# An updated inventory of sea slugs from Koh Tao, Thailand, with notes on their ecology and a dramatic biodiversity increase for Thai waters

**DOI:** 10.3897/zookeys.1042.64474

**Published:** 2021-06-09

**Authors:** Rahul Mehrotra, Manuel A. Caballer Gutiérrez, Chad M. Scott, Spencer Arnold, Coline Monchanin, Voranop Viyakarn, Suchana Chavanich

**Affiliations:** 1 Reef Biology Research Group. Department of Marine Science, Faculty of Science, Chulalongkorn University, Bangkok 10330, Thailand; 2 Aow Thai Marine Ecology Center, Koh Mun Nai, Kram, Klaeng District, Rayong 21110, Thailand; 3 American University of Paris, Department of Computer Science Math and Environmental Science, 6 rue du Colonel Combes, 75007 Paris, France; 4 Muséum national d’Histoire naturelle, 55 rue de Buffon, 75005 Paris, France; 5 Conservation Diver. 7321 Timber Trail Road, Evergreen, Colorado, 80439, USA; 6 Research Center on Animal Cognition (CRCA), Center for Integrative Biology (CBI); CNRS, University Paul Sabatier,Toulouse III, France; 7 Center of Excellence for Marine Biotechnology, Department of Marine Science, Faculty of Science, Chulalongkorn University, Bangkok 10330, Thailand

**Keywords:** Biodiversity exploration, coral reefs, Gulf of Thailand, Heterobranchia, soft sediment habitats

## Abstract

Improved access to field survey infrastructure throughout South-East Asia has allowed for a greater intensity of biodiversity surveys than ever before. The rocky bottoms and coral reef habitats across the region have been shown to support some of the highest sea slug biodiversity on the planet, with ever increasing records. During the past ten years, intensive SCUBA surveys have been carried out at Koh Tao, in the Gulf of Thailand, which have yielded remarkable findings in sea slug biology and ecology. In this work a brief history of sea slug biodiversity research from Thailand is covered and a complete inventory of sea slugs from Koh Tao, Gulf of Thailand is provided. This inventory is based on surveys from 2012 to 2020, with previously unreported findings since 2016. Habitat specificity and species-specific ecology are reported where available with a focused comparison of coral reef habitats and deeper soft-sediment habitats. The findings contribute 90 new species records for Thai waters (92 for the Gulf of Thailand) and report a remarkable consistency in the proportional diversity found to be exclusive to one habitat type or another. Additionally, taxonomic remarks are provided for species documented from Koh Tao that have not been discussed in past literature from Thailand, and a summary of previous records in the Indo-West Pacific is given.

## Introduction

Contemporary sea slug research is largely dominated by investigations into biochemistry, taxonomy, and systematics of the vast diversity of species currently known. Broader aspects remain largely understudied such as development, trophic ecology, and biogeography. Recent years have seen a dramatic increase in the abundance of biodiversity inventories, particularly from regions where much of this work had been sparse before. The importance of documenting local and regional species ranges and diversity is often overlooked despite such studies contributing to our understanding of large-scale environmental issues such as increasing ocean temperatures (Nimbs et al. 2016; Goddard et al. 2018; Ekimova et al. 2019). The problem of invasive species too relies heavily on understanding native and non-native species ranges (Zenetos et al. 2010; Nimbs and Smith 2018). It is thus vital that efforts be made to increase localised biodiversity monitoring, particularly at a time where the rate of change in terrestrial and marine environments is unprecedented and may drive significant biodiversity change and loss (Cowie et al. 2017). The documentation and understanding of baselines in localised species diversity allow for more accurate understanding of biodiversity change both spatially and across time (Nimbs and Smith 2018).

Among the earliest records of sea slugs from Thailand were provided by Bergh (1902) via the ‘Danish Expedition to Siam 1899–1900’ in which he provided records of 22 species (1 Sacoglossa, 3 Cephalaspidea, 4 Aplysiida, and 14 Nudibranchia), all from the Gulf of Thailand. Few additional records were made over the next century, notable inclusions being those of Jensen (1989, 1992), Brunckhorst (1993), and Swennen (1997). These were summarised with more records added in the first review of sea slugs from Thailand by Jensen (1998) and later numerous additional records were provided by Swennen et al. (2001). Thus, in the century since Bergh’s first documentation, the documented diversity in Thai waters reached 81 species, with 46 being recorded within the Gulf of Thailand. However, numerous species documented by Swennen et al. (2001) were only known from shells or remnants and therefore little ecological information could be gained. The waters of Thailand are extremely well suited for comparative investigations between the Indian Ocean and Western Pacific biodiversity and ecology. The Gulf of Thailand has, however, been consistently reported to host a lower diversity of marine life (i.e., Putchakarn and Sonchaeng 2004; Chanmethakul et al. 2010; Wallace et al. 2012), and is therefore often subject to less intensive sampling efforts and in fewer sampling sites. This trend extends to sea slugs with the most extensive review of nudibranch taxa in Thailand being conducted by Chavanich et al. (2013). Their work increased the known biodiversity and biogeography of sea slugs within Thai waters and documented a far greater diversity of taxa on the Andaman coast than the Gulf.

A dedicated survey effort was carried at the island of Koh Tao in the Gulf of Thailand which combined citizen science efforts with in-situ survey techniques and resulted in a dramatic increase in the documented diversity for the Gulf (Mehrotra and Scott 2016). Much of this increase was attributed to previously unexplored soft sediment habitats beyond the slope of fringing coral reefs, which appeared to host a high diversity of species that were not seen in shallower habitats nearer to coral reefs. Subsequent surveys in these habitats revealed a number of novel species descriptions and ecological features that had hitherto been overlooked (Mehrotra et al. 2017, 2019, 2020b). In the present work we summarise the findings of dedicated surveys subsequent to these studies and document a further expansion in sea slug biodiversity in Thai waters. In doing so and by providing a full inventory with known ecology of all species recorded at Koh Tao, we aim to provide a comprehensive baseline on the diversity and ecology of sea slugs in the region.

## Materials and methods

### Surveys and sampling

**Figure 1.**
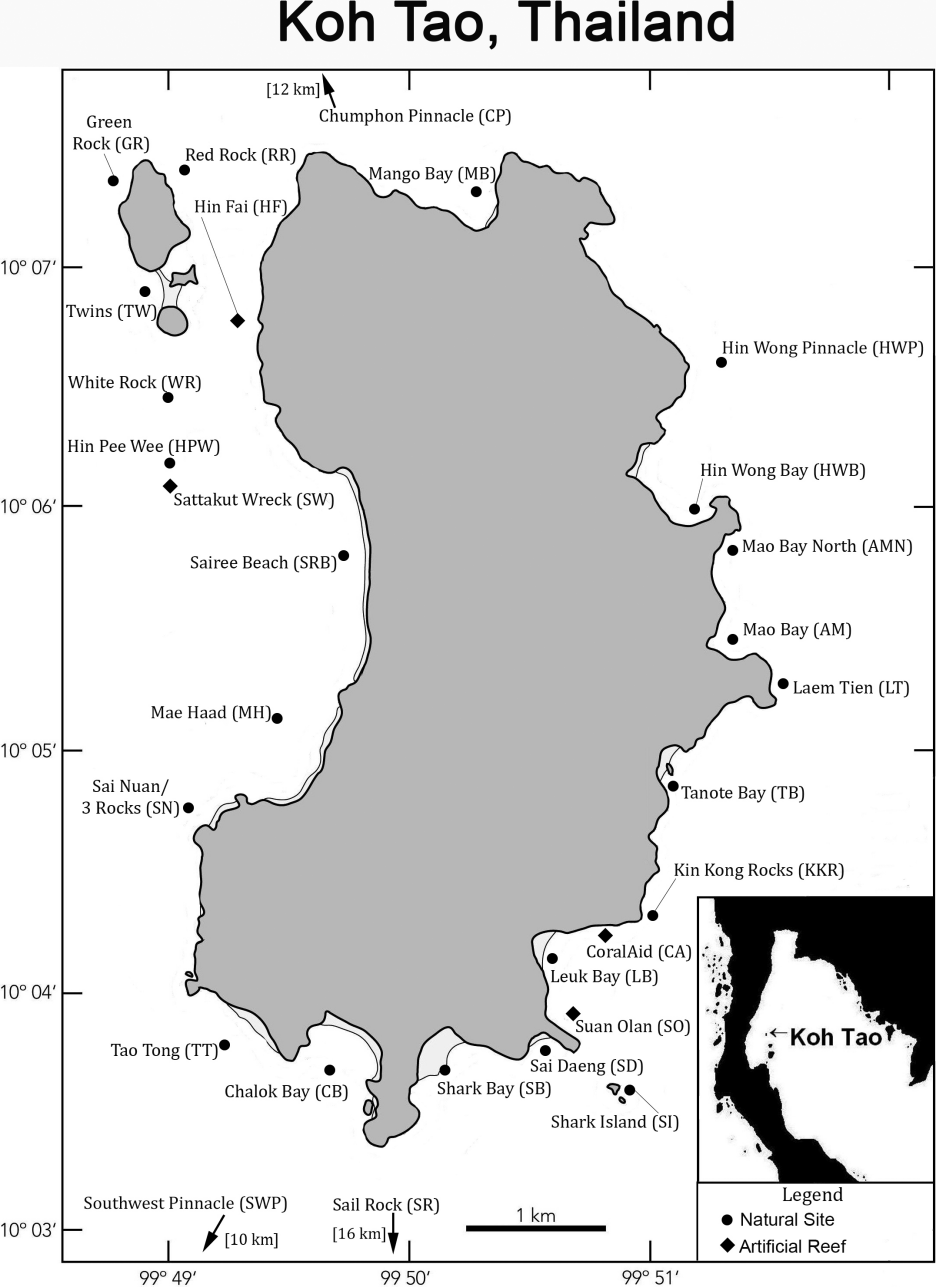
Map of Koh Tao showing surveyed locations in reference to material examined. The inset of the Gulf of Thailand shows the location of Koh Tao.

Benthic surveys were carried out using SCUBA at Koh Tao, Thailand, between January 2016 and February 2020. Roving-diver surveys were performed over both coral reef and soft sediment habitats. Belt transect surveys were also executed across both habitats following the Ecological Monitoring Protocol according to Scott (2012). The majority of surveys were done during the daytime, with a few night-time surveys achieved sporadically throughout the period. Survey intensity ranged from two to 16 people per survey and were carried out through most of the year. During the monsoon season (October–January), survey intensity was reduced to zero surveys per month in some months but were usually between ten to 20 surveys per month throughout the rest of the year. Surveys were carried out throughout the island coast and surrounding pinnacles (Fig. 1). Approximate coordinates for each site are provided in Table 1. As part of these documented surveys, approximately 9200 individual sea slug sightings were recorded across all habitats. Surveys were carried out in equal measure on the coral reef (dominated by scleractinian hard coral substrate) and soft sediment areas.

**Table 1.** Surveyed sites with location codes given in each species after specimen size.

Location	Code	Coordinates
Leuk Bay	LB	10°4'11.65"N, 99°50'34.42"E
Suan Olan Artificial Reef	SO	10°4'6.70"N, 99°50'26.29"E
Coral-Aid Artificial Reef	CA	10°4'20.96"N, 99°50'31.84"E
Shark Island	SI	10°3'41.20"N, 99°50'40.54"E
Sai Daeng	SD	10°3'49.43"N, 99°50'23.80"E
Shark Bay	SB	10°3'39.75"N, 99°50'4.43"E
Chalok Bay	CB	10°3'44.77"N, 99°49'30.35"E
Tao Tong	TT	10°3'58.13"N, 99°49'4.76"E
Sai Nuan and Three Rocks	SN	10°4'45.02"N, 99°48'45.23"E
Mae Haad	MH	10°5'22.53"N, 99°49'14.07"E
Sairee Beach	SRB	10°6'0.99"N, 99°49'15.89"E
Hin Pee Wee	HPW	10°6'19.94"N, 99°48'47.73"E
Sattakut Wreck	SW	10°6'16.97"N, 99°48'47.52"E
White Rock	WR	10°6'27.94"N, 99°48'48.98"E
Hin Fai Artificial Reef	HF	10°6'43.42"N, 99°49'7.18"E
Twins	TW	10°7'1.93"N, 99°48'44.26"E
Green Rock	GR	10°7'31.24"N, 99°48'49.57"E
Red Rock	RR	10°7'19.92"N, 99°48'55.31"E
Mango Bay	MB	10°7'22.52"N, 99°50'5.06"E
Hin Wong Pinnacle	HWP	10°6'47.51"N, 99°51'1.95"E
Hin Wong Bay	HWB	10°6'12.30"N, 99°50'58.63"E
Mao Bay North	AMN	10°5'51.85"N, 99°51'7.69"E
Mao Bay	AM	10°5'32.95"N, 99°51'9.29"E
Laem Thien	LT	10°5'19.13"N, 99°51'17.64"E
Tanote Bay	TB	10°5'1.47"N, 99°50'57.50"E
King Kong Rocks	KKR	10°4'30.25"N, 99°50'46.46"E
Chumphon Pinnacle	CP	10°10'20.52"N, 99°46'44.49"E
Southwest Pinnacle	SWP	9°59'56.22"N, 99°46'44.28"E
Sail Rock	SR	9°56'42.47"N, 99°59'26.46"E

**Figure 2.**
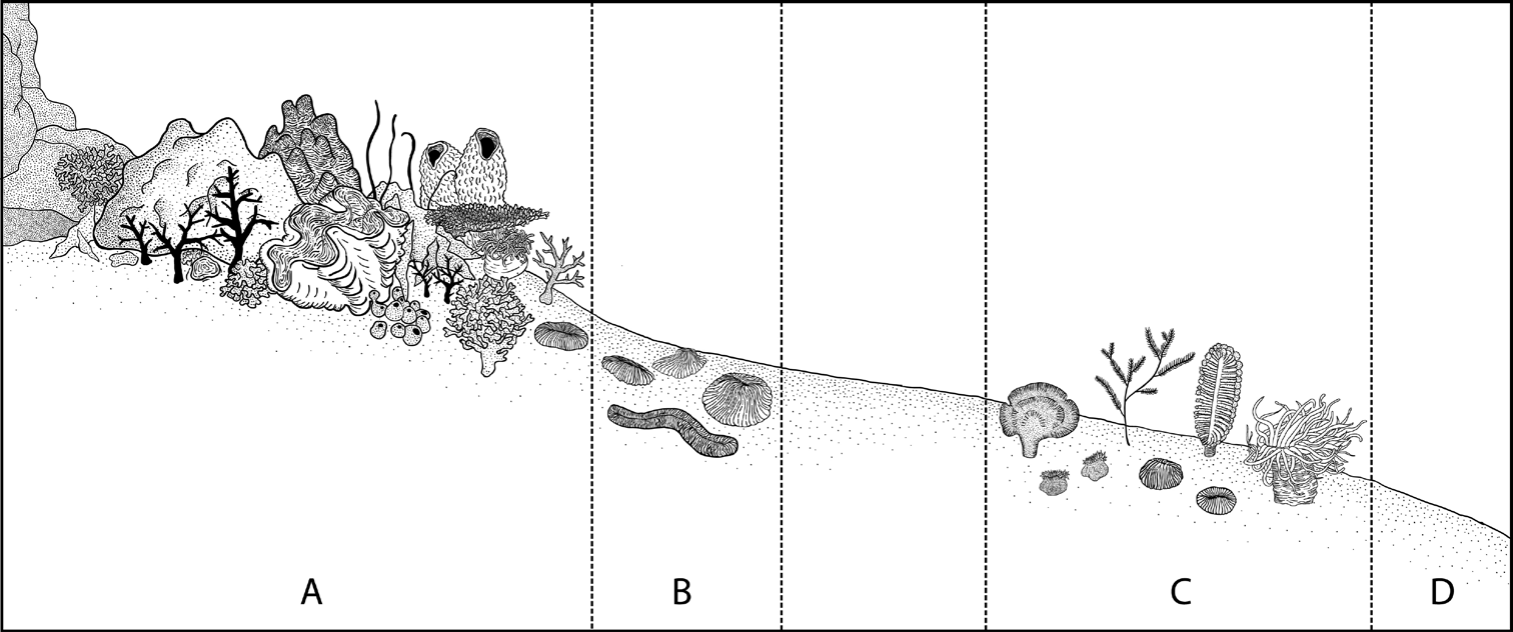
Schematic outline of benthic zones classified in the surveys conducted at Koh Tao **A** fringing reef slope, usually near-shore **B** reef edge as determined by drastic reduction in reef-building scleractinian abundance, leading to zone of no stable substrate nor any Scleractinia**C** soft sediment habitats characterised by sand/silt dominated substrates and colonised by organisms absent/extremely rare in zones **A** and **B**. **D** Deepening of soft sediment slope resulting in a drastic reduction but not absence of soft-sediment colonisers. Illustrated by Pau Urgell Plaza.

The soft sediment habitats, located outside coral reefs, had a surface substrate composition of > 90% sand or silt particles. These are typically large areas lacking natural solid substrates and are therefore deficient in rugosity and stability over longer periods (discussed below). Areas of sandy substrate within or in close proximity to coral-dominated areas were included as coral reef (Fig. 2). In the coral reef regions, the authors surveyed in particular among coral rubble, aforementioned sandy patches, and the underside of dead Fungiidae (Anthozoa: Hexacorallia: Scleractinia) corals. Previous surveys (Mehrotra and Scott 2016) had preliminarily suggested that closer examination of these areas could yield a host of cryptic taxa. Soft sediment areas with close proximity to coral reef habitats, and those with a substrate composition of > 10% hard substrate (particle sizes or corals > ca. 5 cm), were considered as coral reef or reef edge for the surveys and were assessed visually. Thus, there was always clear separation between habitats considered to be coral reef and the deeper soft sediment habitats (referred to here simply as soft sediments). Indicators for soft sediment habitats based on our definitions included organisms that have already been found to grow exclusively in these areas such as sea pens, specific macroalgal species, and specific free-living Scleractinia among others (Mehrotra et al. 2017, 2019).

For each documented species, a small number of specimens was examined closely for taxonomic purposes, with the vast majority of subsequent specimens recorded being noted for their ecology or simply their presence. Detailed specimen examination was carried out in-situ where possible or after sampling using high-magnification underwater photography. Ex-situ examination was carried externally on live specimens which were collected by hand and subsequently returned to their original habitats. All living specimens studied are here documented as ‘material examined’. Specimens were externally identified by the authors aided by in-situ photographs based on relevant literature and contrasted with known species prevalence in Thai waters (see Table 2). Additionally, taxon validity was confirmed with the most recent literature and assisted in part with the World Register of Marine Species (WoRMS 2021) and the references contained within.

**Table 2.** List of published literature between 1989–2020 in which sea slug diversity and distribution records in Thai waters are contributed.

References	Gulf of Thailand diversity	Total Thailand diversity
Jensen 1989	19	20
Jensen 1992	19	24
Brunckhorst 1993	19	38
Gosliner and Johnson 1994	20	39
Jensen 1998; Swennen 1997	28	63
Swennen 2001; Swennen et al. 2001	46	81
Jensen 2007; Swennen 2007; Robba et al. 2007	49	88
Swennen and Buatip 2009	49	88
Nabhitabhata 2009	50	100
Swennen 2011	51	101
Swennen and Buatip 2012	52	102
Chavanich et al. 2013	111	203
Jensen et al. 2014a	111	204
Mehrotra and Scott 2016	154	239
Martynov et al. 2019; Korshunova et al. 2019	156	241
Mehrotra et al. 2020a, b; Wang et al. 2020	160	245
Present study	256	336

**Figure 3.**
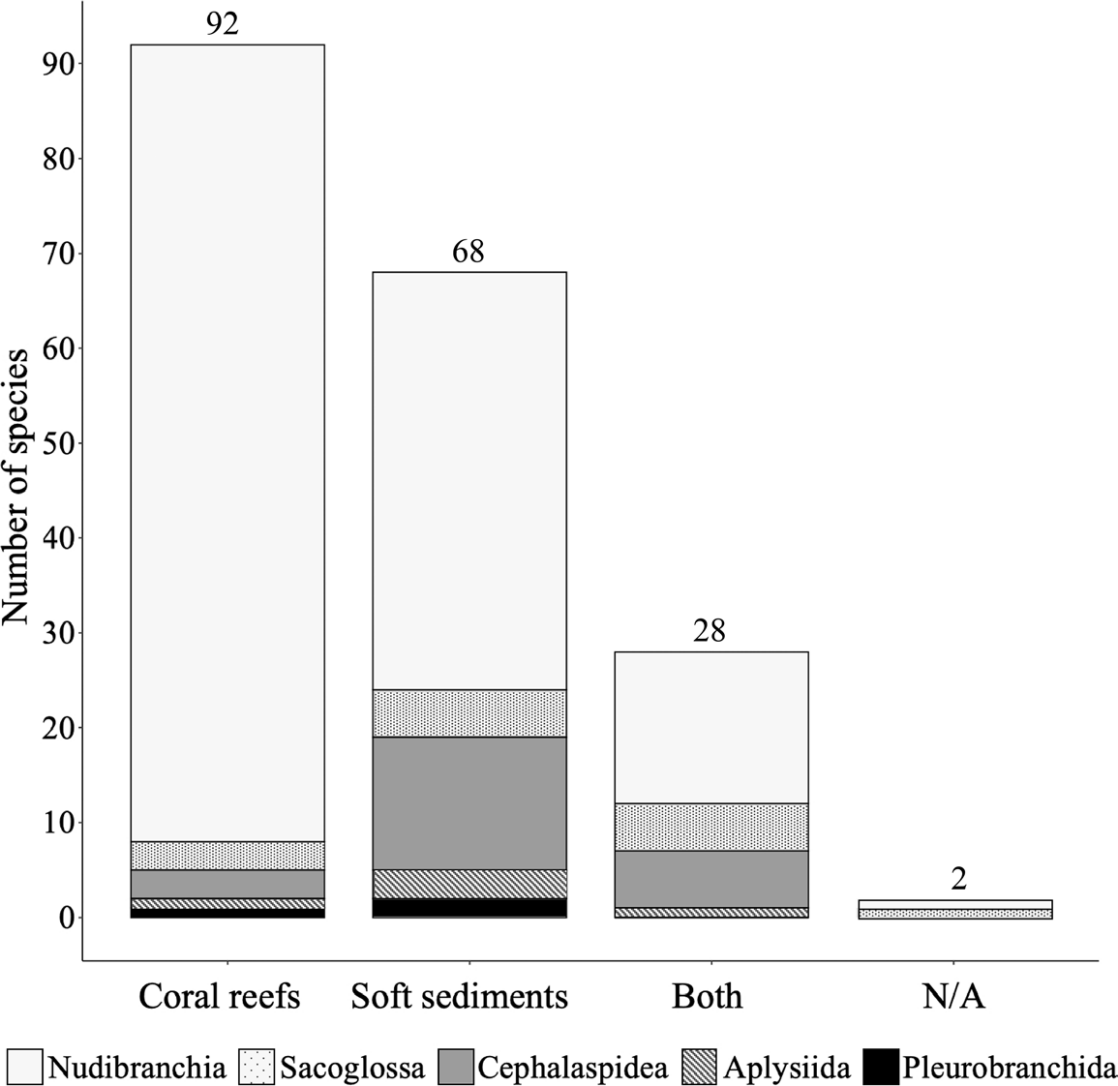
Habitat specificity of the major sea slug groups at Koh Tao. Number of species exclusively found at each habitat type at Koh Tao, compared with the number of species recorded from both habitat types. Two species without a confirmed benthic habitat type were recorded as N/A.

## Results

In total, 191 species of heterobranch sea slugs have been documented from Koh Tao to date across the orders Sacoglossa (14 species), Cephalaspidea (23 species), Aplysiida (5 species), Pleurobranchida (3 species), and Nudibranchia (146 species). These results more than double the known taxa from Koh Tao (new records for Koh Tao identified below by an asterisk *) and contribute a further 90 first records for Thai waters (96 for the Gulf of Thailand) from the island (not including the 32 species first documented in Mehrotra and Scott 2016). Approximately half of all recorded species (*N* = 92) were found exclusively within coral reef habitats, with 68 species (36%) being exclusively recorded from the soft sediment habitats and 28 species recorded in both habitats. Two species could not be attributed to a particular benthic habitat type (see remarks for Limapontiidae sp. and *Scyllaea fulva*). A large number of species were found exclusively or in far greater abundance under the skeletons of dead Fungiidae corals (see discussion).

### Systematics

#### Class Gastropoda Cuvier, 1795

**Subclass Heterobranchia Burmeister, 1837**

**Superorder Panpulmonata Jörger, Stöger, Kano, Fukuda, Knebelsberger & Schrödl, 2010**

**Order Sacoglossa Ihering, 1876**

**Family Costasiellidae Clarke, 1984**

##### Genus *Costasiella* Pruvot-Fol, 1951

###### Costasiella cf. kuroshimae

Taxon classificationAnimaliaSacoglossaCostasiellidae

Ichikawa, 1993

2EA91BF2-9233-5FFD-B9F5-EFD728D71AD5

Figure 4A

####### Material examined.

Two specimens 3–6 mm, LB; two specimens 4–6 mm, SN.

####### Ecology.

In soft sediment habitats, beyond the coral reef where it feeds predominantly on *Avrainvillea longicaulis* (Kützing) G. Murray & Boodle, 1889 and less commonly on *Vaucheria* sp. Depth 10–18 m.

####### Distribution.

*Costasiella kuroshimae* is currently known from the Indo-Pacific including the Red Sea (Yonow 2015), Singapore (Jensen 2009), Indonesia (Eisenbarth et al. 2018), Japan (Ichikawa 1993), Guam (Jensen et al. 2014b), Madagascar, Tanzania, Malaysia, Papua New Guinea, Palau, and Australia (Gosliner et al. 2008). Known from the Gulf waters of Thailand (Mehrotra and Scott 2016).

####### Remarks.

Due to the original description of the species being entirely based on external features, the identity of numerous similar species and the extent of the variability of the species has remained unclear for several years. Molecular work (Jensen et al. 2014b) has shown that specimens identified as *Costasiella kuroshimae* or C. cf. kuroshimae actually make up numerous distinct species that currently await description. Research carried out on specimens from Koh Tao (Mehrotra et al. 2019) indicates that this species is palatable to some scleractinian corals and, based on natural observations, may be viable prey for the free-living coral *Heteropsammia cochlea* (Spengler, 1781), in soft sediment habitats.

**Figure 4.**
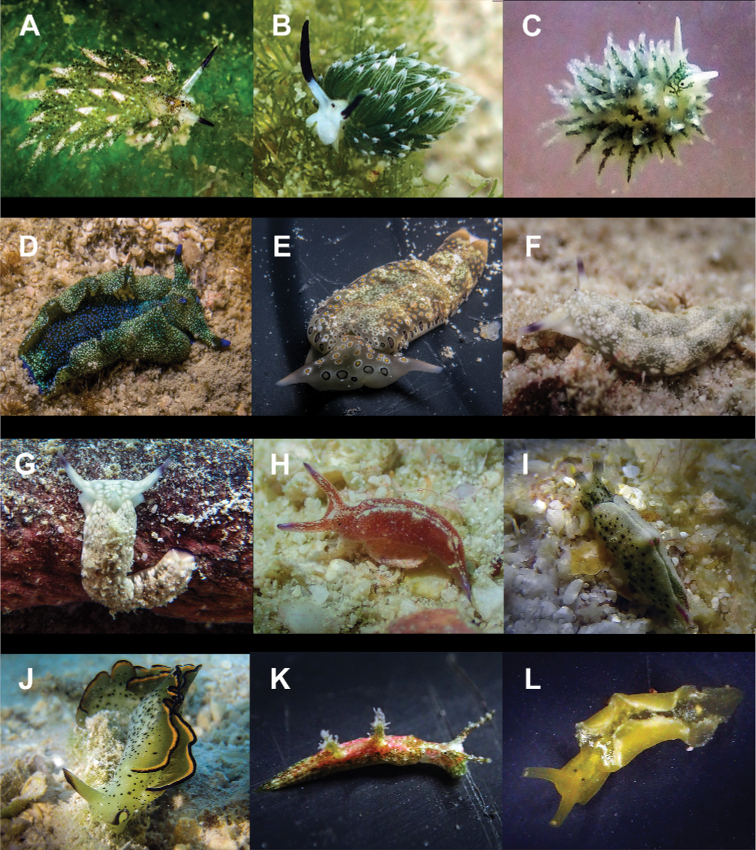
**A**Costasiella cf. kuroshimae 6 mm **B***Costasiella usagi* 6 mm **C**Limapontiidae sp. 3 mm **D***Plakobranchus noctisstellatus* 21 mm (photograph by Pau Urgell Plaza) **E***Plakobranchus ocellatus* 32 mm **F***Plakobranchus papua* 27 mm **G***Plakobranchus papua* 21 mm on *Holothuria edulis***H***Elysia aowthai* 12 mm **I***Elysia asbecki* 15 mm **J**Elysia cf. marginata 70 mm **K***Elysia mercieri* 12 mm **L***Elysia obtusa* 9 mm.

###### Costasiella usagi

Taxon classificationAnimaliaSacoglossaCostasiellidae

Ichikawa, 1993

4089F648-212C-5375-8744-FFFE0922CEB5

Figure 4B

####### Material examined.

Three specimens 2–6 mm, LB.

####### Ecology.

In soft sediment habitats beyond the coral reef where it feeds predominantly on *Avrainvillea longicaulis* and less commonly on *Vaucheria* sp. An individual of the species has been observed to be naturally captured and ingested by the free-living coral *Heteropsammia cochlea* with subsequent investigations suggesting the species may represent viable prey for the coral in soft sediment habitats (Mehrotra et al. 2019). Depth 10–18 m.

####### Distribution.

Across the Indo-Pacific including India (Dixit et al. 2017), Singapore (Jensen 2009), Japan (Ichikawa 1993), Malaysia, the Philippines, Papua New Guinea, Australia, and Guam (Gosliner et al. 2008). Known from the Gulf waters of Thailand (Mehrotra and Scott 2016).

#### Family Limapontiidae Gray, 1847

##### * Limapontiidae sp.

Taxon classificationAnimaliaSacoglossaLimapontiidae

1A74BF44-4E67-518F-AA2C-CB0266CCB253

Figure 4C

###### Material examined.

One specimen 3 mm, location unknown.

###### Ecology.

Local ecology is unknown.

###### Distribution.

Unknown.

###### Remarks.

Similar to *Ercolania translucens* Jensen, 1993 or *Stiliger* sp. 7 in Gosliner et al. (2018) in having cerata with elongated white apices and green pigment on the head not extending to the white rhinophores. The specimen was observed in a holding tank several days after a broad sampling effort of multiple algal species from Koh Tao. Algae were collected from several locations at the south of the island and although they had been checked carefully, the cryptic nature of the species allowed it to be overlooked. The species was recorded only once in April 2015 and has not been recorded since. The internal anatomy of the specimen was not studied and thus it could not be identified to genus level.

#### Suborder Plakobranchacea Gray, 1840

**Superfamily Plakobranchoidea Gray, 1840**

**Family Plakobranchidae Rang, 1829**

##### Genus *Plakobranchus* van Hasselt, 1824

###### Plakobranchus noctisstellatus

Taxon classificationAnimaliaSacoglossaPlakobranchidae

Mehrotra, Caballer, Scott, Arnold, Monchanin & Chavanich, 2020

5AA8A540-454C-5DC5-AE72-AC8079CE7EC3

Figure 4D

####### Material examined.

One specimen 28 mm, SN; two specimens 26–31 mm, TT.

####### Ecology.

From deeper soft sediments outside coral reef habitats. Depth 15–25 m.

####### Distribution.

Vanuatu, Indonesia, Papua New Guinea (Gosliner et al. 2008, 2015) and the Gulf of Thailand (Mehrotra et al. 2020b).

###### Plakobranchus ocellatus

Taxon classificationAnimaliaSacoglossaPlakobranchidae

van Hasselt, 1824

7BE7BEE9-F247-534A-BF9D-C451DEBCCEF4

Figure 4E

####### Material examined.

Three specimens 25–32 mm, CB.

####### Ecology.

From shallow soft sediments to sandy areas along the reef edge. Rarely in deeper soft sediment habitats beyond the reef edge. Depth 0.5–11 m.

####### Distribution.

*Plakobranchus ocellatus* and P. cf. ocellatus are currently considered widespread across the Indo-Pacific including Kenya, Zanzibar, the Red Sea, Maldives, Seychelles, Réunion (Yonow 2012), India (Sheeja and Padma Kumar 2014), the Philippines (Christa et al. 2012), Indonesia (Eisenbarth et al. 2018; Yonow and Jensen 2018), Japan (Maeda et al. 2012), Australia, Papua New Guinea (Yonow and Jensen 2018), Guam (Wägele et al. 2011), Vanuatu (Krug et al. 2013), Hawaii (Wade and Sherwood 2016), Tanzania, Madagascar, Malaysia and Palau (Gosliner et al. 2008). Specimens considered as *P. ocellatus* have been previously recorded from the Andaman and Gulf waters of Thailand (Jensen 1992; Nabhitabhata 2009).

####### Remarks.

Specimens from Koh Tao were recently reviewed by Mehrotra et al. (2020b).

###### Plakobranchus papua

Taxon classificationAnimaliaSacoglossaPlakobranchidae

Meyers-Muñoz & van der Velde in Meyers-Muñoz et al., 2016

6E0DA2CA-169E-58CB-8ADF-F577ED88F89E

Figure 4F, G

####### Material examined.

Three specimens 19–30 mm, SN.

####### Ecology.

Abundant in shallow soft sediment habitats and among the corals and soft sediments of the reef edge. Uncommon, but present in dense coral reef habitats. Rare in deeper soft sediment habitats outside the coral reef. Has been observed being ingested naturally by the scleractinian coral *Pleuractis paumotensis* (Stutchbury, 1833) but is mostly considered unpalatable by such corals (Mehrotra et al. 2015, 2019). During daytime surveys, a single observation was made of *P. papua* crawling upon the sea cucumber *Holothuria edulis* Lesson, 1830 (Fig. 4G), which may have been considered unremarkable were it not for the findings of Mercier and Hamel (2005). Depth 1–19 m.

####### Distribution.

Known only from the Philippines, Malaysia, Indonesia, and Papua New Guinea (Meyers-Muñoz et al. 2016; Yonow and Jensen 2018). Known from Gulf waters of Thailand (Mehrotra et al. 2020b).

####### Remarks.

Specimens from Koh Tao were recently reviewed (Mehrotra et al. 2020b).

###### Elysia aowthai

Taxon classificationAnimaliaSquamataPlakobranchidae

Mehrotra, Caballer, Scott, Arnold, Monchanin & Chavanich, 2020

28ABFC57-1E3F-5373-8092-29AD38F07E87

Figure 4H

####### Material examined.

One specimen 14 mm, LB; one specimen 16 mm, TT.

####### Ecology.

From deeper soft sediments outside coral reef habitats. Depth 10–24 m.

####### Distribution.

Guam, Australia, and the Gulf of Thailand (Mehrotra et al. 2020b).

##### Genus *Elysia* Risso, 1818

###### Elysia asbecki

Taxon classificationAnimaliaSquamataPlakobranchidae

Wägele, Stemmer, Burghardt & Händeler, 2010

4EC8315D-3409-5FA3-A18F-97134E11E0BB

Figure 4I

####### Material examined.

One specimen 15 mm, HF; one specimen 23 mm, TW.

####### Ecology.

In coral reef habitats throughout the island. Depth 3–18 m.

####### Distribution.

Australia, Samoa (Wägele et al. 2010), the Philippines, Indonesia, Papua New Guinea, Japan, Guam, and Hawaii (Gosliner et al. 2008; Wägele et al. 2010). Known from the Gulf waters of Thailand (Mehrotra and Scott 2016).

###### * Elysia cf. marginata

Taxon classificationAnimaliaSquamataPlakobranchidae

(Pease, 1871)

299F959E-BCA9-563E-960B-462177E8F141

Figure 4J

####### Material examined.

Three specimens 65–82 mm, CB.

####### Ecology.

Mostly recorded from specimens inhabiting a shallow, isolated patch of *Halimeda macroloba* Decaisne, 1841 in soft sediment habits, although presumably feeding on other nearby algae. Also observed from shallow coral reef habitats, rarely. Depth 0.5–6 m.

####### Distribution.

*Elysia marginata* is at present recorded from the Indo-Pacific including Myanmar (Sanpanich and Duangdee 2019), Vietnam (Martynov and Korshunova 2012), Indonesia (Yonow and Jensen 2018), Australia (Nimbs and Smith 2016), Japan, Guam, French Polynesia, Vanuatu, and Hawaii (Krug et al. 2013). Specimens from South Africa, Madagascar, and Réunion (Gosliner et al. 2008 as *Elysia ornata*) also are likely to correspond to this complex. Known from the Andaman waters of Thailand (Jensen 1992), here representing a first record for the Gulf of Thailand.

####### Remarks.

Recent molecular investigations (Krug et al. 2013) have indicated up to four possible clades making up the species *Elysia marginata*, that was separated from its Caribbean counterpart *Elysia ornata* (Swainson, 1840), which was formerly considered circumtropical. Yonow and Jensen (2018) further discuss the challenges in assigning all specimens with the ‘characteristic’ orange and black marginal bands on the parapodia to *E. marginata* as similar species such as *E. faustula* Bergh, 1871 and *E. grandifolia* Kelaart, 1858 were described and illustrated with comparable features. Both aforementioned species differ in ground colour or the presence/absence of denticulation on radular teeth, and both of these features have been shown to be variable within a single species and often a single specimen (Mehrotra et al. 2020b). Therefore, all indications point to a need for a comprehensive analysis integrating morphology, ecology, and molecular data to delineate species in this complex.

###### * Elysia mercieri

Taxon classificationAnimaliaSquamataPlakobranchidae

(Pruvot-Fol, 1930)

234A888B-B6A0-520C-A10B-7EC4F9C5A77F

Figure 4K

####### Material examined.

One specimen 12 mm, SO.

####### Ecology.

Found upon concrete artificial reefs in soft sediment habitats that formed part of coral restoration efforts. Depth 11–14 m.

####### Distribution.

Across the Indo-Pacific including the Red Sea (Yonow 2015), Indonesia (Eisenbarth et al. 2018), Japan (Trowbridge et al. 2011), Mariana Islands (Carlson and Hoff 2003), Malaysia, Papua New Guinea, New Caledonia, and Guam (Gosliner et al. 2008). Here representing a first record for Thai waters.

####### Remarks.

*Elysia mercieri* is known to be predated upon by the nudibranch *Gymnodoris okinawae* Baba, 1936 (Nakano and Hirose 2011).

###### * Elysia obtusa

Taxon classificationAnimaliaSquamataPlakobranchidae

Baba, 1938

3AA5F008-E3CF-5E61-86C9-AE12D1DCC10A

Figure 4L

####### Material examined.

One specimen 9 mm, CB; one specimen 12 mm, TW.

####### Ecology.

Among rubble, particularly found underneath the skeletons of dead Fungiidae corals, in shallow coral reef habitats. No association with prey was observed but is known to be part of a group of species feeding on the alga *Bryopsis* (Krug et al. 2016). Depth 3–8 m.

####### Distribution.

Across the Indo-Pacific including India (Apte et al. 2010), Taiwan (Huang et al. 2016), Hong Kong (Jensen 2003), Japan (Trowbridge et al. 2011), Australia (Nimbs and Smith 2016), Samoa (Wägele et al. 2010), Madagascar, Malaysia, Papua New Guinea, the Philippines, Korea, Guam, Marshall Islands, and Hawaii (Gosliner et al. 2008 as *Elysia flava* Verrill, 1901). Here representing a first record for Thai waters.

####### Remarks.

Both individuals recorded were found adhering to the underside of dead fungiid skeletons. The species is locally rare and here included as a first record for the Gulf of Thailand and Thai waters in general.

###### * Elysia pusilla

Taxon classificationAnimaliaSquamataPlakobranchidae

(Bergh, 1871)

217CD247-786C-53A3-807D-0524288C191C

Figure 6A

####### Material examined.

Three specimens 3–7 mm, CB; one specimen 4 mm, SRB.

####### Ecology.

Feeds on *Halimeda macroloba*, on which it is highly cryptic. Host and prey found in soft sediment habitats near coral reefs between 0.5 and 9 m depth. Multiple individuals may be found feeding on a single prey item. Populations of the host algae *H. macroloba* have been found at only two locations at the island, 6–9 m depth at SRB and a small intertidal patch at site CB at 0.5–1.5 m. The abundance of *E. pusilla* has been found to be greater at CB than on the larger but less dense population of *H. macroloba* at site SRB.

####### Distribution.

Widespread across the Indo-Pacific including the Red Sea (Yonow 2008), Réunion (Bourjon et al. 2018), India (Sreeraj et al. 2012), Singapore (Jensen 2009), Vietnam (Martynov and Korshunova 2012), Indonesia (Eisenbarth et al. 2018), Australia (Nimbs and Smith 2016), Japan, Guam (Vendetti et al. 2012), Mexico (Goddard and Hermosillo 2008), Costa Rica (García-Méndez and Camacho-García 2016), South Africa, Tanzania, Madagascar, New Caledonia, and Hawaii (Gosliner et al. 2008). Known from the Andaman waters of Thailand (Jensen 1992), and here representing a first record for the Gulf of Thailand.

####### Remarks.

The status of *Elysia pusilla* and its taxonomic implications for the genus needs closer investigation (Jensen 2009, 2015; Krug et al. 2016). Recent observations carried out by Mehrotra et al. (2019) indicate that *E. pusilla* specimens from Koh Tao are considered palatable to opportunistic scleractinian coral predators and are readily consumed by these reef building corals. However, to date, no instances of natural prey capture of *E. pusilla* by these corals has been documented.

###### * Elysia cf. tomentosa

Taxon classificationAnimaliaSquamataPlakobranchidae

Jensen, 1997

3C35222C-ABE2-5935-B24E-DF962D7417B1

Figure 6B

####### Material examined.

One specimen 37 mm, CB.

####### Ecology.

In soft sediment habitats outside the coral reef. Observed feeding on a pinnate form of *Caulerpa racemosa* (Forsskål) J. Agardh, 1873 which is found chiefly in soft sediment habitats beyond the coral reef. A more lenticular/globular form of the algae can be abundant in some shallow, degraded reef habitats; however, specimens from Koh Tao not been observed associated with this variety. Depth 12–20 m.

####### Distribution.

At present *Elysia tomentosa* is considered widespread across the Indo-Pacific (but see remarks below) including Iran (Oladi et al. 2018), Madagascar, Malaysia (Gosliner et al. 2008), Australia (Jensen 1997), Réunion, India, Singapore, the Philippines, Indonesia, Japan, New Caledonia, and Hawaii (Jensen 2015). Likely introduced to the Mediterranean alongside the highly invasive *Caulerpa* spp. (Zenetos et al. 2010). Here representing a first record for Thai waters.

####### Remarks.

Recent molecular investigations have found that specimens recognised as *Elysia tomentosa* likely correspond to a complex of at least six species (Krug et al. 2013). Discussions about the identity of specimens identifiable by black marginal lines along the parapodia and the similar species *Elysia expansa* (O’Donoghue, 1924) have yet to be resolved (Rudman 2009a; Krug et al. 2013). Additionally, Oladi et al. (2018) recently documented a species in this complex from Iran that matches a sequence of E. cf. tomentosa from the Andaman coast of Thailand, by Cornelius Swennen (GenBank accession number KC573755.1); however, no record of this species from the waters of Thailand has been found in the literature to date. Greater sampling efforts from more locations, with an assessment of internal characters, are needed to clarify this complex, including specimens from Koh Tao.

##### Genus *Thuridilla* Bergh, 1872

###### Thuridilla cf. gracilis

Taxon classificationAnimaliaSacoglossaPlakobranchidae

(Risbec, 1928)

A2AD99A3-22DD-5AD9-AF01-9EBED097B161

Figure 6C

####### Material examined.

1 specimen 18 mm, HWB; 2 specimens 8–15 mm, LB.

####### Ecology.

Found in coral reef habitats throughout the island. Depth 2–25 m.

####### Distribution.

*Thuridilla gracilis* sensu lato is known from Maldives, Seychelles (Yonow 2012), India (Apte 2009), Singapore (Jensen 2009), Myanmar (Sanpanich and Duangdee 2019), Indonesia (Yonow and Jensen 2018), Vietnam (Martynov and Korshunova 2012), Taiwan (Huang et al. 2016), Japan (Trowbridge et al. 2011), Australia (Nimbs and Smith 2016), Madagascar, Malaysia, the Philippines, Papua New Guinea, Palau, New Caledonia, Guam, and Fiji (Gosliner et al. 2008). Known from the Andaman and Gulf waters of Thailand (Jensen 1992; Nabhitabhata 2009).

####### Remarks.

**Figure 5.**
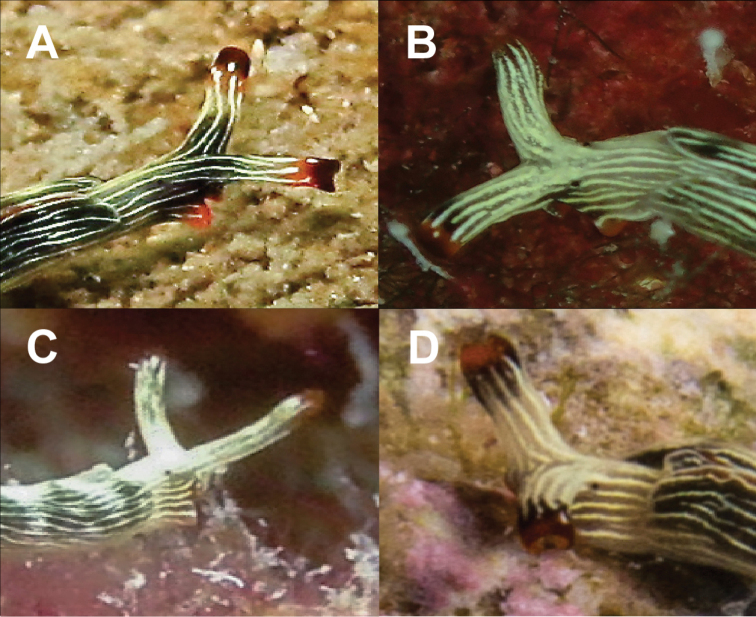
Variation in the heads of Thuridilla cf. gracilis at Koh Tao. Specimens 14 mm (**A**), 18 mm (**B**), 9 mm (**C**), 16 mm (**D**).

The taxonomic status of *Thuridilla gracilis* is at present unclear. Recent works (Yonow and Jensen 2018; Papu et al. 2020) have highlighted the significant variability of specimens ascribed to the species and questioned the synonymisation of multiple white-striped species of *Thuridilla* (Gosliner 1995; Rudman 2000b) under the single species *T. gracilis*. Indications suggest that the breadth of specimens currently considered as *T. gracilis* encompass a complex of species (Händeler and Wägele 2007; Yonow and Jensen 2018; Papu et al. 2020) that requires a comprehensive analysis utilising both morphological and molecular means. Specimens from Koh Tao do not possess any blue markings, instead being closer to the original description and illustration of *T. gracilis* in possessing a thin orange-red marginal band along the parapodia that meets medially and having the white lines often extending to the tips of the rhinophores. Papu et al. (2020) provided photographs highlighting the external variability on the heads of specimens from Bangka, Indonesia suggesting possible diagnostic value. As such, we have emulated this and provided the range of variation visible in the heads of adult specimens from Koh Tao (Fig. 5).

#### Order Cephalaspidea P. Fischer, 1883

**Superfamily Cylichnoidea H. Adams & A. Adams, 1854**

**Family Colinatydidae Oskars, Bouchet & Malaquias, 2015**

##### Genus *Colinatys* Ortea, Moro & Espinosa, 2013

###### * Colinatys sp.

Taxon classificationAnimaliaCephalaspideaColinatydidae

994D3877-8295-5897-A10A-AF3CBB17B9DC

Figure 6D

####### Material examined.

One specimen 3 mm, CB.

####### Ecology.

Endobenthic in sand in coral reef habitats. Depth 3–11 m.

####### Distribution.

The genus is currently recognised to be monospecific with *Colinatys alayoi* (Espinosa & Ortea Rato, 2004) known from the Bahamas, Cuba, Florida, and Martinique Island (Romani et al. 2015; Ortea Rato and Buske 2018). Indo-Pacific records are limited to specimens from Hawaii (Oskars et al. 2015) and Japan (SSW 2017) with species identity yet to be confirmed. Here representing a first record of the family from Thai waters.

**Figure 6.**
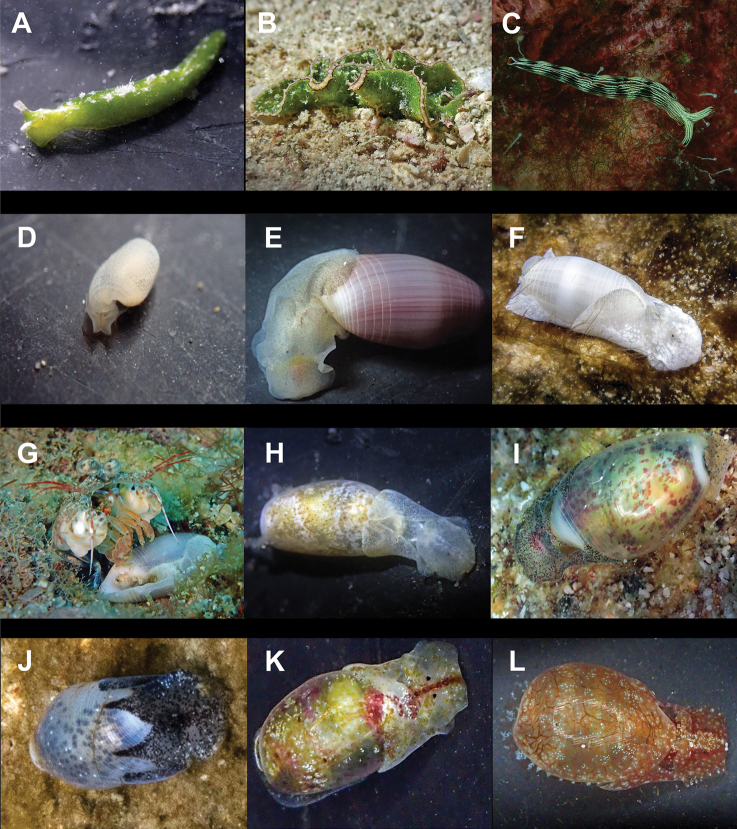
**A***Elysia pusilla* 7 mm **B**Elysia cf. tomentosa 37 mm **C**Thuridilla cf. gracilis 18 mm **D***Colinatys* sp. 3 mm **E***Aliculastrum cylindricum* 30 mm **F***Aliculastrum debilis* 28 mm **G***A. debilis* (shell 12 mm) captured alive and predated by Haptosquilla cf. nefanda**H***Roxaniella multistriata* 9 mm **I***Atys semistriatus* 8 mm **J***Atys* sp. 11 mm **K***Diniatys dentifer* 5 mm **L***Diniatys dubius* 6 mm.

#### Superfamily Haminoeoidea Pilsbry, 1895

**Family Haminoeidae Pilsbry, 1895**

##### Genus *Aliculastrum* Pilsbry, 1896

###### * Aliculastrum cylindricum

Taxon classificationAnimaliaCephalaspideaHaminoeidae

(Helbling, 1779)

08BE1560-7153-5585-B1E2-4DEE2CFB3FDE

Figure 6E

####### Material examined.

Two specimens 25–30 mm, LB.

####### Ecology.

In soft sediment habitats outside the coral reef where it is found associated with cyanobacterial mats on the benthos. May also be found near the reef edge when cyanobacteria abundances increase, indicating a possible seasonal influence in abundance. Often found together with other Haminoeidae species from Koh Tao, apart from *Atys* sp. It may be a probable prey species for the mantis shrimp Haptosquilla cf. nefanda (Kemp, 1911) (see ecology of *Aliculastrum debilis* below). Depth 8–18 m.

####### Distribution.

Widespread across the Indo-Pacific including Mozambique (Macnae and Kalk 1958), Tanzania, Madagascar, the Philippines, Papua New Guinea, New Caledonia (Gosliner et al. 2008), South Africa, Seychelles, Mauritius, India, Japan, China, Fiji, and Tahiti (Too et al. 2014). Known from the Gulf waters of Thailand (Nabhitabhata 2009).

###### Aliculastrum debilis

Taxon classificationAnimaliaCephalaspideaHaminoeidae

(Pease, 1860)

62EEAD7F-37E6-5148-8D58-D5DEF0CA46CC

Figure 6F, G

####### Material examined.

Two specimens 30 mm, LB; one specimen 28 mm, TB.

####### Ecology.

Extremely similar to *A. cylindricum* (see above). Often found together with other Haminoeidae species from Koh Tao, apart from *Atys* sp. As part of the present surveys, observations were made of hunting and capture of specimens of *A. debilis* by the stomatopod Haptosquilla cf. nefanda (Fig. 6G). Captured animals were taken into burrows made by the crustacean. While active feeding was not observed, it should be noted that shells of *A. debilis*, *A. cylindricum*, and rarely *Atys semistriatus* Pease, 1860 can be found in high abundance around the holes of H. cf. nefanda and other mantis shrimps in the soft sediment habitats. These shells are often broken, but not always, and are likely indications of predation by these crustaceans, which are abundant in these habitats. Depth 8–18 m.

####### Distribution.

Across the western Pacific including the Philippines, Guam, Tahiti, Fiji, and Hawaii (Too et al. 2014). Known from the Gulf waters of Thailand (Mehrotra and Scott 2016).

##### Genus *Roxaniella* Monterosato, 1884

###### * Roxaniella multistriata

Taxon classificationAnimaliaCephalaspideaHaminoeidae

(Schepman, 1913)

16D0741C-2B7B-56CB-9A28-912F72BAEFC3

Figure 6H

####### Material examined.

Two specimens 5–9 mm, LB.

####### Ecology.

Very similar to those of *Aliculastrum* spp. though more regularly found immersed within/under cyanobacterial mats than on top. Often found together with other Haminoeidae species from Koh Tao, apart from *Atys* sp. Depth 8–18 m.

####### Distribution.

Known across the Indo-Pacific including Tanzania, the Philippines, Fiji (Gosliner et al. 2008), Indonesia, Palau, Guam, Tahiti (Too et al. 2014), and Hawaii (Kay 1979). Here representing a first record from Thai waters.

##### Genus *Atys* Montfort, 1810

###### * Atys semistriatus

Taxon classificationAnimaliaCephalaspideaHaminoeidae

Pease, 1860

09FEC216-C5C5-5A26-A21E-2965BF001B2D

Figure 6I

####### Material examined.

Two specimens 8–12 mm, LB.

####### Ecology.

Very similar to *R. multistriata*, with which it is often found. A possible prey species for the mantis shrimp Haptosquilla cf. nefanda (see ecology of *Aliculastrum debilis*). Depth 8–18 m.

####### Distribution.

Across the Indo-Pacific including Japan (Kuroda and Habe 1952), Madagascar, Malaysia, the Philippines, Papua New Guinea, Guam, Samoa (Gosliner et al. 2008), Indonesia, New Caledonia, Tahiti, Hawaii (Too et al. 2014), and the Red Sea (Heller and Thompson 1983; Yonow 2008). Here representing a first record for Thai waters.

###### * Atys

Taxon classificationAnimaliaCephalaspideaHaminoeidae

sp.

8488E0EC-A36E-5F7F-86CF-EB504503AEBE

Figure 6J

####### Material examined.

One specimen 11 mm, AMB.

####### Ecology.

In soft sediment habitats outside the coral reef. Depth 23 m.

####### Distribution.

*Atys* sp. 6 (Gosliner et al. 2018) is currently known from the Philippines, Vanuatu, and Indonesia.

##### *Diniatys* Iredale, 1936

###### * Diniatys dentifer

Taxon classificationAnimaliaCephalaspideaHaminoeidae

(A. Adams, 1850)

03B7BE58-2874-582C-876D-D0DEC7DF5DAA

Figure 6K

####### Material examined.

Three individuals 2–5 mm, LB.

####### Ecology.

Very similar to the other soft-sediment associated Haminoeidae such as *Aliculastrum* spp., *R. multistriata* etc., which are often found together. Depth 8–18 m.

####### Distribution.

*Diniatys dentifer* is known from Madagascar, the Philippines, Japan, Indonesia, Papua New Guinea, Guam, Hawaii, French Polynesia (Too et al. 2014), and the Red Sea (Yonow 2008). Here documented as a first record for Thai waters.

###### * Diniatys dubius

Taxon classificationAnimaliaCephalaspideaHaminoeidae

(Schepman, 1913)

DCDB4554-B0C3-5A52-B099-88D761B2B498

Figure 6L

####### Material examined.

Three individuals 3–6 mm, LB.

####### Ecology.

Very similar to *D. dentifer*. Depth 8–18 m.

####### Distribution.

*Diniatys dubius* is known from the Philippines, Indonesia, Papua New Guinea, Guam, Hawaii (Too et al. 2014), and the Red Sea (Yonow 2008). Here documented as a first record for Thai waters.

##### *Haloa* Pilsbry, 1921

###### * Haloa

Taxon classificationAnimaliaCephalaspideaHaminoeidae

sp.

4492F331-656F-50F4-B108-03CD1362FFED

Figure 7A

####### Material examined.

Three individuals 2–5 mm, LB.

####### Ecology.

While rarer than most other soft sediment associated Haminoeidae spp., from Koh Tao, the strong association with cyanobacterial mats is a shared feature across these species. Depth 8–18 m.

**Figure 7.**
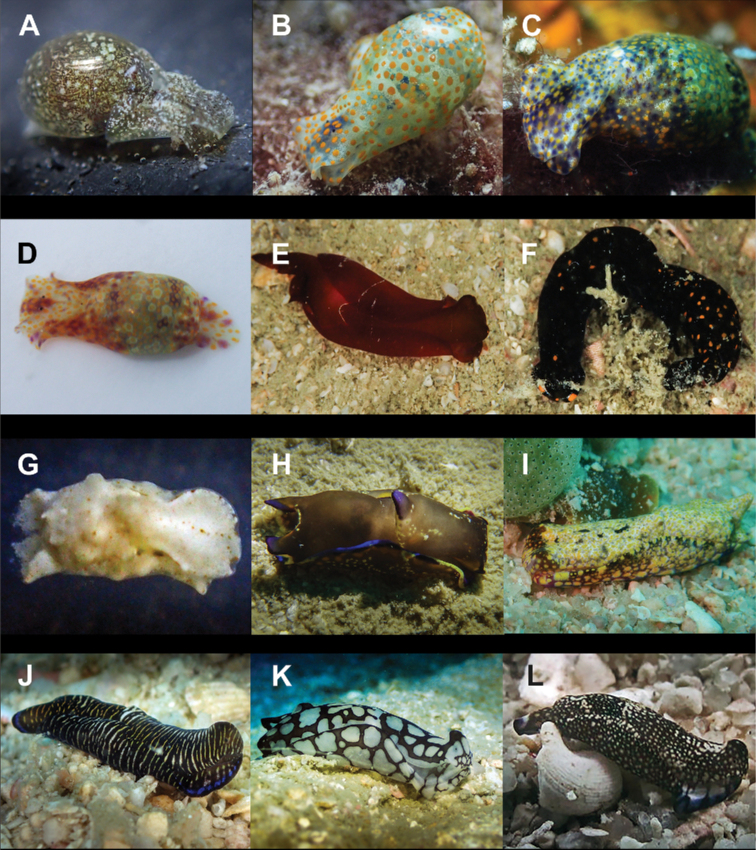
**A***Haloa* sp. 5 mm **B***Lamprohaminoea ovalis* 18 mm **C***L. ovalis* 9 mm (photograph by Elouise Haskin) **D***L. ovalis* 4 mm **E**Chelidonura cf. castanea 62 mm **F***Chelidonura punctata* 32 and 34 mm **G***Niparaya* sp. 4 mm **H***Philinopsis speciosa* 18 mm **I** ‘Philinopsis’ coronata 35 mm (photograph by Phannee Mccarthy) **J***Tubulophilinopsis lineolata* 38 mm **K***Tubulophilinopsis pilsbryi* 35 mm **L***Tubulophilinopsis reticulata* 30 mm (photograph by Kirsty Magson).

####### Distribution.

Unknown.

##### *Lamprohaminoea* Habe, 1952

###### * Lamprohaminoea ovalis

Taxon classificationAnimaliaCephalaspideaHaminoeidae

(Pease, 1868)

A7807007-6702-5D2E-AD6B-B6501A184BB4

Figure 7B, C, D

####### Material examined.

Six individuals 9–35 mm, SB; eight individuals 6–21 mm; LB.

####### Ecology.

White morphs (Fig. 7B) among rubble in shallow coral reef habitats at depths 4–8 m. Purple/red morphs in soft sediment habitats outside the coral reef where it is found strongly associated with mats of cyanobacteria on the benthos. Purple/red morphs (Fig. 7C, D) may also be found near the reef edge when cyanobacteria abundances increase, indicating a possible seasonal influence in abundance. Often found together with other Haminoeidae species from Koh Tao, apart from *Atys* sp. White and purple/red morphs not found together suggesting a fundamental division in local ecology (i.e., diet), potentially contributing to the difference in colouration. Specimens from Koh Tao have been observed to be ingested, and sometimes rejected post-ingestion, by the wrasses *Cheilinus fasciatus* (Bloch, 1791) and *Thalassoma lunare* (Linnaeus, 1758). This method of prey rejection has been suggested as a vector for prey dispersal of sea slugs onto opportunistic predatory scleractinian corals (Mehrotra et al. 2019). Depth 8–18 m.

####### Distribution.

*Lamprohaminoea ovalis* known from the Red Sea, Oman, Philippines, Vanuatu, Guam, French Polynesia, Hawaii (Oskars and Malaquias 2020), Australia (Nimbs and Smith 2016), Mariana Islands (Carlson and Hoff 2003), Japan (Hori 2017), Marshall Islands (Marcus and Burch 1965), and invasive in the Mediterranean Sea (Fernández-Vilert et al. 2018). It is here recorded for the first time from Thai waters.

####### Remarks.

With the recent comprehensive review of the genus *Lamprohaminoea* (Oskars and Malaquias 2020), specimens from Koh Tao were identified as *L. ovalis*. This identification was based on shell morphology and external colouration, the variability of both now being well documented. Despite specimens from Koh Tao being from the same species, the different morphs have been found to exhibit distinct ecological characteristics.

#### Superfamily Philinoidea Gray, 1850 (1815)

**Family Aglajidae Pilsbry, 1895 (1847)**

##### Genus *Chelidonura* A. Adams, 1850

###### * Chelidonura cf. castanea

Taxon classificationAnimaliaCephalaspideaAglajidae

Yonow, 1994

8DA68F14-9290-5428-BC36-81B87685A82A

Figure 7E

####### Material examined.

Two specimens 62–74 mm, TT.

####### Ecology.

In soft sediment habitats outside the coral reef. Depth 22–26 m.

####### Distribution.

*Chelidonura castanea* is currently known only from the Maldives (Yonow 1994). Unconfirmed sightings have also been made from Mozambique and Myanmar (iNaturalist 2011; TSS 2020). Here representing a first record for Thai waters (but see below).

####### Remarks.

Specimens from Koh Tao differ from those originally described by lacking orange spots across the dorsum, instead having only two tiny orange spots on the anterior portion of the head, on either side of the mouth. Additionally, the body is uniformly deep reddish brown with a thin white line on the upper margin of the cephalic shield. In the larger specimen (74 mm), both orange spots and the white line were markedly less distinct. The presence and absence of yellow/orange spots in such Aglajids has been shown to be an unreliable character for species delimitations (Turner and Wilson 2012). To date, *C. castanea* is only known from the Indian Ocean; however, a distribution from Thailand was recorded, without reference to any source, by Gosliner et al. (2008) but omitted in later versions (Gosliner et al. 2018). Therefore, we hereby provide details of a similar species from the Gulf of Thailand waters as a first record.

###### * Chelidonura punctata

Taxon classificationAnimaliaCephalaspideaAglajidae

Eliot, 1903

14A34B7C-2018-5E23-88C4-0B9932179A1D

Figure 7F

####### Material examined.

Four specimens 32–39 mm, TT.

####### Ecology.

In soft sediment habitats outside the coral reef. Depth 22–26 m.

####### Distribution.

*Chelidonura punctata* is currently known from Kenya (Mangubhai 2007), Mozambique (Tibiriçá and Malaquias 2016), Zanzibar, Mauritius, the Chagos Islands, the Maldives (Yonow 2012), India (Apte 2009), and Myanmar (Sanpanich and Duangdee 2019). *Chelidonura punctata* has been recorded from the Andaman sea of Thailand (Gosliner et al. 2008; Nabhitabhata 2009) and is here recorded for the first time from the Gulf of Thailand.

##### Genus *Niparaya* Zamora-Silva & Malaquias, 2018

###### * Niparaya

Taxon classificationAnimaliaCephalaspideaAglajidae

sp.

0C316ADE-22D5-5A51-953A-39FD8B611720

Figure 7G

####### Material examined.

Two specimens 4 mm, CB; one specimen 3 mm, TT.

**Ecology.** Among rubble in coral reef habitats and soft sediments near the reef edge. Depth 4–8 m.

####### Distribution.

*Niparaya* sp. 3 is currently known only from eastern Malaysia (Gosliner et al. 2018).

##### Genus *Philinopsis* Pease, 1860

###### Philinopsis speciosa

Taxon classificationAnimaliaCephalaspideaAglajidae

Pease, 1860

09089962-11E7-5F81-8BD0-B48E0581A9DE

Figure 7H

####### Material examined.

Two specimens 18–26 mm, SB; one specimen 14 mm, TT; one specimen 11 mm, SN.

####### Ecology.

In soft sediment habitats outside the coral reef. Depth 14–26 m.

####### Distribution.

Widespread across the Indo-Pacific including Mozambique (Tibiriçá and Malaquias 2016), Maldives (Yonow 1994), Vietnam (Martynov and Korshunova 2012), Australia (Nimbs and Smith 2016), Guam, Hawaii, the Galapagos Islands (Zamora-Silva and Malaquias 2018), South Africa, Tanzania, the Philippines, Indonesia, Papua New Guinea, Japan, Panama (Gosliner et al. 2008), and Red Sea (Yonow 1990). Previously documented from Thai waters (Nabhitabhata 2009) but a specific location was not given. Here confirmed from the Gulf waters of Thailand.

###### * ‘Philinopsis’ coronata

Taxon classificationAnimaliaCephalaspideaAglajidae

(Gosliner, 2011)

3B76337F-4984-5930-9642-4F536FF39738

Figure 7I

####### Material examined.

One specimen 35 mm, SRB.

####### Ecology.

In soft sediment habitats outside the coral reef. Depth 10 m.

####### Distribution.

‘*Philinopsis’ coronata* is known from the Philippines (Gosliner 2011) and Indonesia (Gosliner et al. 2018). Here recorded for the first time from Thai waters.

####### Remarks.

The taxonomic validity of this species name remains unresolved after it was designated the type species for the recently erected genus *Spinophallus* by Zamora-Silva and Malaquias (2018), which is a junior homonym of *Spinophallus* A. Riedel, 1962 [Gastropoda, Pristilomatidae] (MolluscaBase 2020). To date, no replacement name has been proposed leaving us to retain the use of the earlier but incorrect genus designation for this species.

##### Genus *Tubulophilinopsis* Zamora-Silva & Malaquias, 2018

###### Tubulophilinopsis lineolata

Taxon classificationAnimaliaCephalaspideaAglajidae

(H. Adams & A. Adams, 1854)

8C499320-956B-5E57-A309-89E37F31AB69

Figure 7J

####### Material examined.

One specimen 45 mm, LB; two specimens 32–38 mm, TT.

####### Ecology.

In soft sediment habitats outside the coral reef. Depth 12–18 m.

####### Distribution.

Currently known only from Australia (Nimbs and Smith 2016), Japan and the Philippines (Gosliner et al. 2018). Recorded from the Gulf waters of Thailand (Jensen 1998).

###### Tubulophilinopsis pilsbryi

Taxon classificationAnimaliaCephalaspideaAglajidae

(Eliot, 1900)

ACA9AEED-8766-56C5-818D-EC0524093668

Figure 7K

####### Material examined.

Two specimens 35–39 mm, SN; two specimens 25–42 mm, TT; one specimen 19 mm, MB.

####### Ecology.

Abundant in soft sediment habitats outside the coral reef. A single individual of the species was observed being ingested by the scleractinian coral *Heteropsammia cochlea* (Mehrotra et al. 2019). Depth 12–28 m.

####### Distribution.

Widespread across the Indo-Pacific including Mozambique (Tibiriçá and Malaquias 2016), Myanmar (Sanpanich and Duangdee 2019), Vietnam (Martynov and Korshunova 2012), Australia (Nimbs and Smith 2016), the Philippines, Vanuatu (Zamora-Silva and Malaquias 2018), Madagascar, Malaysia, Indonesia, Papua New Guinea, Palau, Guam, Marshall Islands, and Hawaii (Gosliner et al. 2008). Documented from the Gulf of Thailand (Mehrotra and Scott 2016).

###### * Tubulophilinopsis reticulata

Taxon classificationAnimaliaCephalaspideaAglajidae

(Eliot, 1903)

FB9E609C-1CCB-566D-AD3F-15891FC40142

Figure 7L

####### Material examined.

One specimen 30 mm, SN.

####### Ecology.

In soft sediment habitats outside the coral reef. Depth 14–16 m.

####### Distribution.

Widespread across the Indo-Pacific including Mozambique (Tibiriçá and Malaquias 2016), Vietnam (Martynov and Korshunova 2012), Taiwan (Huang et al. 2016), Australia (Nimbs and Smith 2016), Marshall Islands (Zamora-Silva and Malaquias 2018), Tanzania, South Africa, Madagascar (Gosliner et al. 2008), and the Red Sea (Yonow 1990). Here documented as a first record for Thai waters.

##### Genus *Migaya* Ortea, Caballer & Espinosa, 2014

###### * Migaya

Taxon classificationAnimaliaCephalaspideaAglajidae

sp.

FA250F23-BCE7-5E43-872E-FE0ABC3F2D1E

Figure 8A

####### Material examined.

One specimen 3 mm, LB.

####### Ecology.

In soft sediment habitats outside the coral reef. Depth 24 m.

####### Distribution.

Currently known only from the Gulf of Thailand, documented here for the first time.

####### Remarks.

Ortea Rato et al. (2014) described the genus *Migaya* to hold all the Caribbean and Indo-Pacific cephalaspideans that were found to cluster (subclade B.2.) with *Aglaja felis* Er. Marcus & Ev. Marcus, 1970 in the molecular phylogeny inferred by Camacho-García et al. (2013). These authors transferred *A. felis* to the genus *Nakamigawaia* Kuroda & Habe, 1961 based on the apparently wide distribution of *A. felis* in the Indo-Pacific, assuming that they could only belong to the Japanese genus *Nakamigawaia* because of the similarities in their external morphology and colouration, but they did not include representatives of the type species of the genus, *N. spiralis* Kuroda & Habe, 1961, in their study, nor any other co-generic species coming from Japan. Ortea Rato et al. (2014) compared the shells *N. spiralis* with those of *A. felis* in the context of a wide-range shell comparison including all the Aglajidae, and concluded that both species belonged to different genera, consequently describing the genus *Migaya*. Afterwards, Zamora-Silva and Malaquias (2018) published a new molecular phylogeny based on a wider taxonomical sampling within the Aglajidae in which they synonymised the genus *Migaya* and transferred *A. felis* to the genus *Nakamigawaia*. Again, these authors did not include representatives of *N. spiralis* from Japan, but similar species from Australia and Papua New Guinea, without checking their internal anatomies. For these reasons, given the high rate of endemicity of the Japanese sea slugs, and after the study of the shell of the specimen from Thailand (bearing a similar shell to that of *M. felis*), we prefer to maintain the genus *Migaya* until representatives of *N. spiralis* from Japan are sequenced and compared in a phylogenetic context.

**Figure 8.**
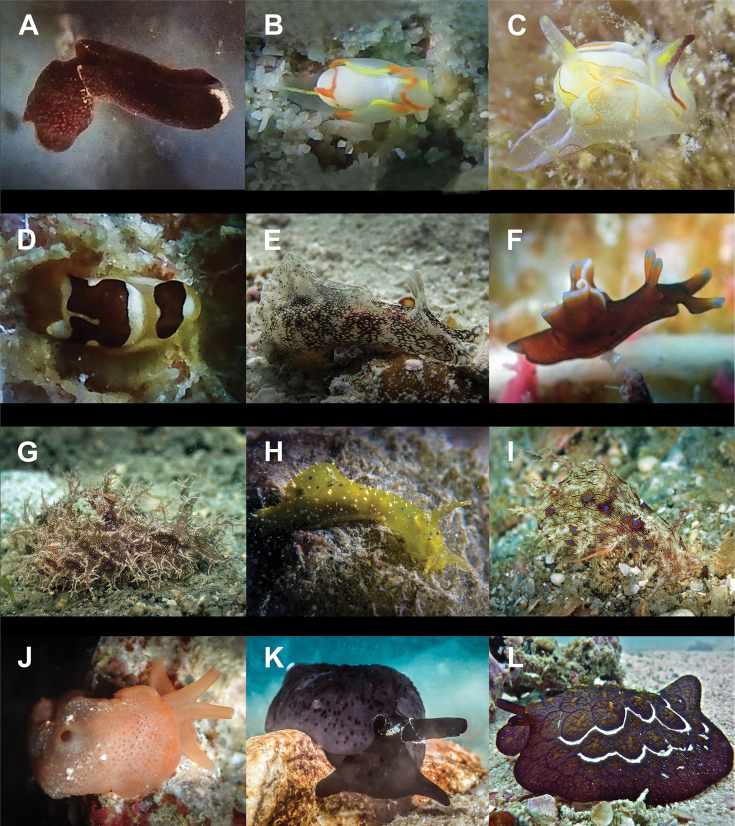
**A***Migaya* sp. 3 mm **B***Siphopteron makisig* 3 mm **C***Siphopteron* sp. 3 mm (photograph by Will Malsukum) **D***Philine orca* 3 mm **E***Aplysia kurodai* 30 mm (photograph by Geoffrey Chamayou) **F***Aplysia nigrocincta* 9 mm **G**Bursatella cf. ocelligera 65 mm (photograph by Elouise Haskin) **H***Stylocheilus longicauda* 45 mm (photograph by Kirsty Magson) **I***Stylocheilus striatus* 29 mm **J**Berthella cf. caledonica 10 mm **K***Berthella martensi* 60 mm (photograph by Paddy Steele) **L***Pleurobranchus forskalii* 130 mm (photograph by Tine Kvamme).

#### Family Gastropteridae Swainson, 1840

**Genus *Siphopteron* Gosliner, 1989**

##### * Siphopteron makisig

Taxon classificationAnimaliaCephalaspideaGastropteridae

Ong & Gosliner, 2017

992F834D-E138-585B-9EAB-B93CE7DC39C9

Figure 8B

###### Material examined.

One individual 3 mm, SO.

###### Ecology.

In soft sediment habitats outside the coral reef. Depth 12–16 m.

###### Distribution.

Currently known only from the Philippines, Indonesia, and Australia (Ong et al. 2017). Here documented as a first record for Thai waters.

##### * Siphopteron

Taxon classificationAnimaliaCephalaspideaGastropteridae

sp.

811E338E-610B-59ED-B005-05CEE8C15DB4

Figure 8C

###### Material examined.

One individual 3 mm, RR.

###### Ecology.

In soft sediment habitats outside the coral reef. Depth 20 m.

###### Distribution.

Unknown

#### Family Philinidae Gray, 1850 (1815)

**Genus *Philine* Ascanius, 1772**

##### * Philine orca

Taxon classificationAnimaliaCephalaspideaPhilinidae

(Pease, 1860)

BE2247B8-78B5-588E-AAED-94A214B6B1E9

Figure 8D

###### Material examined.

Two individuals 2–4 mm, CB; one individual 3 mm, SB.

###### Ecology.

Observed exclusively under dead Fungiidae coral skeletons where it is extremely cryptic, although it may be abundant. The only cephalaspidean species recorded exclusively from the coral reef habitat at Koh Tao. Depth 3–8 m.

###### Distribution.

Widespread across the Indo-Pacific including Japan (Baba 1990), Australia (Nimbs and Smith 2016), Madagascar, Malaysia, the Philippines, Indonesia, Papua New Guinea, Hawaii, and the Galapagos Islands (Gosliner et al. 2008). Here documented as a first record for Thai waters.

#### Order Aplysiida

**Superfamily Aplysioidea Lamarck, 1809**

**Family Aplysiidae Lamarck, 1809**

##### Genus *Aplysia* Linnaeus, 1767

###### Aplysia kurodai

Taxon classificationAnimaliaAplysiidaAplysiidae

Baba, 1937

5D26A752-5D4F-5561-94C1-75558CC4C43F

Figure 8E

####### Material examined.

Two specimens 60–81 mm, SN; one specimen 30 mm, MH.

####### Ecology.

In soft sediment habitats, occasionally found in aggregations, although more often observed as solitary. Depth 8–22 m.

####### Distribution.

Currently known only from China (Guang-Yu and Tchang 1965), Korea (Lee et al. 2014), and Japan (Baba 1937). Documented from the Gulf of Thailand (Mehrotra and Scott 2016).

###### * Aplysia nigrocincta

Taxon classificationAnimaliaAplysiidaAplysiidae

von Martens, 1880

A689B93C-5DE1-5E34-B981-3235DBC95917

Figure 8F

####### Material examined.

One specimen 9 mm, SB.

####### Ecology.

Under a dead Fungiidae coral among rubble in shallow coral reef habitats. Depth 6 m.

####### Distribution.

Across the Indo-Pacific including Mozambique, Mauritius, the Philippines, Indonesia, Papua New Guinea, Vanuatu (Golestani et al. 2019), Maldives (Yonow 1994 as *Aplysia fasciata*), and the Red Sea (Yonow 1990 as Aplysia cf. parvula.). Here documented as a first record for Thai waters.

##### Genus *Bursatella* Blainville, 1817

###### * Bursatella cf. ocelligera

Taxon classificationAnimaliaAplysiidaAplysiidae

(Bergh, 1902)

55ABA1A3-2CFA-5E5E-85A9-D6D7C1E91A9C

Figure 8G

####### Material examined.

One specimen 65 mm, SB.

####### Ecology.

In soft sediment habitats where it grazes on cyanobacterial mats on the benthos. Depth 18–25 m.

####### Distribution.

*Bursatella ocelligera* is known only from the Philippines (Bazzicalupo et al. 2020) and the Gulf of Thailand (Bergh 1902). *Bursatella leachii* is circumtropical (Bazzicalupo et al. 2020) including Brazil (Galvão Filho et al. 2015), Guadeloupe (Ortea Rato et al. 2012), Spain (González-Wangüemert et al. 2014), Italy (Travaglini and Crocetta 2019), Tunisia (Zakhama-Sraieb 2009), Morocco (Selfati et al. 2017), Ghana (Bebbington 1969), Iran (Rezai et al. 2016), India (Sethi et al. 2015), Vietnam (Martynov and Korshunova 2012), Australia (Nimbs and Smith 2016), New Zealand (Appleton et al. 2002), South Africa, Madagascar, and Hawaii (Gosliner et al. 2008).

####### Remarks.

Eales and Engel (1935) synonymised all species of *Bursatella* into the single circumtropical species *B. leachii*. Recent evidence (Bazzicalupo et al. 2020) has supported the presence of a second species, *B. ocelligera*, based on internal morphological differences such as an unarmed penis and supported by molecular data. This name was attributed to specimens described from the Gulf of Thailand by Bergh (1902), who was the first to describe specimens with an unarmed penis. At present, there is no reliable way of discerning between the two species based on external morphology alone, and no available molecular data for specimens from the Gulf of Thailand. The internal anatomy of specimens from Koh Tao could not be investigated as part of the present study; however, the proximity to the type locality (Koh Chang) supports the need for comprehensive analyses of specimens from the Gulf of Thailand in particular, given the wide geographical range of *B. leachii* and the recent separation of other ‘circumtropical’ aplysiids.

##### Genus *Stylocheilus* Gould, 1852

###### Stylocheilus longicauda

Taxon classificationAnimaliaAplysiidaAplysiidae

(Quoy & Gaimard, 1825)

7C6286D4-29EB-52F6-9D8D-E1624CD81F39

Figure 8H

####### Material examined.

One specimen 45 mm, SRB; one specimen 30 mm, TT.

####### Ecology.

In soft sediment habitats rarely and upon mooring ropes where it grazes on cyanobacteria. Far less common than *S. striatus.* The association with mooring ropes is believed to be driven by its pelagic lifestyle, as these ropes act as mid-water substrates for cyanobacterial growth. Depth 5–18 m.

####### Distribution.

Circumtropical including Brazil (Galvão Filho et al. 2015), Gulf of Oman (Fatemi and Attaran 2015), Red Sea (Yonow 2008), India (Chinnadurai et al. 2014), Australia (Nimbs and Smith 2016), Tanzania, the Philippines, and Hawaii (Gosliner et al. 2008). Documented from the Gulf of Thailand (Mehrotra and Scott 2016).

####### Remarks.

Recent work (Yonow 2012; Bazzicalupo et al. 2020), has indicated the need for morphological and molecular examination of *S. longicauda* across their range with the taxonomic validity of the species being questioned. In the present work, *S. longicauda* is treated as distinct from *S. striatus* based on differences in external morphology (consistently shorter papillae and yellow/lime-green colouration in *S. longicauda*) and ecology. We here retain the use of the compound noun used in the original description of the species (see Nimbs et al. 2017).

###### Stylocheilus striatus

Taxon classificationAnimaliaAplysiidaAplysiidae

(Quoy & Gaimard, 1832)

FFD12CE9-D03A-5847-9170-256D568E60EB

Figure 8I

####### Material examined.

One specimen 25 mm, SRB; one specimen 22 mm, CB; one specimen 29 mm, TT.

####### Ecology.

From shallow and deep soft sediment habitats grazing of mats of cyanobacteria on the benthos. Depth 1–18 m.

####### Distribution.

Circumtropical including Brazil (Galvão Filho et al. 2015), Mexico (Ortigosa et al. 2015), Guadeloupe (Ortea Rato et al. 2012), the Azores (Malaquias et al. 2009), Mozambique (Jochum and Favre 2017), India (Apte 2009), Vietnam (Martynov and Korshunova 2012), Indonesia (Eisenbarth et al. 2018), Australia (Nimbs and Smith 2016), French Polynesia (Horwitz et al. 2017), Fiji (Thaman et al. 2017), the Caribbean, Galapagos, South Africa, Red Sea, Hawaii and California (Gosliner et al. 2008). Documented from the Gulf of Thailand (Mehrotra and Scott 2016), and from Andaman Sea (as *S. longicauda*) by Jensen (1998).

#### Clade Nudipleura Wägele & Willan, 2000

**Order Pleurobranchida Deshayes, 1832**

**Superfamily Pleurobranchoidea Gray, 1827**

**Family Pleurobranchidae Gray, 1827**

##### Genus *Berthella* Blainville, 1824

###### * Berthella cf. caledonica

Taxon classificationAnimaliaPleurobranchidaPleurobranchidae

(Risbec, 1928)

E00BEE42-6E98-5AC4-BBEF-C83E1EF427A7

Figure 8J

####### Material examined.

Three specimens 10–15 mm, TW.

####### Ecology.

Under coral rubble in shallow coral reef habitats. Depth 6–8 m.

####### Distribution.

*Berthella caledonica* is known from New Caledonia (Risbec 1928), Mariana Islands (Carlson and Hoff 2003), Hawaii (Johnson 2002a), Marshall Islands (Johnson 2002b), Australia (Cobb 2009), and Japan (Bolland 2002).

####### Remarks.

Overall colour variable from pink to light or dark brown, with numerous small, low tubercles across the dorsal surface, often surrounded by a brown ring, and with dark brown apices. A prominent brown mark surrounded by a diffuse ring of translucent white is located centrally on the dorsal surface. While specimens from Koh Tao resemble the description of *Berthella caledonica* (Risbec, 1928) rather well, records of *Berthella africana* (Pruvot-Fol, 1956) have also been made from Thailand, with an unclear locality (Nabhitabhata 2009). Both species share external similarities, in particular the presence of a brown spot or ‘hole’ found centrally on the dorsum, and the need for clarification between both species has been noted (Gosliner et al. 2008; Rudman 2009b). The present species is most similar to *Berthella* sp. 1 of Gosliner et al. (2018). With the Gulf of Thailand being distant from the type localities of both species (Morocco for *B. africana* and New Caledonia for *B. caledonica*), and the Pacific range currently known for *B. caledonica*, the present species is treated as potentially distinct until such a time as closer examinations can be made.

###### * Berthella martensi

Taxon classificationAnimaliaPleurobranchidaPleurobranchidae

(Pilsbry, 1896)

4B08B9EA-987A-5975-92C5-79FA9B564A8B

Figure 8K

####### Material examined.

One specimen 60 mm, LB.

####### Ecology.

Exclusively recorded from soft sediment habitats outside coral reefs. Depth 11–21 m.

####### Distribution.

Widespread throughout the Indo-Pacific including the Red Sea (Yonow 2015), Mozambique (Tibiriçá et al. 2017), India (Sreeraj et al. 2012), Maldives (Yonow 1994), Tanzania, Mauritius, Indonesia, the Philippines, Taiwan, Australia, Papua New Guinea, Solomon Islands, Hawaii, and the Pacific coast of Mexico (Gosliner et al. 2008).

####### Remarks.

Individuals from Koh Tao have a dark, almost black mantle with numerous inconspicuous black spots. *Berthella martensi* was recorded by Nabhitabhata (2009) based on a local record (in Thai), but the location(s) of this record is unknown. Therefore, while *B. martensi* is known from the Gulf of Thailand, its presence along the Andaman coast of Thailand is unconfirmed.

##### Genus *Pleurobranchus* Cuvier, 1804

###### Pleurobranchus forskalii

Taxon classificationAnimaliaPleurobranchidaPleurobranchidae

Rüppell & Leuckart, 1828

1830410A-0149-5726-A4BB-34EAD1B7FA6E

Figure 8L

####### Material examined.

One specimen 265 mm, SN; one specimen 55 mm, TT.

####### Ecology.

Exclusively recorded from soft sediment habitats outside coral reefs. Observed feeding on colonies of the tunicate *Didemnum molle* Herdmann, 1886. Depth 11–21 m.

####### Distribution.

Widespread throughout the Indo-Pacific including Mozambique (Tibiriçá et al. 2017), Gulf of Oman (Fatemi and Attaran 2015), India (Apte and Bhave 2014), Tanzania, the Red Sea, the Philippines, Indonesia, Japan, Australia, Papua New Guinea, and Fiji (Gosliner et al. 2008). First documented from the Gulf of Thailand by Mehrotra and Scott (2016).

#### Order Nudibranchia Cuvier, 1817

**Suborder Doridina Odhner, 1934**

**Superfamily Doridoidea Rafinesque, 1815**

**Family Actinocyclidae O’Donoghue, 1929**

##### Genus *Hallaxa* Eliot, 1909

###### * Hallaxa iju

Taxon classificationAnimaliaNudibranchiaActinocyclidae

Gosliner & Johnson, 1994

D30E6DBD-C4B7-58DD-83BD-DFAA4EDFC71B

Figure 9A

####### Material examined.

One specimen 10 mm, SO.

####### Ecology.

Among rubble in coral reef and reef edge habitats. Associated with an unidentified pale/creamy white sponge. Depth 4–12 m.

####### Distribution.

Across the Pacific including Australia (Nimbs and Smith 2016), the Philippines, Papua New Guinea, Japan, the Marshall Islands (Gosliner and Johnson 1994), Hong Kong, and Hawaii (Gosliner et al. 2008). Here representing a first record for Thai waters.

**Figure 9.**
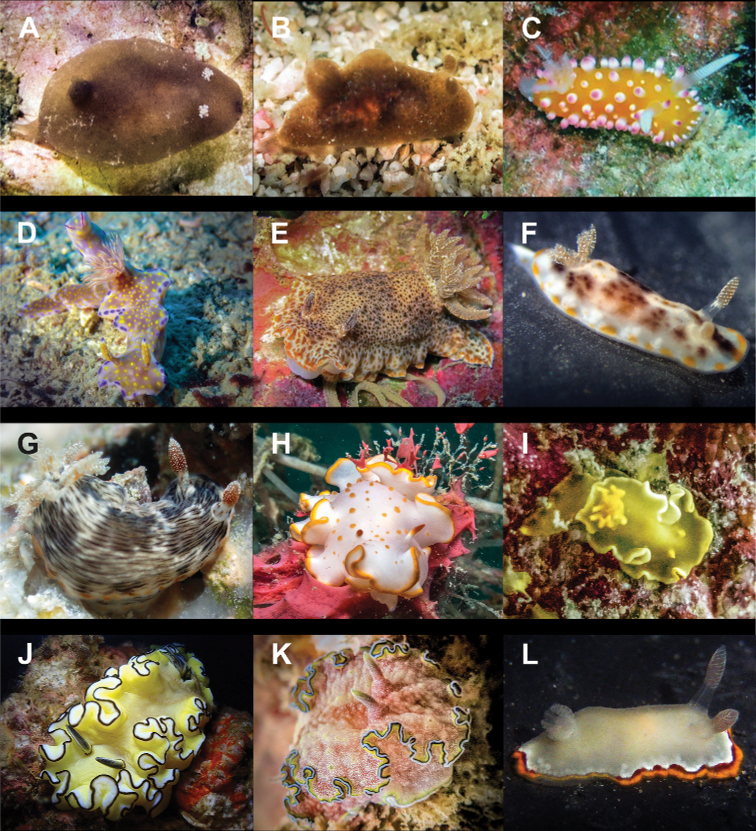
**A***Hallaxa iju* 10 mm **B***Hallaxa indecora* 8 mm **C***Cadlinella ornatissima* 30 mm (photograph by Mati Pauner) **D***Ceratosoma tenue* 85 mm **E***Chromodoris mandapamensis* 50 mm (photograph by Tine Kvamme) **F**Chromodoris cf. mandapamensis 7 mm **G**Chromodoris cf. balat 25 mm (photograph by Elouise Haskin) **H***Diversidoris aurantionodulosa* 30 mm **I***Diversidoris crocea* 8 mm (photograph by Pau Urgell Plaza) **J***Doriprismatica atromarginata* 60 mm **K**Glossodoris cf. cincta 40 mm **L**Goniobranchus cf. albonares 5 mm.

###### * Hallaxa indecora

Taxon classificationAnimaliaNudibranchiaActinocyclidae

(Bergh, 1905)

A029791A-73BE-566B-A598-B0132638DAD3

Figure 9B

####### Material examined.

One specimen 8 mm, SRB; one specimen 8 mm, CB.

####### Ecology.

Among rubble and under dead fungiid coral skeletons in coral reef habitats. Occasionally near isolated unattached colonies of sponge in deeper soft sediment habitats. Associated with an unidentified pale/creamy white sponge. Depth 4–20 m.

####### Distribution.

Red Sea (Yonow 2008) and across the Indo-Pacific including Australia (Nimbs and Smith 2016), the Gulf Aden, the Philippines, Indonesia, Japan, New Caledonia, and American Samoa (Gosliner et al. 2008). Here representing a first record for Thai waters.

#### Family Chromodorididae Bergh, 1891

**Genus *Cadlinella* Thiele, 1931**

##### Cadlinella ornatissima

Taxon classificationAnimaliaNudibranchiaChromodorididae

(Risbec, 1928)

26933876-97B2-5969-A81E-4B0F65587A01

Figure 9C

###### Material examined.

Two specimens 12–30 mm, TT.

###### Ecology.

On rocks and among corals at offshore pinnacles. Depth 8–25 m.

###### Distribution.

Widespread in the Indo-Pacific including New Caledonia (Risbec 1928), Japan (Baba 1949), Mozambique (Tibiriçá et al. 2017), Chagos Islands (Yonow et al. 2002), India (Ramakrishna et al. 2010), Sri Lanka, Gulf of Oman, Réunion Island (Yonow 2012), Red Sea (Yonow 2008), Myanmar (Sanpanich and Duangdee 2019), Australia (Nimbs and Smith 2016), Taiwan, Hong Kong (Gosliner et al. 2008), Singapore (Lim and Chou 1970), the Gulf of Thailand, and the Andaman coast of Thailand (Chavanich et al. 2013).

##### Genus *Ceratosoma* A. Adams & Reeve, 1850

##### * Ceratosoma tenue

Taxon classificationAnimaliaNudibranchiaChromodorididae

Abraham, 1876

237F78C0-A302-5732-9656-43D5D619CF6C

Figure 9D

###### Material examined.

One specimen 85 mm, AM.

###### Ecology.

Soft sediment habitat. Depth 26 m.

###### Distribution.

Widespread throughout the Indo-Pacific including Japan (Baba 1949), Indonesia (Yonow 2001), South Africa, Mozambique, Malaysia, Australia, New Caledonia, Hawaii (Gosliner et al. 2008), and the Red Sea (Yonow 2008). Here representing a first record for Thai waters.

##### Genus *Chromodoris* Alder & Hancock, 1855

##### Chromodoris mandapamensis

Taxon classificationAnimaliaNudibranchiaChromodorididae

Valdés, Mollo & Ortea, 1999

A1963B5A-0DD0-549B-AE85-9DFCBBADCD7C

Figure 9E

###### Material examined.

One specimen 50 mm, CP.

###### Ecology.

Among corals and coral rubble at offshore pinnacle sites. 9–20 m.

###### Distribution.

Widespread throughout the Indo-Pacific including Mozambique (Tibiriçá et al. 2017), India (Valdés et al. 1999), South Africa, Solomon Islands, Papua New Guinea, Myanmar (Gosliner et al. 2008), and Gulf of Thailand (Chavanich et al. 2013).

###### Remarks.

While externally matching the original description of the species completely, the internal anatomy was not analysed to verify this, the importance of which is particular to this and other similar species (Layton et al. 2018). See ‘Remarks’ for Chromodoris cf. mandapamensis below.

##### * Chromodoris cf. mandapamensis

Taxon classificationAnimaliaNudibranchiaChromodorididae

Valdés, Mollo & Ortea, 1999

A3EDECF2-D08B-5525-AC1D-CD9D53EF9DD8

Figure 9F

###### Material examined.

Three specimens 5–10 mm, CB.

###### Ecology.

Among reef rubble, in particular under dead fungiid coral skeletons, in shallow coral reef areas. Depth 2–8 m.

###### Distribution.

*Goniobranchus pruna* (Gosliner, 1994) is known from Madagascar and South Africa (Gosliner 1994) and Mozambique (Tibiriçá et al. 2017). *Chromodoris* sp. 15 is known from Philippines and New Caledonia (Gosliner et al. 2018).

###### Remarks.

Very similar to Chromodoris aff. mandapamensis (Layton et al. 2018; Bonomo and Gosliner 2020), *Chromodoris* sp. 15 (Gosliner et al. 2018), and *Goniobranchus pruna* (Gosliner 1994). While *C. mandapamensis* has been suggested as a possible synonym of *G. pruna* as discussed in Tibiriçá et al. (2017), the present species is kept separate from *C. mandapamensis* due to difference in the local ecology of both species. *Chromodoris mandapamensis* is locally recorded only from sparse observations at offshore pinnacles, with no confirmed records for the past five years, whereas C. cf. mandapamensis is regularly found in surveys in shallow reef areas near the shore. While it is possible that this species undergoes its juvenile stages closer to shore before moving out towards the offshore pinnacles, no observations have been made of this species in the intervening deeper soft sediment habitats between the two. Additionally, no individuals larger than approximately 15 mm have been recorded and no individuals that externally match *C. mandapamensis* have yet been recorded near the island.

##### * Chromodoris cf. balat

Taxon classificationAnimaliaNudibranchiaChromodorididae

Bonomo & Gosliner, 2020

E8986636-3B05-54DC-97CB-D374513EB9BF

Figure 9G

###### Material examined.

One specimen 25 mm, CB.

###### Ecology.

Among reef rubble, in particular under dead fungiid coral skeletons, in shallow coral reef areas. Depth 4–8 m

###### Distribution.

*Chromodoris balat* is known only from the Philippines (Bonomo and Gosliner 2020) and a similar species is recorded here for the first time from Thai waters.

###### Remarks.

Similar to *Chromodoris balat* in having a striated dorsum with numerous large blotches and a broken yellow-orange marginal line. This species was differentiated from the similar *Chromodoris striatella* Bergh, 1877 based on these and other features (Layton et al. 2018; Bonomo and Gosliner 2020). Our specimen is differentiated from *C. balat* by lacking small yellow, orange, and red spots on the white parts of the dorsal surface, the pale tan-coloured gills with orange spots instead of red-brown as seen in *C. balat*, and by the blotches being pale grey-brown and indistinct instead of dark and pronounced. The indistinct blotches on the dorsum, the broken yellow-orange marginal band, and the colouration of rhinophores and gills do share a resemblance with *C. mandapamensis* and C. cf. mandapamensis (the latter of which may be found living alongside C. cf. balat at Koh Tao). Given the difficulties of relying on external features for species delineation in many of these striped and spotted species of *Chromodoris* (Layton et al. 2018; Bonomo and Gosliner 2020) we refrain from committing to a species identification until specimens from Koh Tao can be investigated further. Chavanich et al. (2013) recorded *C. striatella* from both Gulf and Andaman coasts of Thailand; however, in the absence of specimen details from both areas, this distribution record may be called into question in light of the recent findings regarding the complex surrounding *C. striatella*. Further documentation of *Chromodoris* species from both coasts may clarify this.

##### Genus *Diversidoris* Rudman, 1987

##### * Diversidoris aurantionodulosa

Taxon classificationAnimaliaNudibranchiaChromodorididae

Rudman, 1987

F6891A72-7CD4-5359-8F3D-1058521946FB

Figure 9H

###### Material examined.

One specimen 30 mm, SI.

###### Ecology.

Found upon its pink host sponge, *Darwinella* sp., at deeper reef and pinnacle sites, and in muck habitats. Depth 12–30 m.

###### Distribution.

Red Sea (Yonow 2015), South Africa, Tanzania, Australia, and Hong Kong (Gosliner et al. 2008). Here representing a first record for Thai waters

###### Remarks.

This species was mistakenly identified as *Ardeadoris averni* (Rudman, 1985) by Mehrotra and Scott (2016) based on limited photographic data. Specimens found several years after initial second-hand observations have permitted reidentification, leaving *A. averni* remaining currently unrecorded in Thai waters.

##### Diversidoris crocea

Taxon classificationAnimaliaNudibranchiaChromodorididae

(Rudman, 1986)

3560B722-1B6A-5C6D-847F-491555560D10

Figure 9I

###### Material examined.

One specimen 8 mm, TT.

**Ecology.** Coral reef habitats. Usually cryptic on its sponge, a yellow *Darwinella* sp. (Rudman 2005a).

**Distribution.** Widespread throughout the Indo-Pacific including Mozambique (Tibiriçá et al. 2017), Philippines, Indonesia, Japan, Papua New Guinea (Gosliner et al. 2008), Guam (Carlson and Hoff 2003), and Australia (Rudman 1986). First documented from the Gulf of Thailand by Mehrotra and Scott (2016).

##### Genus *Doriprismatica* d’Orbigny, 1839

##### Doriprismatica atromarginata

Taxon classificationAnimaliaNudibranchiaChromodorididae

(Cuvier, 1804)

AAD58787-ECBF-5579-AB40-7405C74400B5

Figure 9J

###### Material examined.

One specimen 60 mm, CP.

###### Ecology.

On rocks and among corals at offshore pinnacles. Depth 8–25 m.

###### Distribution.

Widespread throughout the Indo-Pacific including South Africa, Red Sea, French Polynesia, Solomon Islands, China, Philippines (Gosliner 1987), Papua New Guinea, Australia (Rudman 1986), Myanmar (Sanpanich and Duangdee 2019), Japan (Baba 1949), Indonesia (Yonow 2001), Gulf of Thailand (Jensen 1998), Mauritius (Yonow and Hayward 1991), and the Andaman coast of Thailand (Chavanich et al. 2013).

##### Genus *Glossodoris* Ehrenberg, 1831

##### Glossodoris cf. cincta

Taxon classificationAnimaliaNudibranchiaChromodorididae

(Bergh, 1888)

998A031E-1648-595B-A8A0-50D1E053F21C

Figure 9K

###### Material examined.

One specimen 40 mm, HWB.

###### Ecology.

Coral reefs throughout the island. Depth 5–15 m.

###### Distribution.

Papua New Guinea, the Philippines and Madagascar (Matsuda and Gosliner 2018). First documented from the Gulf of Thailand by Mehrotra and Scott (2016).

###### Remarks.

Previously recorded as *Glossodoris cincta* (Bergh 1888), recent work by Matsuda and Gosliner (2018) has shown that Southeast Asian/western Pacific species may be distinct. However, this cannot be verified until specimens of *G. cincta* are analysed from the type locality of Mauritius. Therefore, the present species, which externally matches the description by Matsuda and Gosliner, is separated from the true Indian Ocean *Glossodoris cincta* for now.

##### Genus *Goniobranchus* Pease, 1866

##### * Goniobranchus cf. albonares

Taxon classificationAnimaliaNudibranchiaChromodorididae

(Rudman, 1990)

3391151A-61B4-5941-BC57-E6827BA82841

Figure 9L

###### Material examined.

Three specimens 4–6 mm, CB.

###### Ecology.

Observed under dead fungiid coral skeletons and occasionally among rubble in shallow coral reef. Depth 3–8 m

###### Distribution.

*Goniobranchus albonares* is known from Australia (Rudman 1990), Japan (Gosliner et al. 2008), Madagascar (Rassat 2016), and Mozambique (Tibiriçá et al. 2017).

###### Remarks.

Externally resembling both *Goniobranchus albonares* (Rudman, 1990) and *Goniobranchus rubrocornutus* (Rudman, 1985), the present species differs from the former by possessing a broken submarginal band of deep red and from the latter by the presence of completely white rhinophore clubs and gills as opposed to red. There is significant overlap in the range of both species, with *G. rubrocornutus* known from Australia, Hong Kong, and Japan (Rudman 1985). A comprehensive comparison of the three species is needed.

##### Goniobranchus aureopurpureus

Taxon classificationAnimaliaNudibranchiaChromodorididae

(Collingwood, 1881)

BBAB1F18-EE36-560D-BD5A-1D34CF43EE2C

Figure 10A

###### Material examined.

One specimen 45 mm, SN.

###### Ecology.

Locally rare, known only from soft sediment habitats outside the coral reef. Depth 12–16 m.

###### Distribution.

Across the Indo-Pacific including Myanmar (Sanpanich and Duangdee 2019), Australia (Nimbs and Smith 2016), the Philippines, Indonesia, China, Japan, Papua New Guinea, and New Caledonia (Gosliner et al. 2008). Known from both Andaman and Gulf waters of Thailand (Chavanich et al. 2013).

**Figure 10.**
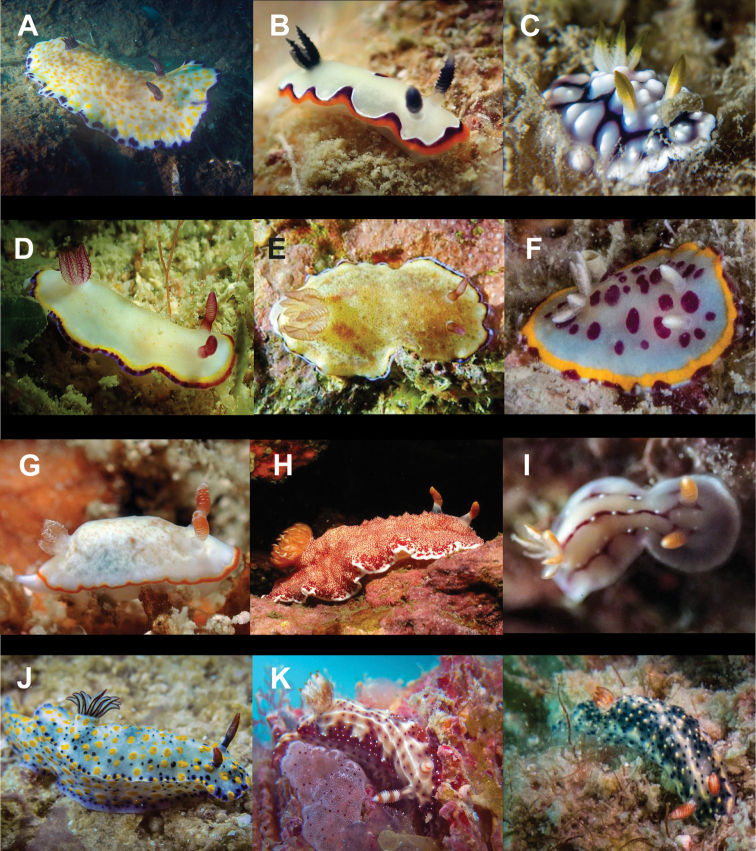
**A***Goniobranchus aureopurpureus* 45 mm **B***Goniobranchus fidelis* 12 mm **C***Goniobranchus geometricus* 15 mm (photograph by Kirsty Magson) **D, E***Goniobranchus sinensis* different morphs, 34 mm (**D**) and 55 mm (**E**) **F***Goniobranchus tumuliferus* 10 mm **G***Goniobranchus verrieri* 10 mm (photograph by Khumron Waipaka) **H***Goniobranchus* sp. 55 mm (photograph by Phannee Mccarthy) **I***Hypselodoris cerisae* 4 mm **J***Hypselodoris confetti* 24 mm (photograph by Pau Urgell Plaza) **K***Hypselodoris decorata* 18 mm **L***Hypselodoris infucata* 12 mm.

##### Goniobranchus fidelis

Taxon classificationAnimaliaNudibranchiaChromodorididae

(Kelaart, 1858)

FD79951A-9574-5C04-AABE-F332C056E752

Figure 10B

###### Material examined.

Two specimens 6–18 mm CB; one specimen 15 mm, SB; one specimen 12 mm, SD.

###### Ecology.

Coral reef habitats throughout the region. Depth 2–25 m.

###### Distribution.

Widespread throughout the Indo-Pacific including Mozambique (Tibiriçá et al. 2017), Maldives (Yonow 1994), India (Ramakrishna et al. 2010), Myanmar (Sanpanich and Duangdee 2019), Madagascar, Red Sea, Philippines, Japan, Australia, New Caledonia (Gosliner et al. 2008), and Indonesia (Yonow 2001). Known from both Andaman and Gulf waters of Thailand (Chavanich et al. 2013).

##### Goniobranchus geometricus

Taxon classificationAnimaliaNudibranchiaChromodorididae

(Risbec, 1928)

4D59662C-65AA-5D2C-9853-28F6CF21B901

Figure 10C

###### Material examined.

One specimen 15 mm, KKR.

###### Ecology.

Locally rare, known only from the reef edge and soft sediment habitats outside the coral reef. Depth 12–25 m.

###### Distribution.

Widespread throughout the Indo-Pacific including Myanmar (Sanpanich and Duangdee 2019), Tanzania, Madagascar, Guam, Japan, Papua New Guinea (Gosliner et al. 2008), Maldives (Yonow 1994), Indonesia (Yonow 2001), Philippines (Debelius 1996), and Australia (Nimbs and Smith 2016). Known from both Andaman and Gulf waters of Thailand (Chavanich et al. 2013).

##### Goniobranchus preciosus

Taxon classificationAnimaliaNudibranchiaChromodorididae

(Kelaart, 1858)

F32DDDD3-36D7-5F4B-9A15-94379152697A

###### Material examined.

None found presently.

###### Ecology.

Soft sediment habitats outside the coral reef. Depth 18–22 m.

###### Distribution.

Across the Indo-Pacific including Indonesia (Scott 2005), Malaysia, the Philippines, Papua New Guinea, Australia, New Caledonia. Recorded from Andaman and Gulf waters of Thailand (Chavanich et al. 2013).

###### Remarks.

The species has historically been recorded from Koh Tao (Mehrotra and Scott 2016) based on a citizen science effort, but no material has been found in surveys to date.

##### * Goniobranchus sinensis

Taxon classificationAnimaliaNudibranchiaChromodorididae

(Rudman, 1985)

B1F7785E-CE6B-5754-8D4A-C7710BED561C

Figure 10D, E

###### Material examined.

Three specimens 20–55 mm, CP.

###### Ecology.

Among rocks and corals at offshore rocky pinnacles. Depth 9–22 m.

###### Distribution.

Across the Indo-Pacific including the Gulf of Oman (Fatemi and Attaran 2015), India (Sreeraj et al. 2012), Malaysia, Indonesia, China, and Japan (Gosliner et al. 2008). Recorded from the Gulf waters of Thailand (Chavanich et al. 2013).

###### Remarks.

Incorrectly identified as *Goniobranchus trimarginatus* (Winckworth, 1946) by Mehrotra and Scott (2016). Subsequent observations indicate that specimens from Koh Tao are *Goniobranchus sinensis*, with individuals being recorded with marginal and submarginal bands ranging from complete to broken.

##### Goniobranchus tumuliferus

Taxon classificationAnimaliaNudibranchiaChromodorididae

(Collingwood, 1881)

937B8089-A9CA-554C-B8F0-6404E4A6C6B1

Figure 10F

###### Material examined.

Two specimens 10–15 mm, CP.

###### Ecology.

Predominantly found among coral and rock at an offshore submerged pinnacle site. Also sparsely recorded from the deeper soft sediment habitats near the island. Depth 11–25 m.

###### Distribution.

Across the western Pacific including Vietnam (Martynov and Korshunova 2012), the Philippines, Japan, Australia, and New Caledonia (Gosliner et al. 2008). Known from the Gulf of Thailand (Jensen 1998; Chavanich et al. 2013).

##### * Goniobranchus verrieri

Taxon classificationAnimaliaNudibranchiaChromodorididae

(Crosse, 1875)

75451CEF-E5F9-5AAE-AF6F-7CFD812A2B32

Figure 10G

###### Material examined.

One specimen 10 mm, GR.

###### Ecology.

Coral reefs. Depth 5–10 m.

###### Distribution.

Widespread throughout the Indo-Pacific including Mozambique (Tibiriçá et al. 2017), Tanzania, New Caledonia (Rudman 1985), Australia (Nimbs and Smith 2016), South Africa, Madagascar, Indonesia, Philippines, Hawaii (Gosliner et al. 2008), and Red Sea (Yonow 1989). Here representing a first record for Thai waters.

###### Remarks.

Locally known only from a single individual.

##### Goniobranchus

Taxon classificationAnimaliaNudibranchiaChromodorididae

sp.

A4ED04F9-2B6B-54A2-9517-638C2B2225E6

Figure 10H

###### Material examined.

One specimen 55 mm, CP.

###### Ecology.

Among rocks and corals at offshore rocky pinnacles. Depth 18 m.

###### Distribution.

*Goniobranchus* sp. 5 (Soong et al. 2020) is known from the Philippines, Indonesia, Japan, and Australia. A similar species is known from the Gulf of Thailand, incorrectly recorded as *Goniobranchus reticulatus* by Mehrotra and Scott (2016).

###### Remarks.

Red reticulated specimens of *Goniobranchus* have been known to represent a complex of species often attributed to *G. reticulatus* or *G. tinctorius*. Recent molecular work by Soong et al. (2020) concluded that none of the five distinct lineages identified corresponded exactly with the description of either of the aforementioned species. Due to the overlapping ranges and external variability of some of these, a further morphological investigation into these lineages is needed to define the species.

##### Genus *Hypselodoris* Stimpson, 1855

##### * Hypselodoris cerisae

Taxon classificationAnimaliaNudibranchiaChromodorididae

Gosliner & Johnson, 2018

2F7B532F-438B-53FB-8615-F54C3CA138A9

Figure 10I

###### Material examined.

One specimen 4 mm, CB.

###### Ecology.

Among reef rubble in shallow coral reef. Depth 4 m.

###### Distribution.

Japan, Malaysia, and Taiwan (Epstein et al. 2018). Here representing a first record for Thai waters.

###### Remarks.

Though a small individual, it is identified as *H. cerisae* based on pink and purple pigmentation and dark brown lines with white spots. An absence of orange/burnt orange pigmentation separates it from *H. krakatoa* Gosliner & Johnson, 1999.

##### * Hypselodoris confetti

Taxon classificationAnimaliaNudibranchiaChromodorididae

Gosliner & Johnson, 2018

BF1663C2-AF5E-537D-A77D-87A024B822D3

Figure 10J

###### Material examined.

Two specimens 12–24 mm, SB.

###### Ecology.

Locally found exclusively from deeper soft sediment habitats of the island. Depth 14–25 m.

###### Distribution.

Philippines, Papua New Guinea, probably Indonesia and Hong Kong (Epstein et al. 2018). Here representing a first record for Thai waters.

##### * Hypselodoris decorata

Taxon classificationAnimaliaNudibranchiaChromodorididae

(Risbec, 1928)

E8149551-99C4-55ED-BDB6-348844ECEDE5

Figure 10K

###### Material examined.

Two specimens 8–18 mm, CB.

###### Ecology.

Abundant among reef rubble, in particular under dead fungiid coral skeletons, in shallow coral reef areas. Rare in other habitats. Depth 2–12 m.

###### Distribution.

Widespread throughout the Indo-Pacific including Malaysia, Philippines, Indonesia, Papua New Guinea, New Caledonia, Vanuatu, and the Marshall Islands (Epstein et al. 2018). Here representing a first record for the Gulf of Thailand. *Hypselodoris decorata* was recorded as *Hypselodoris maculosa* (Pease, 1871) from the Andaman coast by Chavanich et al. (2013).

##### Hypselodoris infucata

Taxon classificationAnimaliaNudibranchiaChromodorididae

(Rüppell & Leuckart, 1830)

DBCB664E-C116-52F3-8753-B6138E597061

Figure 10L

###### Material examined.

One specimen 25 mm, SRB; one specimen 12 mm, CB; one specimen 8 mm, SB.

###### Ecology.

Juveniles and smaller individuals common under dead fungiid corals and reef rubble in shallow coral reef areas, making up some of the most abundant nudibranch taxa in some areas. Larger individuals rarer. Throughout reef and deeper soft sediment habitats. Depth 2–25 m.

###### Distribution.

Widespread and abundant in the Indo-Pacific including Mozambique (Tibiriçá et al. 2017), Oman, South Africa, the Philippines, Australia (Debelius 1996), Red Sea (Yonow 1989), Madagascar, Mediterranean Sea, Indonesia, Japan, Papua New Guinea, and Hawaii (Gosliner et al. 2008). Distribution within Thailand currently unclear (see below), previously recorded from the Gulf of Thailand (Mehrotra and Scott 2016).

###### Remarks.

Chavanich et al. (2013) recorded *Hypselodoris infucata* from Andaman and Gulf coasts, and *Hypselodoris obscura* from the Gulf of Thailand. While images were not presented alongside these records, review of the original data hints at a possible clarification. *Hypselodoris obscura* is known to be a sub-tropical species from eastern Australia (Epstein et al. 2018) while *H. infucata* is known to be widespread across the Indo-Pacific; therefore, records from the Gulf of Thailand by Chavanich et al. (2013) are likely to be *H. infucata*. Review of photographs used in the initial identifications supports this clarification, but also suggest that the species called *Hypselodoris infucata* found to be present on both coasts may or may not be the true *H. infucata*. A closer investigation on the distribution of *Hypselodoris* species across both coasts is needed to confirm its range in Thai waters.

##### * Hypselodoris cf. juniperae

Taxon classificationAnimaliaNudibranchiaChromodorididae

Gosliner & Johnson, 2018

C3D17245-B441-57A9-8977-A09FEB1CE034

Figure 11A

###### Material examined.

One specimen 6 mm, CB.

###### Ecology.

Observed under a dead fungiid coral skeleton in shallow coral reef. Depth 6 m.

###### Distribution.

*Hypselodoris juniperae* is currently known from Madagascar (Epstein et al. 2018) and potentially South Africa, Sri Lanka, and Réunion Island (Bidgrain 2005; Ogden 2005; Houben 2007). A similar species represents a first record for Thai waters.

###### Remarks.

Externally similar to both *Hypselodoris maculosa* (Pease, 1871) and *Hypselodoris juniperae* Gosliner & Johnson, 2018 in bearing thin longitudinal white lines, dark purple spots, an opaque orange marginal band and white gills with red apices. Identified as the former species in Mehrotra and Scott (2016). Observed living sympatrically with *H. decorata*, readily distinguished by bearing two red rhinophoral rings and purple rather than reddish brown body colouration. Given the close relationship between all three species, a much closer study is required. Only a single individual has been observed in the area to date.

**Figure 11.**
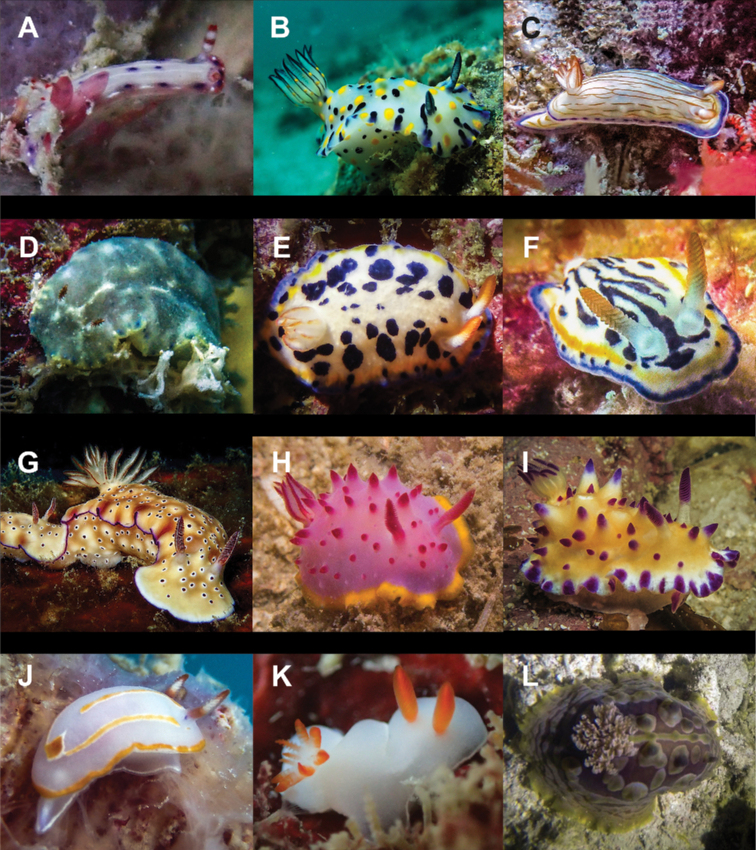
**A**Hypselodoris cf. juniperae 6 mm **B**Hypselodoris cf. kanga 40 mm **C***Hypselodoris katherinae* 15 mm **D**Hypselodoris cf. lacuna 10 mm **E, F**Hypselodoris cf. maritima 20 mm (**E**) and 25 mm (**F**) **G***Hypselodoris tryoni*, right specimen 35 mm (photograph by Kirsty Magson) **H***Mexichromis mariei* 15 mm (photograph by Pau Urgell Plaza) **I***Mexichromis multituberculata* 30 mm (photograph by Pau Urgell Plaza) **J***Mexichromis trilineata* 8 mm **K**Verconia cf. hongkongiensis 6 mm **L***Asteronotus cespitosus* 120 mm (photograph by Emily Palmer).

##### * Hypselodoris cf. kanga

Taxon classificationAnimaliaNudibranchiaChromodorididae

Rudman, 1977

C084886F-BDDE-5FEE-9680-A2512A145720

Figure 11B

###### Material examined.

One specimen 40 mm, TW; two specimens 10–25 mm, SB.

###### Ecology.

Locally found exclusively from deeper soft sediment habitats of the island. Depth 14–25 m.

###### Distribution.

Misidentified by Chavanich et al. (2013) as *H. kanga*, with a recorded distribution in both Andaman Sea and Gulf coasts of Thailand.

###### Remarks.

*Hypselodoris kanga* bears blueish purple lines across its dorsum which are absent in this species, instead replaced by deep blue, almost black spots. These are more abundant towards the margin where they diffuse outwards turning into blue streaks closer to the edge. Given the historic confusion surrounding *H. kanga* (see Epstein et al. 2018), this difference in colouration merits the need for a closer investigation.

##### * Hypselodoris katherinae

Taxon classificationAnimaliaNudibranchiaChromodorididae

Gosliner & Johnson, 2018

BADEA9AA-E6B9-57BF-AE9F-A83BE9FB8D9A

Figure 11C

###### Material examined.

Two specimens 5–15 mm, CB.

###### Ecology.

Observed under dead fungiid coral skeletons and on rocks in shallow coral reef. Depth 3–15 m.

###### Distribution.

Indonesia, Eastern Malaysia, and the Philippines (Gosliner et al. 2008). Here representing a first record for Thai waters.

###### Remarks.

Recorded as undescribed from Koh Tao (Mehrotra and Scott 2016: fig. 2G) now identified as a first record for Thai waters.

##### * Hypselodoris cf. lacuna

Taxon classificationAnimaliaNudibranchiaChromodorididae

Gosliner & Johnson, 2018

1E93918C-B810-5B80-BEE2-1CFE873A3131

Figure 11D

###### Material examined.

One specimen 10 mm, CB.

###### Ecology.

Observed under dead fungiid coral skeletons and on rocks in shallow coral reef. Depth 5 m.

###### Distribution.

*Hypselodoris lacuna* is known from the Philippines, Indonesia, Japan, Papua New Guinea, Vanuatu, and Aldabra Atoll (Gosliner et al. 2008). Here representing a first record for Thai waters is a similar species.

###### Remarks.

Dorsum centrally translucent grey with a network of opaque white lines. Gills grey with light grey apices, rhinophore stalks translucent, clubs white basally turning red with white tips. Mantle edge pale yellow areas with alternating blue spots. Differentiated from *H. lacuna* by having a mostly translucent grey dorsal surface rather than just isolated circles and by the pale yellow areas between the marginal ring of blue spots. Similar to *Hypselodoris* sp. 8 in Gosliner et al. (2018).

##### Hypselodoris cf. maritima

Taxon classificationAnimaliaNudibranchiaChromodorididae

(Baba, 1949)

877CDF1F-9041-5001-B478-E7E31FB0DC8A

Figure 11E, F

###### Material examined.

Three specimens 14–30 mm, TT; two specimens 15–25 mm, SWP; one specimen 20 mm, SR.

###### Ecology.

On rocks and rubble within coral reef. Depth 5–25 m.

###### Distribution.

*Hypselodoris maritima* is recorded from Japan (Baba 1949), Vietnam (Martynov and Korshunova 2012), Taiwan (Su et al. 2009), the Philippines, Indonesia, Hong Kong, Papua New Guinea, and Australia (Gosliner et al. 2008). The species is also recorded as being present in Thailand by Gosliner et al. (2008) but no confirmed records of this or similar species outside of Koh Tao have been found in the literature.

###### Remarks.

Initially recorded as *H. maritima* from Koh Tao (Mehrotra and Scott 2016), further observations have shown variation in external morphology that diverges from the original description. In general, the dorsal surface is always white with scattered and slightly raised spots. While many individuals bear the deep blue to black ‘longitudinal streaks’ along the central dorsal surface, in others these are broken lines or even entirely disconnected spots of varying sizes. Rhinophore clubs range from entirely orange to white with orange apices, stalks always translucent white. The blue marginal band is always separated from the yellow submarginal band by the same white as the dorsum, and both bands are often broken or rows of pigmented spots, matching variation in the dark pigmentation. There are always deep blue-black spots between marginal and submarginal bands/rows, that may vary in size and often extend to the mantle edge. While closer examination may reveal individuals from Koh Tao and the nearby pinnacles to be more than one species, the population is here treated as a single variable species.

##### Hypselodoris tryoni

Taxon classificationAnimaliaNudibranchiaChromodorididae

(Garrett, 1873)

1302417E-C275-5834-93D8-0C6B92021EBC

Figure 11G

###### Material examined.

Two specimens 30–35 mm, CB.

###### Ecology.

On rocks and rubble within coral reef. Depth 1–30 m.

###### Distribution.

Across the Indo-Pacific including Singapore (Toh 2016), Vietnam (Martynov and Korshunova 2012), Australia (Nimbs and Smith 2016), Malaysia, the Philippines, Indonesia, Japan, Palau, Papua New Guinea, Vanuatu, the Marshall Islands (Gosliner et al. 2008), and known from both Andaman and Gulf waters of Thailand (Chavanich et al. 2013).

##### Genus *Mexichromis* Bertsch, 1977

##### * Mexichromis mariei

Taxon classificationAnimaliaNudibranchiaChromodorididae

(Crosse, 1872)

18D1B157-F1D7-58D7-82D7-F97B12E9638C

Figure 11H

###### Material examined.

One specimen 15 mm, SB.

###### Ecology.

In deep soft sediment habitats. Depth 20 m. Feeding on *Dysidea* sp. sponge.

###### Distribution.

Widespread throughout the Indo-Pacific including India (Patel and Apte 2014), Malaysia (Ho 1989), Australia (Nimbs and Smith 2016), South Africa, Madagascar, the Philippines, Indonesia. Japan, Papua New Guinea, and New Caledonia (Gosliner et al. 2008). Here representing a first record for Thai waters.

###### Remarks.

Locally rare with only a single individual observed in the present surveys. Sharing the same habitat and prey preference as *M. multituberculata*.

##### Mexichromis multituberculata

Taxon classificationAnimaliaNudibranchiaChromodorididae

(Baba, 1953)

C25EA656-4820-5CAD-A3BB-F5375C0D5BBD

Figure 11I

###### Material examined.

One specimen 30 mm, SB, one specimen 8 mm, SN.

###### Ecology.

Observed in deep soft sediment habitats throughout the island, though uncommon. Often found associated with or actively feeding on *Dysidea* sp. sponge which grows unattached on the benthos. Depth 14–25 m.

###### Distribution.

Widespread throughout the Indo-Pacific including, India (Kumar et al. 2011), Myanmar (Sanpanich and Duangdee 2019), Vietnam (Martynov and Korshunova 2012), Taiwan (Huang et al. 2015), Hong Kong (Rudman and Darvell 1990), China (Lin 1990), the Philippines, Indonesia, and Japan (Gosliner et al. 2008). Recorded from both Andaman and Gulf waters of Thailand (Chavanich et al. 2013).

##### Mexichromis trilineata

Taxon classificationAnimaliaNudibranchiaChromodorididae

(A. Adams & Reeve, 1850)

46A7B034-FA63-5655-8806-1262A5F1237B

Figure 11J

###### Material examined.

Three specimens 5–8 mm, CB; two specimens 5 mm, TW.

###### Ecology.

Usually found immersed in prey sponge *Dysidea* sp. under rubble and dead fungiid coral skeletons in shallow coral reef habitats. Uncommon, though multiple individuals may be observed together. Depth 3–8 m.

###### Distribution.

Across the western Pacific including Indonesia (Yonow 2001; 2017), the Philippines, Palau, Papua New Guinea, and Australia (Gosliner et al. 2008). Recorded from the Gulf of Thailand (Mehrotra and Scott 2016). Very variable in pattern.

##### Genus *Verconia* Pruvot-Fol, 1931

##### * Verconia cf. hongkongiensis

Taxon classificationAnimaliaNudibranchiaChromodorididae

(Rudman, 1990)

697BECC4-95F5-585E-A629-27716C1B7EC1

Figure 11K

###### Material examined.

One specimen 6 mm, CB; one specimen 4 mm, TW.

###### Ecology.

Observed under dead fungiid coral skeleton and rubble in shallow coral reef. Depth 3–8 m.

###### Distribution.

*Verconia hongkongiensis* is known from Japan and Hong Kong (Gosliner et al. 2008, 2018). The present Verconia cf. hongkongiensis is a first record for Thai waters.

###### Remarks.

Initially recorded as *Hypselodoris bullockii* (Collingwood, 1881) from a single small specimen by Mehrotra and Scott (2016), further observations have concluded that the species from Koh Tao is not *H. bullockii* but one superficially resembling *Verconia hongkongiensis* (Rudman 1990). Similarities between the two species are the thin marginal white line and pale variations in colour of the dorsum. However, rather than reddish tips, gills appear to be uniformly red (more orange in some specimens) and rhinophores appear pigmented throughout, basally red, sometimes with a paler median band, ending in red/orange tips, with all individuals recorded from Koh Tao being smaller than 6 mm.

#### Family Discodorididae Bergh, 1891

**Genus *Asteronotus* Ehrenberg, 1831**

##### * Asteronotus cespitosus

Taxon classificationAnimaliaNudibranchiaDiscodorididae

(van Hasselt, 1824)

4DCDDF2C-5948-5D32-B96D-5ED2315029EE

Figure 11L

###### Material examined.

One specimen 120 mm, CB.

###### Ecology.

Locally found exclusively in soft sediment habitats. Depth 1–16 m.

###### Distribution.

Widespread Indo-Pacific including Australia, Indonesia, Mauritius (Gosliner et al. 2008), Red Sea (Yonow 1990), Hawaii (Kay and Young 1969), Mozambique (Tibiriçá et al. 2017), and Gulf of Thailand (Chavanich et al. 2013).

##### Genus *Atagema* Gray, 1850

##### * Atagema intecta

Taxon classificationAnimaliaNudibranchiaDiscodorididae

(Kelaart, 1859)

B5E7BD1D-E7FE-5FCD-A345-593BDDF74591

Figure 12A

###### Material examined.

One specimen 25 mm, CB.

###### Ecology.

Coral reefs. Depth 5–10 m.

###### Distribution.

Widespread Indo-Pacific including the Red Sea (Yonow 2008), Mozambique (Tibiriçá et al. 2017), Sri Lanka (Kelaart 1859), Indonesia (Debelius 1996), Australia (Nimbs and Smith 2016), Japan, Papua New Guinea, and Hawaii (Gosliner et al. 2008). Here representing a first record for Thai waters.

**Figure 12.**
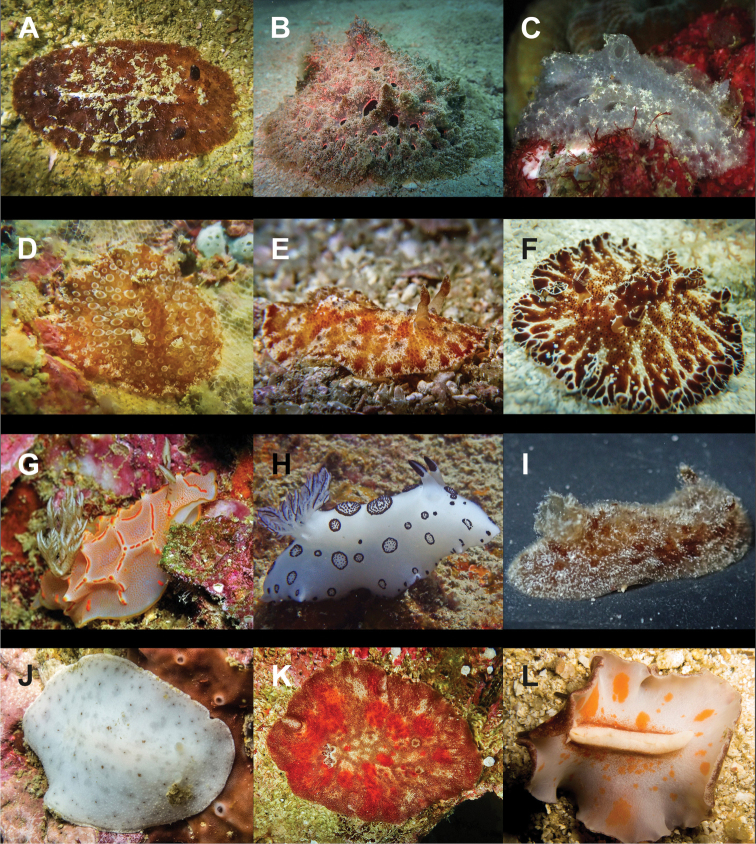
**A***Atagema intecta* 25 mm (photograph by Nick Tringham) **B***Atagema spongiosa* 95 mm **C***Atagema* sp. 32 mm **D**Carminodoris cf. bifurcata 9 mm **E***Discodoris cebuensis* 30 mm (photograph by Kirsty Magson) **F***Discodoris boholiensis* 55 mm **G***Halgerda bacalusia* (unknown size, photograph by Stephan Pelletier) **H***Jorunna funebris* 87 mm **I***Jorunna* sp. 10 mm **J***Peltodoris murrea* 30 mm **K, L**Platydoris cf. formosa 35 mm, dorsal and ventral views (photographs by Kirsty Magson).

##### * Atagema spongiosa

Taxon classificationAnimaliaNudibranchiaDiscodorididae

(Kelaart, 1858)

F99FBA8F-8254-52BC-ABF1-A65B971CF45A

Figure 12B

###### Material examined.

One specimen 95 mm, CB.

###### Ecology.

Observed upon prey sponge *Dysidea* sp. in deeper soft sediment habitats. Depth 14–24 m.

###### Distribution.

Gulf of Oman (Fatemi and Attaran 2015), Red Sea (Yonow 2008), India (Apte et al. 2010), Sri Lanka (Kelaart 1858), Australia (Nimbs and Smith 2016), Fiji (Brodie and Brodie 1990), Madagascar, Singapore, the Philippines, South Korea, and Papua New Guinea (Gosliner et al. 2008). Here representing a first record for Thai waters.

##### * Atagema

Taxon classificationAnimaliaNudibranchiaDiscodorididae

sp. (Kelaart, 1858)

63512E7E-2CDA-53F3-B762-B7698BD7228E

Figure 12C

###### Material examined.

One specimen 32 mm, CB.

###### Ecology.

Observed among rubble in shallow coral reef habitats at night with no observed association with prey. Depth 4–6 m.

###### Distribution.

*Atagema* sp. 8 (Gosliner et al. 2018) is known only from the Philippines.

###### Remarks.

Externally distinct from *Atagema spongiosa* (Kelaart, 1858) in being completely translucent pale grey, including the circular pits, separated by ridges, along the mantle. Similar to *Atagema* sp. 8 (Gosliner et al. 2018). Individuals from Koh Tao also differ from *A. spongiosa* in ecology, being observed in shallow reef environments instead of deeper soft sediment habitats.

##### Genus *Carminodoris* Bergh, 1889

##### * Carminodoris cf. bifurcata

Taxon classificationAnimaliaNudibranchiaDiscodorididae

Baba, 1993

E24AD215-25D0-51A7-A16F-FE43C4C2C518

Figure 12D

###### Material examined.

Two specimens 9–17 mm, HF.

###### Ecology.

Among rubble in shallow coral reef habitats. Depth 3–8 m.

###### Distribution.

*Carminodoris bifurcata* is recorded across the Indo-Pacific including from Mozambique (Tibiriçá et al. 2017), the Red Sea (Yonow 2008), Korea (Koh 2006), the Philippines, Japan, and Hawaii (Gosliner and Fahey 2011) and *Carminodoris flammea* is recorded from Indonesia (Gosliner and Fahey 2011). Neither species has been previously recorded from Thai waters.

###### Remarks.

Specimens from Koh Tao resemble both *Carminodoris bifurcata* Baba 1993 and *Carminodoris flammea* (Gosliner and Fahey 2011). They differ from the descriptions of the former by lacking any black spots on the dorsum and having a brown rather than grey ground colour and they differ from the latter in having brown rather than grey gill leaves, with white tips, and a tan median colouration instead of the bright red for which the species is named. A very similar looking specimen from Vietnam was identified as *Hoplodoris bifurcata* by Martynov and Korshunova (2012: pl. 31E). However, see discussions regarding *Carminodoris pustulata* (Abraham, 1877) by Jensen (1994), Yonow et al. (2002), and Yonow (2017) and indeed by Baba in the original description.

##### Genus *Discodoris* Bergh, 1877

##### * Discodoris cebuensis

Taxon classificationAnimaliaNudibranchiaDiscodorididae

Bergh, 1877

C6FE4668-2FA7-52F0-806F-1E60E65276CF

Figure 12E

###### Material examined.

One specimen 30 mm, SN.

###### Ecology.

Locally found exclusively in soft sediment habitats. Depth 14–18 m.

###### Distribution.

Across the Indo-Pacific including the Red Sea (Yonow 2008), South Africa (Gosliner 1987), Seychelles (Eliot 1910), Tanzania, the Philippines, Indonesia, Japan, Papua New Guinea, and Hawaii (Dayrat 2010). Here representing a first record for Thai waters.

##### Discodoris boholiensis

Taxon classificationAnimaliaNudibranchiaDiscodorididae

Bergh, 1877

A1F530E7-CCD8-5F0F-B87D-4F2796FFEB8D

Figure 12F

###### Material examined.

One specimen 55 mm, SD.

###### Ecology.

Found exclusively in soft sediment habitats. Depth 12–24 m.

###### Distribution.

Widespread across the Indo-Pacific including India (Rao 1960), Vietnam (Risbec 1956), Singapore (Lim and Chou 1970), New Caledonia (Risbec 1928), Vanuatu (Coleman 2001), Madagascar, Indonesia, the Philippines, Palau, Papua New Guinea, Australia (Dayrat 2010), and the Gulf of Thailand (Chavanich et al. 2013).

##### Genus *Halgerda* Bergh, 1880

##### Halgerda bacalusia

Taxon classificationAnimaliaNudibranchiaDiscodorididae

Fahey & Gosliner, 1999

8BB7CA96-0804-5D70-9C68-04B20C3CBE12

Figure 12G

###### Material examined.

No specimen collected.

###### Ecology.

Shallow coral reef habitats. Depth 5–10 m.

###### Distribution.

The range of this species appears to be very limited thus far including only Myanmar (Gosliner et al. 2018) and Thailand, from both the Andaman coast (Fahey and Gosliner 1999) and Gulf of Thailand (Mehrotra and Scott 2016).

###### Remarks.

Recorded at Koh Tao from a single individual in 2011 and not recorded since. The included figure (Fig. 12G) represents the only evidence of the species from the Gulf of Thailand. This largely agrees with the observations of the authors that specimens of the genus *Halgerda* are thus far exceptionally rare from within the Gulf of Thailand.

##### Genus *Jorunna* Bergh, 1876

##### Jorunna funebris

Taxon classificationAnimaliaNudibranchiaDiscodorididae

(Kelaart, 1859)

E7E727B1-6E3D-5837-9A86-1E160D981B52

Figure 12H

###### Material examined.

Two specimens 15–25 mm, LB; one specimen 30 mm, SI; one specimen 87 mm, CB.

###### Ecology.

Abundant throughout corals and rubble in both nearshore reefs and offshore pinnacles. Rarely observed in soft sediment habitats. Depth 2–35 m; preys on blue *Xestospongia* sp. (Huang et al. 2016).

###### Distribution.

Widespread in the Indo-Pacific including the Red Sea (Yonow 2008), India (Apte 2009), Sri Lanka (Kelaart 1859), Indonesia (Yonow 2011), Australia (Debelius 1996) Mauritius, Madagascar, Philippines, Japan, Palau, New Caledonia (Camacho-García and Gosliner 2008a), Malaysia (Ho 1989), Vietnam (Risbec 1956), and known from both Andaman and Gulf waters of Thailand (Chavanich et al. 2013).

##### * Jorunna

Taxon classificationAnimaliaNudibranchiaDiscodorididae

sp.

6047B869-2EBC-5021-95E1-E81AE5C37378

Figure 12I

###### Material examined.

Two specimens 10–15 mm, SB.

###### Ecology.

Found exclusively in deeper soft sediment habitats. Depth 16–25 m.

###### Distribution.

*Jorunna* sp. 7 recorded in the Philippines (Gosliner et al. 2018).

###### Remarks.

An undescribed species covered in numerous long caryophyllidia, similar to *Jorunna* sp. 7 in Gosliner et al. (2018). Cream coloured dorsum with dark brown patches, white pigment on most caryophyllidia, dark brown rhinophore clubs with lamellae edged in white and white apices. Gills cream with some white. Here representing a first record for Thai waters.

##### Genus *Peltodoris* Bergh, 1880

##### * Peltodoris murrea

Taxon classificationAnimaliaNudibranchiaDiscodorididae

(Abraham, 1877)

F18583DF-F8A2-5CC2-B892-5503BE90AE76

Figure 12J

###### Material examined.

One specimen 30 mm, SR.

###### Ecology.

Documented here from a single record found upon submerged concrete artificial substrate at an offshore pinnacle site. Depth 30 m.

###### Distribution.

Widespread in the Indo-Pacific including the Red Sea (Yonow 2008), New Caledonia, Australia, Japan (Dayrat 2010), Indonesia, and Malaysia (Yonow 2017). Here representing a first record for Thai waters.

##### Genus *Platydoris* Bergh, 1877

##### * Platydoris cf. formosa

Taxon classificationAnimaliaNudibranchiaDiscodorididae

(Alder & Hancock, 1864)

FBFF0F68-0C4C-569B-9616-BF6A7AFDC7F9

Figure 12K, L

###### Material examined.

One specimen 20 mm, CA; two specimens 35–40 mm, SI.

###### Ecology.

Among rubble at the coral reef edge. Depth 8–16 m.

###### Distribution.

*Platydoris formosa* is known from Australia (Nimbs and Smith 2016), Tanzania, India, Indonesia, the Philippines, Samoa, New Caledonia, and Hawaii (Gosliner et al. 2008). *Platydoris cinereobranchiata*Dorgan et al., 2002 is known from the Philippines, Australia, the Solomon Islands (Gosliner et al. 2008), and Indonesia (Yonow 2011). Neither species has yet been documented in Thai waters.

###### Remarks.

Externally, the specimens bear similarity to *Platydoris formosa*, as detailed by Dorgan et al. (2002) although differ in numerous ways. The rhinophores of the present species are yellow to pale brown, sometimes with red spots near the apex, with no black spots between them. Rhinophores have 33–35 lamellae. The gills are grey with dark brownish red lines running along the rachises, with no black spots anterior to the gill sheath. A white ring is present around the rims of the rhinophore and gill sheaths. Most of the caryophyllidia covering the dorsum are a dark reddish colour with a few white patches randomly distributed. Larger bright red patches are more numerous and also randomly distributed across the dorsal surface. Ventrally white with large red spots which become more diffuse and concentrated towards the foot. Based on these characteristics, it appears that the specimens from Koh Tao bear characteristics of both *P. formosa* and *P. cinereobranchiata*.

##### Genus *Rostanga* Bergh, 1879

##### * Rostanga

Taxon classificationAnimaliaNudibranchiaDiscodorididae

sp.

2E634E51-98A4-5FCB-AB8D-C1787E75BCB2

Figure 13A

###### Material examined.

Two specimens 6–18 mm, CB.

###### Ecology.

Exclusively found under coral rubble and the skeletons of dead Fungiidae corals. Cryptic on its pink-red sponge. Depth 3–8 m.

###### Distribution.

Unknown.

###### Remarks.

The present species is only identified based on external morphology and as such has not been identified to species level. Chavanich et al. (2013) documented *Rostanga orientalis* Rudman & Avern, 1989 from the Gulf of Thailand, which bears some external similarities to the present specimens.

**Figure 13.**
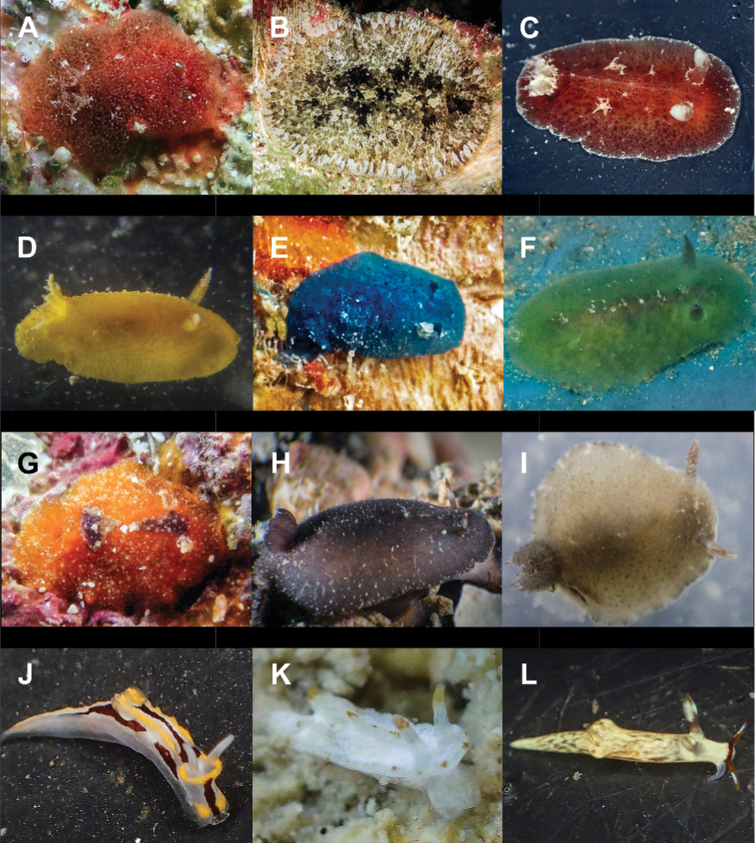
**A***Rostanga* sp. 18 mm **B***Sebadoris fragilis* 70 mm **C***Thordisa* sp. 12 mm **D**Doriopsis cf. granulosa 7 mm **E***Doriopsis pecten* 6 mm **F***Doriopsis viridis* 4 mm **G**Doris cf. immonda 5 mm **H, I**Dorididae sp. 15 mm (**H** photograph by Jeremy Coz) and 8 mm (**I**) **J***Goniodoridella* sp. 1 6 mm **K***Goniodoridella* sp. 2 3 mm (photograph by Khumron Waipaka) **L**Trapania cf. gibbera 5 mm.

##### Genus *Sebadoris* Er. Marcus & Ev. Marcus, 1960

##### * Sebadoris fragilis

Taxon classificationAnimaliaNudibranchiaDiscodorididae

(Alder & Hancock, 1864)

42C7B48F-C920-5958-B50E-48DDB60A435E

Figure 13B

###### Material examined.

One specimen 70 mm, SB.

###### Ecology.

Found under coral rubble and the skeletons of dead Fungiidae corals. Depth 3–8 m.

###### Distribution.

Across the Indo-Pacific including Madagascar (Gosliner et al. 2008), Red Sea (Yonow 2008), Kuwait (Nithyanandan 2012), India (Bhave and Apte 2011), Indonesia, the Philippines, Papua New Guinea (Yonow 2017), and known from the Gulf of Thailand (Chavanich et al. 2013).

##### Genus *Thordisa* Bergh, 1877

##### * Thordisa

Taxon classificationAnimaliaNudibranchiaDiscodorididae

sp.

6FF3ECE8-8851-553F-82E0-6C4F2715EE01

Figure 13C

###### Material examined.

One specimen 12 mm, TT.

###### Ecology.

Found in soft sediment habitats at 16 m depth.

###### Distribution.

Unknown.

###### Remarks.

Dorsal colour deep red, with white rhinophore clubs and gills. The most distinctive feature of the species appears to be a pair of distinct, elongated, white, conical papillae surrounded by 5–7 white extensions radiating out at the base of each papilla, on either side of the mid-dorsal ridge. Besides these are a few smaller but still elongated white papillae randomly distributed. Additionally, the base of the white gills and translucent red rhinophore stalks is surrounded by a thin white line which similarly can be found around the margin of the mantle. The most similar known species might be *T. sanguinea* Baba, 1955 which may be distinguished based on the ground colour, dorsal ornamentation (covered in papillae), and the colour of the rhinophores and gills.

#### Family Dorididae Rafinesque, 1815

**Genus *Doriopsis* Pease, 1860**

##### * Doriopsis cf. granulosa

Taxon classificationAnimaliaNudibranchiaDorididae

(Pease, 1860)

5BD60FBE-3854-5621-A298-24AE0276A387

Figure 13D

###### Material examined.

One specimen 7 mm, HF.

###### Ecology.

Under reef rubble and on rocks in coral reef habitats. Cryptic on its yellow prey sponge. Depth 6–12 m.

###### Distribution.

*Doriopsis granulosa* is found across the Indo-Pacific including India (Apte and Bhave 2014), Seychelles (Gosliner et al. 2008), Tanzania (Edmunds 1971), Japan (Baba and Hamatani 1961), Hong Kong (Orr 1981), Australia (Nimbs and Smith 2016), and Hawaii (Kay and Young 1969). Here representing a first record for Thai waters.

###### Remarks.

While clearly resembling *Doriopsis granulosa* (Pease, 1860) by an overall yellow dorsum, six gill leaves arranged horizontally in an almost transverse line, and numerous low, rounded tubercles, it bears differences from other descriptions (see Baba and Hamatani 1961; Valdés 2002; Apte and Bhave 2014). These include the absence of any brown spots along the dorsum and rhinophores that are not uniformly yellow-orange but instead have translucent stalks and abruptly transition from a yellow base to a white apex, separated by a thin brown median line. These subtle differences may be found to be within the variability of the species and cannot be confirmed without a closer investigation.

##### * Doriopsis pecten

Taxon classificationAnimaliaNudibranchiaDorididae

Collingwood, 1881

7E649478-00F9-50B2-826D-B3A4A2A70D89

Figure 13E

###### Material examined.

Two specimens 6 mm, CB.

###### Ecology.

Among rubble in coral reef habitats. Depth 3–6 m.

###### Distribution.

Across the Indo-Pacific including Taiwan (Collingwood 1881), Vietnam (Martynov and Korshunova 2012), the Philippines, Indonesia (Yonow 2017), New Caledonia (Risbec 1953), South Africa, Madagascar, and Hawaii (Gosliner et al. 2008). Previously documented from the Gulf of Thailand (Chavanich et al. 2013).

##### * Doriopsis viridis

Taxon classificationAnimaliaNudibranchiaDorididae

(Pease, 1861)

BD6BD941-521E-5AC6-A24D-5265BC397E80

Figure 13F

###### Material examined.

Two specimens 4–6 mm, CB.

###### Ecology.

Among rubble in coral reef habitats. Depth: 3–6 m.

###### Distribution.

Known from China (Lin 1990), Vietnam (Risbec 1956), Tahiti, Hawaii, and western Mexico (Gosliner et al. 2008). Here representing a first record for Thai waters.

##### Genus *Doris* Linnaeus, 1758

##### * Doris cf. immonda

Taxon classificationAnimaliaNudibranchiaDorididae

Risbec, 1928

CFCAFE4C-E2BA-56A2-9064-6138FE147E86

Figure 13G

###### Material examined.

One specimen 5 mm, HF.

###### Ecology.

Under reef rubble and on rocks in coral reef habitats. Cryptic on its orange prey sponge. Depth 6–12 m.

###### Distribution.

*Doris immonda* is known across the Indo-Pacific including Japan (Rudman 2000a), Australia (Nimbs and Smith 2016), Papua New Guinea, New Caledonia, Hawaii (Gosliner et al. 2008), and the Pacific coast of Costa Rica (Camacho-García and Gosliner 2008b). Here representing a first record for Thai waters.

###### Remarks.

Broadly matches the description and variations highlighted by Valdés (2002); however, like specimens of D. cf. granulosa, specimens documented from Koh Tao differ in colouration from *Doris immonda*. The dorsal colour is a bright orange with numerous rounded or conical tubercles covering the surface, many capped in white. The Y-shaped marking between the rhinophores to in front of the gills is made up of purple tubercles and the rhinophore club is a pale brown with white edges of lamellae. As above, this variation in colouration may yet be considered to be within what can be found in *Doris immonda* Risbec, 1928.

##### * Dorididae

Taxon classificationAnimaliaNudibranchiaDorididae

sp.

C65D9343-DAAA-5768-A864-C81C3183B264

Figure 13H, I

###### Material examined.

Three specimens 6–15 mm, SN.

###### Ecology.

In soft sediment habitats beyond the coral reef.

###### Distribution.

Unknown. Currently only documented from Koh Tao.

###### Remarks.

A small dorid with a dark mantle ranging from grey to dark brown, covered in numerous small, clearly separated pustules. Gills arranged circularly, pinnate, dark brown. The lamellate rhinophores are basally dark brown with translucent white clubs and reddish brown apices with white tips. A much more in-depth analysis of this species is needed to ascertain its placement.

#### Superfamily Onchidoridoidea Gray, 1827

**Family Goniodorididae H. Adams & A. Adams, 1854**

##### Genus *Goniodoridella* Pruvot-Fol, 1933

###### Goniodoridella

Taxon classificationAnimaliaNudibranchiaGoniodorididae

sp. 1

14BE2ACC-AA61-571B-B4EF-3AB5624EC40E

Figure 13J

####### Material examined.

One specimen 5 mm, TT; two specimens 6–8 mm, CA.

####### Ecology.

Rare and cryptic within coral reef habitats. Depth 8–24 m.

####### Distribution.

Similar to *Goniodoridella* sp. 2 (Gosliner et al. 2008) which is known only from the Philippines and Papua New Guinea. First documented from the Gulf of Thailand by Mehrotra and Scott (2016).

###### * Goniodoridella

Taxon classificationAnimaliaNudibranchiaGoniodorididae

sp. 2

E87A636C-081F-5AFF-BC03-85982667E286

Figure 13K

####### Material examined.

One specimen 3 mm, SI.

####### Ecology.

Rare and cryptic within coral reef habitats. Depth 14 m.

####### Distribution.

Similar to *Goniodoridella* sp. 10 (Gosliner et al. 2018) which is known only from the Indonesia. Here documented as a first record for Thai waters.

##### Genus *Trapania* Pruvot-Fol, 1931

###### * Trapania cf. gibbera

Taxon classificationAnimaliaNudibranchiaGoniodorididae

Gosliner & Fahey, 2008

CBF38768-82B6-5C22-8598-FF1A1A0C2D88

Figure 13L

####### Material examined.

One specimen 5 mm, CB.

####### Ecology.

Under rubble in shallow coral reefs. Depth 4–6 m.

####### Distribution.

*Trapania gibbera* is known from Indonesia, Japan, and Papua New Guinea (Gosliner and Fahey 2008). Here representing a first record for Thai waters.

####### Remarks.

Specimens from Koh Tao differ slightly from *Trapania gibbera*Gosliner and Fahey 2008 in colouration. The ‘hump’ located anterior to the gills, for which *T. gibbera* was named, is also seen in specimens from Koh Tao. The dorsal colouration is tan rather than white with numerous reddish brown patches spread over the body, sometimes giving animals a reticulated appearance. Rhinophore clubs have nine lamellae that are mostly white with some edges being red, with translucent red stalks and red apices. A deep red mark can be seen at the anterior margin of the head that extends to the oral tentacles, which turn orange-brown and have a single distinctive white spot on the dorsal surface of each. Ventrally the oral tentacles are entirely orange.

###### * Trapania miltabrancha

Taxon classificationAnimaliaNudibranchiaGoniodorididae

Gosliner & Fahey, 2008

92757309-F1A2-532C-BBFA-8BDC445B0678

Figure 14A, B

####### Material examined.

Three specimens 8–25 mm, LB.

####### Ecology.

Among colonies of Didemnid tunicates in soft sediment habitats. Depth 12–15 m.

####### Distribution.

Known only from Indonesia (Gosliner and Fahey 2008) and Japan (Uyeno and Nagasawa 2012). Here representing a first record for Thai waters

####### Remarks.

While known to be predators of Entoprocta (Gosliner et al. 2018), the present individuals were all found directly upon *Didemnum molle* tunicate colonies in soft sediment habitats. While active feeding could not be confirmed, it is of interest to note that all 12 individuals recorded were initially found crawling upon the tunicates and not upon the benthos which may indicate the presence of a prey source associated with the tunicates but feeding investigations were not carried out.

**Figure 14.**
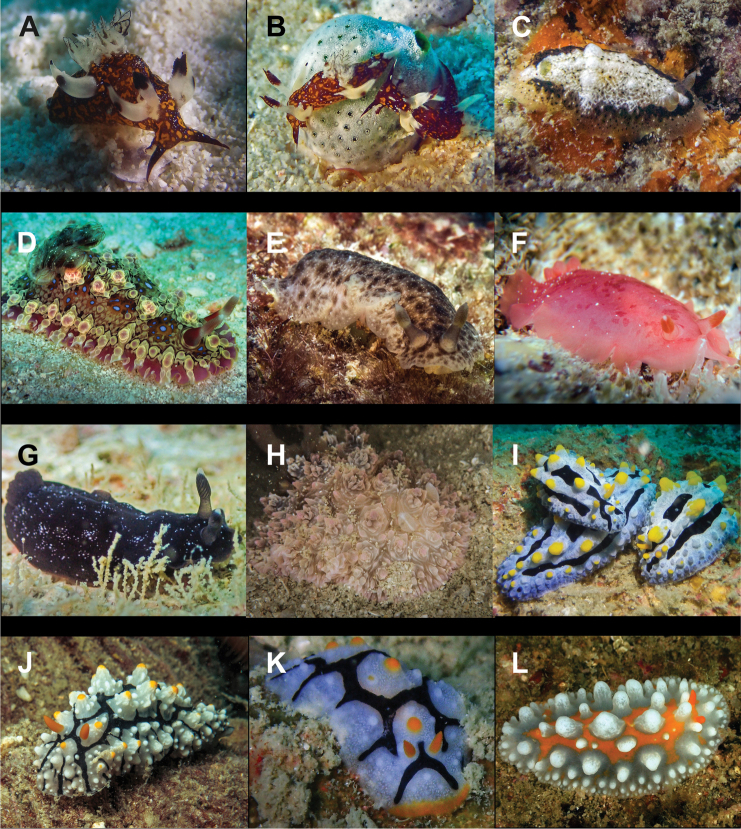
**A, B***Trapania miltabrancha* 25 mm (**A**) two specimens on *Didemnum molle* (**B**) **C***Dendrodoris coronata* 30 mm (photograph by Kirsty Magson) **D***Dendrodoris krusensternii* 45 mm **E***Dendrodoris elongata* 29 mm **F***Dendrodoris fumata* 55 mm **G***Dendrodoris nigra* 16 mm **H***Dendrodoris tuberculosa* 88 mm **I***Phyllidia coelestis* specimens 25–35 mm **J***Phyllidia elegans* 45 mm (photograph by Pau Urgell Plaza) **K***Phyllidia exquisita* 30 mm **L***Phyllidia ocellata* 60 mm.

#### Superfamily Phyllidioidea Rafinesque, 1814

**Family Dendrodorididae O’Donoghue, 1924 (1864)**

##### Genus *Dendrodoris* Ehrenberg, 1831

###### * Dendrodoris coronata

Taxon classificationAnimaliaNudibranchiaDendrodorididae

Kay & Young, 1969

BD114ACF-88AD-5EAC-8061-01A3E4F39B6A

Figure 14C

####### Material examined.

One specimen 30 mm, LT.

####### Ecology.

On rocks and among corals in shallow reefs. Depth 2–6 m.

####### Distribution.

Widespread in the Indo-Pacific including the Red Sea (Yonow 2008), Mozambique (Tibiriçá et al. 2017), Japan, Australia, Palau, Papua New Guinea, New Caledonia, Marshall Islands (Gosliner et al. 2008), probably Indonesia (Yonow 2017), and Hawaii (Kay and Young 1969). Here representing a first record for Thai waters.

###### Dendrodoris krusensternii

Taxon classificationAnimaliaNudibranchiaDendrodorididae

(Gray, 1850)

D007F7AB-3AA6-5F43-8750-AF7381B35DD6

Figure 14D

####### Material examined.

Three specimens 8–45 mm, TT; one specimen 22 mm, SB.

####### Ecology.

Exclusively recorded from soft sediment habitats beyond the fringing coral reef. Depth 14–26 m.

####### Distribution.

Widespread in the Indo-Pacific including the Red Sea (Yonow 2015), Mozambique (Tibiriçá et al. 2017), South Africa, Hawaii (Gosliner 1987), Singapore, the Philippines, Indonesia, Korea, Papua New Guinea, New Caledonia, New Zealand (Gosliner et al. 2008), Japan (Baba 1949), Australia (Angas 1864) and known from both Andaman and Gulf waters of Thailand (Chavanich et al. 2013).

###### * Dendrodoris elongata

Taxon classificationAnimaliaNudibranchiaDendrodorididae

Baba, 1936

FF8EC7A4-9305-572D-8150-DB7E0249F708

Figure 14E

####### Material examined.

Three specimens 29–42 mm, CB.

####### Ecology.

Found under coral rubble and the skeletons of dead Fungiidae corals. Depth 3–8 m.

####### Distribution.

Across the Indo-Pacific including the Red Sea (Yonow 2008), India (Vadher and Kardani 2018), Vietnam (Risbec 1956), China (Lin 1990), Malaysia, the Philippines, Australia, New Caledonia, Fiji (Gosliner et al. 2008), and Indonesia (Yonow 2017). Here representing a first record for Thai waters.

###### * Dendrodoris fumata

Taxon classificationAnimaliaNudibranchiaDendrodorididae

(Rüppell & Leuckart, 1830)

6D69ED00-1FCA-5332-92CB-F19C3E148A3C

Figure 14F

####### Material examined.

One specimen 7 mm, SB; one specimen 55 mm, CB.

####### Ecology.

Found under coral rubble and the skeletons of dead fungiid corals. Depth 3–8 m

####### Distribution.

Widespread in the Indo-Pacific including South Africa, Tanzania, Madagascar, Malaysia, Palau, Vanuatu, New Caledonia, Hawaii (Gosliner et al. 2008), Red Sea (Yonow 2008), Mauritius, Socotra, Persian Gulf, La Réunion (Yonow 2012), Hong Kong, Papua New Guinea, Fiji, Australia (Brodie et al. 1997), Indonesia (Yonow 2017), and known from the Gulf of Thailand (Chavanich et al. 2013).

###### Dendrodoris nigra

Taxon classificationAnimaliaNudibranchiaDendrodorididae

(Stimpson, 1855)

4B13E335-536A-5A90-9445-D19F6ABED9F7

Figure 14G

####### Material examined.

Two specimens 8–16 mm, LT; one specimen 25 mm, MH.

####### Ecology.

Found under coral rubble and the skeletons of dead Fungiidae corals. More abundant towards the edge of the reef, less abundant but present in soft sediment habitats outside of the coral reef. Depth 2–25 m.

####### Distribution.

Widespread in the Indo-Pacific including Mozambique (Tibiriçá et al. 2017), Mauritius (Yonow and Hayward 1991), Red Sea (Yonow 1990), South Africa (Gosliner 1987), Socotra, Maldives, Zanzibar, Gulf of Oman, Seychelles, La Réunion, (Yonow 2012), India (Apte 2009), Gulf of Oman (Fatemi and Attaran 2015), Indonesia (Yonow 2017), Vietnam (Risbec 1956), Japan (Stimpson 1855), Australia (Burn 2006), Hawaii (Kay and Young 1969), and known from both Andaman and Gulf waters of Thailand (Jensen 1998; Chavanich et al. 2013).

###### Dendrodoris tuberculosa

Taxon classificationAnimaliaNudibranchiaDendrodorididae

(Quoy & Gaimard, 1832)

4F25072C-5D45-50C6-95A5-2FBEF8ACD880

Figure 14H

####### Material examined.

Two specimens 24–88 mm, TT.

####### Ecology.

Found in soft sediment habitats outside the coral reef alongside *D. krusensternii*. Incorrectly classified as a coral reef-associated species from Koh Tao (Mehrotra and Scott 2016) based on a single observation in shallower waters nearer the coral reef.

####### Distribution.

Widespread in the Indo-Pacific including the Red Sea (Yonow 2008), Tanzania, South Africa, Maldives, Malaysia, Philippines, Korea, Papua New Guinea, Australia, Solomon Islands, Marshall Islands (Gosliner et al. 2008), Mozambique (Tibiriçá et al. 2017), India (Apte 2009), Chagos (Yonow et al. 2002), Vietnam (Risbec 1956), Japan (Baba 1949), Hawaii (Kay and Young 1969), and known from both Andaman and Gulf waters of Thailand (Chavanich et al. 2013; Mehrotra and Scott 2016).

#### Family Phyllidiidae Rafinesque, 1814

**Genus *Phyllidia* Cuvier, 1797**

##### Phyllidia coelestis

Taxon classificationAnimaliaNudibranchiaPhyllidiidae

Bergh, 1905

22829CB0-6A23-50A2-B215-BE412C714ABD

Figure 14I

###### Material examined.

Three specimens 25–35 mm, GR.

###### Ecology.

Abundant in coral reef habitats. Depth 3–30 m

###### Distribution.

Widespread in the Indo-Pacific including Mozambique (Tibiriçá et al. 2017), South Africa (Brunckhorst 1993), Madagascar, Seychelles (Yonow 2012), Tanzania (Edmunds 1972), Sri Lanka (Yonow 1984), Chagos Islands (Yonow et al. 2002), India (Apte 2009), the Philippines, Japan, Papua New Guinea (Gosliner et al. 2008), Australia (Nimbs and Smith 2016), China (Lin 1990), Indonesia (Yonow 2011), and known from both Andaman and Gulf waters of Thailand (Brunckhorst 1993; Chavanich et al. 2013).

##### Phyllidia elegans

Taxon classificationAnimaliaNudibranchiaPhyllidiidae

Bergh, 1869

3037B758-BE11-56EC-BBC6-BBCB852C4642

Figure 14J

###### Material examined.

One specimen 45 mm, LT; one specimen 30 mm, RR; one specimen 45 mm, SP.

###### Ecology.

Abundant in coral reef habitats. Depth 3–30 m

###### Distribution.

Widespread in the western Pacific including), Indonesia, Taiwan, Australia, Guam, Solomon Islands (Brunckhorst 1993), Myanmar, Malaysia, the Philippines, Japan, Fiji, Papua New Guinea, Vanuatu (Gosliner et al. 2008), Vietnam (Risbec 1956), China (Lin 1990), Singapore (Lim and Chou 1970) and known from both Andaman and Gulf waters of Thailand (Brunckhorst 1993; Chavanich et al. 2013).

##### * Phyllidia exquisita

Taxon classificationAnimaliaNudibranchiaPhyllidiidae

Brunckhorst, 1993

4A17BCC6-AA77-5FEF-8CC2-DA96730FDF00

Figure 14K

###### Material examined.

One specimen 30 mm, SI.

###### Ecology.

Rare, found in coral reef habitats, Depth 15 m.

###### Distribution.

Known from the Maldives (Yonow 2012), Vietnam (Martynov and Korshunova 2012), Hong Kong (Orr 1981), Malaysia, Indonesia, Philippines, Palau, Japan (Gosliner et al. 2008), Australia, Papua New Guinea, Fiji, the Marshall Islands, and the Andaman sea of Thailand (Brunckhorst 1993). Here representing a first record for the Gulf of Thailand.

##### Phyllidia ocellata

Taxon classificationAnimaliaNudibranchiaPhyllidiidae

Cuvier, 1804

2A93CF2A-5404-5DE1-8C33-3A635D84C0C9

Figure 14L

###### Material examined.

One specimen 30 mm, AMN; one specimen 65 mm, SP; three specimens 45–60 mm, HF.

###### Ecology.

Abundant in coral reef, reef edge, and soft sediment habitats. Depth 3–30 m.

###### Distribution.

Widespread in the Indo-Pacific including Mozambique (Tibiriçá et al. 2017), Oman, Sri Lanka, the Philippines (Debelius 1996), India (Ramakrishna et al. 2010), Myanmar (Sanpanich and Duangdee 2019), Indonesia (Yonow 1996), South Africa, Mauritius, Madagascar, Tanzania, Japan, Papua New Guinea, Australia, Guam, Fiji, Vanuatu (Gosliner et al. 2008) and known from both Andaman and Gulf waters of Thailand (Brunckhorst 1993; Chavanich et al. 2013).

##### Phyllidia picta

Taxon classificationAnimaliaNudibranchiaPhyllidiidae

Pruvot-Fol, 1957

28E67061-63FF-5C28-AD1C-56A4ABAFFBEF

Figure 15A

###### Material examined.

Two specimens 28 mm, CB; one specimen 33 mm, TB.

###### Ecology.

Abundant in coral reef habitats. Depth 3–30 m

###### Distribution.

Known mostly from South-East Asia and the western Pacific including Malaysia, the Philippines, Indonesia, Hong Kong, Papua New Guinea, Australia, Japan, Fiji, Vanuatu, the Solomon Islands (Gosliner et al. 2008) and known from both Andaman and Gulf waters of Thailand (Chavanich et al. 2013). Indian Ocean records are limited but include Socotra Island and Christmas Island (Yonow 1996, 2012).

###### Remarks.

External identification of some specimens yielded the incorrect inclusion of *Phyllidia marindica* (Yonow & Hayward, 1991) by Mehrotra and Scott (2016) from Koh Tao. More intensive surveys have yielded no specimens that externally match *P. marindica*, which appears to be limited to the Indian Ocean. Brunckhorst (1993) recorded this species from the Andaman coast of Thailand and later Chavanich et al. (2013) included the species from the Gulf of Thailand. Collections of *P. marindica* from the Gulf of Thailand are needed to firmly establish its presence within the Gulf and therefore indicating its range as one not exclusive to the Indian Ocean.

**Figure 15.**
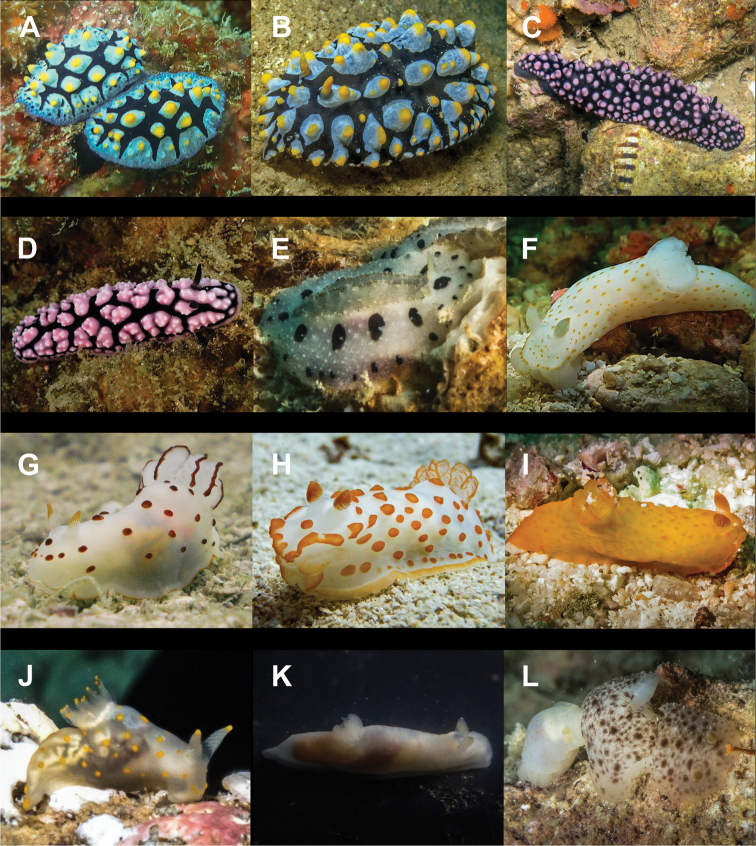
**A***Phyllidia picta* specimens 28 mm **B***Phyllidia varicosa* 55 mm (photograph by Pau Urgell Plaza) **C***Phyllidiella nigra* 50 mm **D**Phyllidiella cf. pustulosa 35 mm **E***Phyllidiopsis loricata* 28 mm **F**Gymnodoris cf. alba 25 mm (photograph by Tine Kvamme) **G***Gymnodoris ceylonica* 35 mm **H***Gymnodoris impudica* 60 mm **I***Gymnodoris inornata* 28 mm (photograph by Pau Urgell Plaza) **J***Gymnodoris* sp. 1 25 mm (photograph by Pim Bontenbal) **K, L***Gymnodoris* sp. 2 14 mm, same specimen feeding on *Dendrodoris elongata*.

##### Phyllidia varicosa

Taxon classificationAnimaliaNudibranchiaPhyllidiidae

Lamarck, 1801

A63966A5-572D-522C-8AC9-1994945185A3

Figure 15B

###### Material examined.

One specimen 32 mm, HWB; one specimen 55 mm, SR.

###### Ecology.

Abundant in coral reef habitats. Depth 3–30 m.

###### Distribution.

Widespread in the Indo-Pacific including the Red Sea (Yonow 1996), South Africa (Gosliner 1987), Tanzania (Edmunds 1971), Mozambique (Tibiriçá et al. 2017), Madagascar (Risbec 1928), Socotra, Kenya, Mauritius, Zanzibar, Seychelles, Maldives (Yonow 2012),Chagos Islands (Yonow et al. 2002), Sri Lanka, Hawaii (Debelius 1996), India (Apte 2009), Myanmar (Sanpanich and Duangdee 2019), Philippines, Vietnam (Brunckhorst 1993), Indonesia (Yonow 2011), Australia (Nimbs and Smith 2016), Palau, Japan, Papua New Guinea (Gosliner et al. 2008), and known from both Andaman and Gulf waters of Thailand (Brunckhorst 1993; Chavanich et al. 2013).

##### Genus *Phyllidiella* Bergh, 1869

##### Phyllidiella nigra

Taxon classificationAnimaliaNudibranchiaPhyllidiidae

(van Hasselt, 1824)

142B9804-D892-5BB9-8CEC-EE682B1634B3

Figure 15C

###### Material examined.

One specimen 50 mm, AMN; one specimen 50 mm, TB.

###### Ecology.

Abundant in coral reef habitats. Depth 3–30 m.

###### Distribution.

Widespread in the Indo-Pacific including the Philippines, Australia, Papua New Guinea (Brunckhorst 1993), Myanmar (Sanpanich and Duangdee 2019), Malaysia, Vietnam, Indonesia, Japan, Palau, Guam (Gosliner et al. 2008), and known from both Andaman and Gulf waters of Thailand (Swennen et al. 2001; Chavanich et al. 2013).

##### Phyllidiella cf. pustulosa

Taxon classificationAnimaliaNudibranchiaPhyllidiidae

(Cuvier, 1804)

AED72065-F5A7-5173-BFA9-BBFF6C3EA9E4

Figure 15D

###### Material examined.

Two specimens 35–40 mm, TT; one specimen 35 mm, CB.

###### Ecology.

Abundant in coral reef habitats. Depth 3–30 m.

###### Distribution.

‘*Phyllidiella pustulosa*’ is known from India (Apte 2009), the Red Sea, Indonesia, the Philippines, Japan, Papua New Guinea, Australia, Fiji (Brunckhorst 1993), Hawaii (Gosliner et al. 2008), both Andaman and Gulf waters of Thailand (Brunckhorst 1993; Chavanich et al. 2013), and an externally different morphotype referred to as ‘Phyllidiella cf. pustulosa’ is known from Mozambique (Tibiriçá et al. 2017).

###### Remarks.

Morphological and molecular work (Chang and Willan 2015; Stoffels et al. 2016) has indicated that *P. pustulosa* is composed of a number of cryptic species. Specimens recorded from Koh Tao externally look similar to those illustrated in Brunckhorst (1993: pl. 5E, F) and Stoffels et al. (2016: figs 11I, J, 12F, 13B) but dissimilar from specimens in Mozambique (Tibiriçá et al. 2017: figs 20C, D).

##### Genus *Phyllidiopsis* Bergh, 1876

##### Phyllidiopsis loricata

Taxon classificationAnimaliaNudibranchiaPhyllidiidae

(Bergh, 1873)

DCFDFFD7-F193-57D0-8379-548B9698D409

Figure 15E

###### Material examined.

One specimen 28 mm, SB; one specimen 15 mm, CP.

###### Ecology.

Under and among rocks and coral rubble. Depth 3–30 m.

###### Distribution.

Across the Indo-Pacific including Australia, Guam, Marshall Islands, Tahiti (Brunckhorst 1993), western Indian Ocean of Réunion, Papua New Guinea, Hawaii (Gosliner et al. 2008) and Singapore (Lim and Chou 1970). First documented from the Gulf of Thailand by Mehrotra and Scott (2016).

#### Superfamily Polyceroidea Alder & Hancock, 1845

**Family Polyceridae Alder & Hancock, 1845**

##### Genus *Gymnodoris* Stimpson, 1855

###### Gymnodoris cf. alba

Taxon classificationAnimaliaNudibranchiaPolyceridae

(Bergh, 1877)

56F35CD2-84B8-51C6-8CEC-0CE63BAE3D47

Figure 15F

####### Material examined.

One specimen 25 mm, SI.

####### Ecology.

Among coral and rubble in coral reef habitats. Depth 4–18 m.

####### Distribution.

*Gymnodoris alba* is currently believed to be found across the Indo-Pacific, from the Red Sea (Yonow 2008), Australia (Nimbs and Smith 2016), South Africa, the Philippines, Japan, Papua New Guinea, and Hawaii (Gosliner et al. 2008). *Gymnodoris alba* is also known from both Andaman and Gulf waters of Thailand (Chavanich et al. 2013).

####### Remarks.

Similar to *Gymnodoris alba* (Bergh, 1877) but more observations and material are needed to confidently ascertain an identification. A clarification of the *G. alba* species complex and indeed a revision of the genus is sorely needed to provide a biogeographical and taxonomic understanding of *Gymnodoris* in the Indo-Pacific. This species was first recorded as *Gymnodoris* sp. by Mehrotra and Scott (2016: fig. 2E) and is distinct from specimens of *G. alba* as recorded in both Andaman and Gulf waters by Chavanich et al. (2013).

###### Gymnodoris ceylonica

Taxon classificationAnimaliaNudibranchiaPolyceridae

(Kelaart, 1858)

4C9A1882-2131-567C-BA35-961CC2503945

Figure 15G

####### Material examined.

Two specimen 35 mm, TB.

####### Ecology.

Exclusively recorded from soft sediment habitats beyond the fringing coral reef. Depth 14 m.

####### Distribution.

Widespread in the Indo-Pacific including Mozambique (Tibiriçá et al. 2017), the Red Sea (Yonow 1990), Indonesia (Debelius 1996), India (Apte 2009), Taiwan (Huang et al. 2015), Singapore (Lim and Chou 1970), Réunion Island, Seychelles, Sri Lanka, the Philippines, Japan, Papua New Guinea, Australia, Guam, Marshall Islands (Gosliner et al. 2008), and known from both Andaman and Gulf waters of Thailand (Chavanich et al. 2013; Mehrotra and Scott 2016).

###### Gymnodoris impudica

Taxon classificationAnimaliaNudibranchiasPolyceridae

(Rüppell & Leuckart, 1830)

9C24084D-DDDC-57D2-B8DA-AAC7D7113E71

Figure 15H

####### Material examined.

One specimen 45 mm, TT; two specimens 65 mm, HF; one specimen 60 mm, TW.

####### Ecology.

In coral reef, rubble and soft sediment habitats throughout the island and nearby offshore pinnacles. Depth 5–25 m.

####### Distribution.

Widespread in the Indo-Pacific including the Red Sea (Yonow 2008), Mozambique (Tibiriçá et al. 2017), India (Ramakrishna et al. 2010), Singapore (Lim and Chou 1970), Japan (Baba 1949), South Africa, Tanzania, the Philippines, Indonesia, New Caledonia, Hawaii (Gosliner et al. 2008) and known from both Andaman and Gulf waters of Thailand (Chavanich et al. 2013).

###### * Gymnodoris inornata

Taxon classificationAnimaliaNudibranchiaPolyceridae

(Bergh, 1880)

C64388D5-2330-51A6-A657-1F8F88BA7177

Figure 15I

####### Material examined.

One specimen 28 mm, HF.

####### Ecology.

Among coral and rubble in coral reef habitats. Depth 6–12 m.

####### Distribution.

Widespread in the Indo-Pacific including Mozambique (Tibiriçá et al. 2017), China (Lin 1990), Hong Kong (Orr 1981), Japan (Baba 1949), Australia (Nimbs and Smith 2016), South Africa, Tanzania, Red Sea, Indonesia, Philippines, New Caledonia, and Hawaii (Gosliner et al. 2008). Here representing a first record for Thai waters.

###### Gymnodoris

Taxon classificationAnimaliaNudibranchiaPolyceridae

sp. 1

D5B3B7E5-E111-51ED-9658-957F415DB366

Figure 15J

####### Material examined.

One specimen 25 mm, TT.

####### Ecology.

On rocks and among coral and rubble in coral reef habitats. Depth 4–18 m.

####### Distribution.

First documented as *Gymnodoris* sp. from the Gulf of Thailand by Mehrotra and Scott (2016: fig. 2D). Similar *Gymnodoris* species (Gosliner et al. 2008: sp. 5; Gosliner et al. 2018: sp. 21) known from Japan and Papua New Guinea only.

###### * Gymnodoris

Taxon classificationAnimaliaNudibranchiaPolyceridae

sp. 2

170C6A33-F4F0-5C94-973B-43F3C1E60357

Figure 15K, L

####### Material examined.

One specimen 14 mm, CB.

####### Ecology.

Among rubble in shallow coral reef habitats, observed feeding on *Dendrodoris elongata* by progressively feeding on the extended mantle around the animal. Depth 6 m.

####### Distribution.

Unknown.

####### Remarks.

Resembling multiple species considered undescribed according to Gosliner et al. (2008, 2018). Gills white, pinnate, overall body relatively cylindrical, translucent white with numerous very small yellow-orange spots throughout the dorsum, internal viscera visible throughout. Rhinophores triangular, pale yellow.

###### * Gymnodoris

Taxon classificationAnimaliaNudibranchiaPolyceridae

sp. 3

DE558BC2-4C75-5815-B22E-DEFC2000E535

Figure 16A

####### Material examined.

One specimen 12 mm, HF.

####### Ecology.

Among rubble in coral reef habitats. Depth 6–12 m.

####### Distribution.

*Gymnodoris* sp. 36 (Gosliner et al. 2018) is known from the Philippines and Papua New Guinea.

####### Remarks.

**Figure 16.**
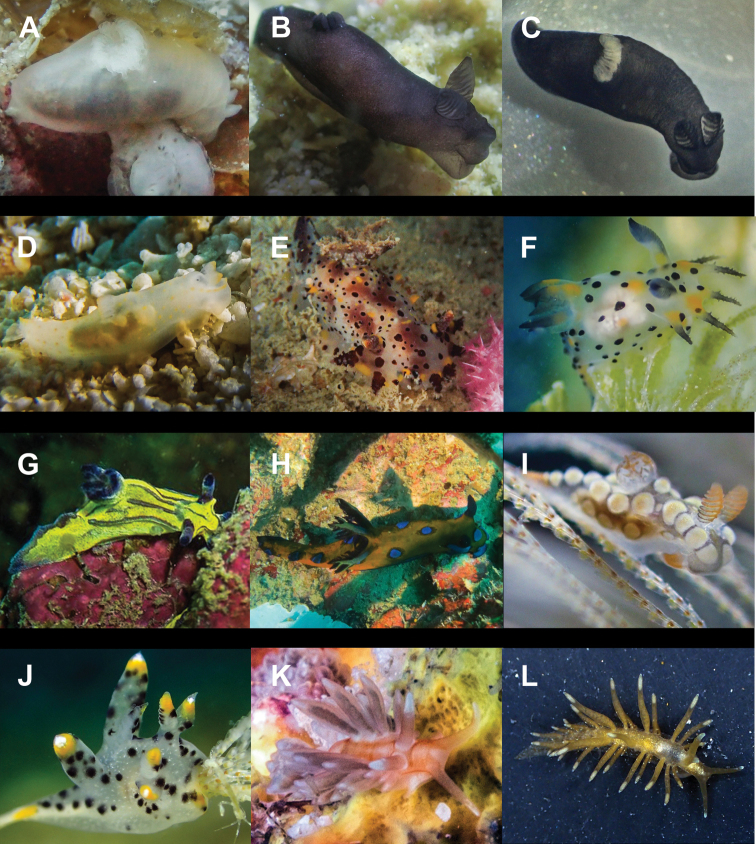
**A***Gymnodoris* sp. 3 12 mm **B***Gymnodoris nigricolor* 5 mm **C**Gymnodoris cf. nigricolor 6 mm **D***Gymnodoris* sp. 4 9 mm **E***Plocamopherus tilesii* 90 mm **F***Polycera* sp. 12 mm **G***Tambja amakusana* 6 mm (photograph by Richard Brinke) **H***Tambja pulcherrima* 40 mm (photograph by Liam Kelly) **I***Tambja* sp. 4 mm (photo by Kaitlyn Harris) **J***Thecacera* sp. 6 mm **K***Anteaeolidiella* sp. 15 mm **L***Aeolidiopsis harrietae* 17 mm.

Smooth, translucent white dorsum with club shaped gill leaves. Observed feeding on an unknown nudibranch species (Fig. 16A).

###### * Gymnodoris nigricolor

Taxon classificationAnimaliaNudibranchiaPolyceridae

Baba, 1960

1DD747CC-E471-5907-8544-84BAF26E4ADE

Figure 16B

####### Material examined.

Two specimens 5–7 mm, SN.

####### Ecology.

Recorded exclusively from deeper soft sediment habitats, crawling on the substrate. Depth 19–27 m.

####### Distribution.

*Gymnodoris nigricolor* is known from the Philippines, Japan, New Caledonia, and the Solomon Islands (Gosliner et al. 2008). Here recorded as a first for Thai waters.

####### Remarks.

The present species closely resembles externally the description by Baba (1960) possessing a black (sometimes translucent black) dorsum and rhinophores, and a translucent foot. The gills match the description in being black, small, and club-shaped and arranged in a semi-circle. It should also be noted that the plate supplementing the original description of the species shows gills in a circular arrangement and seemingly plumose rather than reduced and club shaped. While the species is known to be parasite of gobies (Osumi and Yamasu 1994), all observations from Koh Tao were not in the vicinity of any demersal fish, including any in the family Gobiidae. Ongoing searches of gobies from both soft sediment and coral reef habitats have thus far revealed no observations of parasitic nudibranchs.

###### * Gymnodoris cf. nigricolor

Taxon classificationAnimaliaNudibranchiaPolyceridae

Baba, 1960

1870FBAA-9BCB-5444-9E2B-FF5865FC624E

Figure 16C

####### Material examined.

One specimen 6 mm, SB; one specimen 7 mm, LB.

####### Ecology.

Recorded exclusively from deeper soft sediment habitats, crawling on the substrate. Depth 19–27 m.

####### Distribution.

Unknown

####### Remarks.

Strikingly similar to *Gymnodoris nigricolor* from Koh Tao in sharing habitat, size, and much of their external morphology except the arc of gills. Both observed specimens, several months and kilometres apart, were found to possess white club-shaped gills instead of black. As such we treat this species as likely distinct.

###### * Gymnodoris

Taxon classificationAnimaliaNudibranchiaPolyceridae

sp. 4

3E9E9A1F-4B0B-5E19-91E4-C2F6EFF345AD

Figure 16D

####### Material examined.

One specimen 9 mm, TW.

####### Ecology.

In soft sediment habitats beyond fringing coral reefs. Depth 12–16 m.

####### Distribution.

So far only recorded in the Gulf of Thailand.

####### Remarks.

Incorrectly identified as *Gymnodoris bicolor* (Alder & Hancock, 1864) by Mehrotra and Scott (2016) due to low quality images, additional material has shown several unique differences, including translucent club-shaped gills with yellow tips in an arc and a prominently raised area between the eyes, behind the rhinophores.

##### Genus *Plocamopherus* Rüppell & Leuckart, 1828

###### * Plocamopherus tilesii

Taxon classificationAnimaliaNudibranchiaPolyceridae

Bergh, 1877

E501145F-5CC7-5203-9B1E-697691062629

Figure 16E

####### Material examined.

Two specimens 102 mm, TT.

####### Ecology.

Exclusively recorded from soft sediment habitats beyond the fringing coral reef. Depth 18–25 m.

####### Distribution.

*Plocamopherus tilesii* is known from Turkey (Yokeş et al. 2012), Vietnam (Martynov and Korshunova 2012), the Philippines (Gosliner et al. 2008), Hong Kong (Jensen 2000), Japan (Nakano 2017), China (Gosliner and Vallès 2006), Australia (Nimbs and Smith 2016), and Korea (Song et al. 2017). Here documented as a first record for Thai waters.

##### Genus *Polycera* Cuvier, 1816

###### * Polycera

Taxon classificationAnimaliaNudibranchiaPolyceridae

sp.

176BF631-E17B-588B-BA09-C1BAA9CF0A0C

Figure 16F

####### Material examined.

One specimen 12 mm, SN.

####### Ecology.

Exclusively recorded from soft sediment habitats beyond the fringing coral reef where it feeds on Bugulidae spp. arborescent bryozoans. Depth 12–25 m.

####### Distribution.

*Polycera* sp. 1 (Gosliner et al. 2008, 2018) is known from Indonesia only. Here representing a first record for Thai waters.

##### Genus *Tambja* Burn, 1962

###### Tambja amakusana

Taxon classificationAnimaliaNudibranchiaPolyceridae

Baba, 1987

5B864D7E-0201-5CCC-BA13-366D73804C94

Figure 16G

####### Material examined.

One specimen 6 mm, CP.

####### Ecology.

Among rocks and corals on offshore pinnacle sites.

####### Distribution.

Widespread in the Indo-Pacific including Mozambique (Tibiriçá et al. 2017), Maldives (Yonow 2012), Japan (Baba 1987), Australia (Marshall and Willan 1999), Papua New Guinea (Pola et al. 2006), Vanuatu and Hawaii (Gosliner et al. 2008). First documented from the Gulf of Thailand by Mehrotra and Scott (2016).

###### Tambja pulcherrima

Taxon classificationAnimaliaNudibranchiaPolyceridae

Willan & Chang, 2017

93AF16D2-8558-5A70-ADB0-8AF70777F488

Figure 16H

####### Material examined.

One specimen 40 mm, SWP.

####### Ecology.

Locally recorded exclusively in deep soft sediment habitats at an offshore pinnacle. Depth 25–30 m.

####### Distribution.

Known from South Korea, Japan, Taiwan, Malaysia, Papua New Guinea Australia, and New Zealand (Willan and Chang 2017). First documented from the Gulf of Thailand by Mehrotra and Scott (2016).

####### Remarks.

Externally, the present species matches the species recently described by Willan and Chang (2017) and was initially documented as an unidentified species of *Tambja* by Mehrotra and Scott (2016: fig. 2H).

###### * Tambja

Taxon classificationAnimaliaNudibranchiaPolyceridae

sp.

162EF45F-CAFA-5C5E-B837-BB3510DE7408

Figure 16I

####### Material examined.

One specimen 4 mm, TT.

####### Ecology.

Recorded from soft sediment habitats beyond the fringing coral reef where it was found on Bugulidae spp. Depth 12–25 m.

####### Distribution.

*Tambja* sp. 2 (Gosliner et al. 2018) is known from the Philippines and Indonesia. Here representing a first record for Thai waters. All available photographs show this species on the arborescent bryozoans of the family Bugulidae.

##### Genus *Thecacera* J. Fleming, 1828

###### Thecacera

Taxon classificationAnimaliaNudibranchiaPolyceridae

sp.

AC26C7B8-F22B-5720-B3A3-C74669A70EC1

Figure 16J

####### Material examined.

Two specimens 6–16 mm, SB; one specimen 12 mm, KKR.

####### Ecology.

Exclusively recorded from soft sediment habitats beyond the fringing coral reef where it feeds on Bugulidae spp. arborescent bryozoans. Depth 12–25 m.

####### Distribution.

Similar species are known from Mozambique (Tibiriçá et al. 2017), Maldives (Yonow 1994), Malaysia, Indonesia, and Japan (Gosliner et al. 2008).

####### Remarks.

While similar to Thecacera cf. picta Baba, 1972 from Mozambique (Tibiriçá et al. 2017) our preliminary investigations indicate that this species is distinct from both *Thecacera picta* Baba, 1972, *Thecacera vittata* Yonow, 1994, and the supposedly pan-tropical/sub-tropical species *Thecacera pennigera* (Montagu, 1813) which was incorrectly documented from Koh Tao by Mehrotra and Scott (2016). This species is also distinct from *Thecacera pennigera* previously recorded from both Andaman and Gulf waters of Thailand by Chavanich et al. (2013).

#### Suborder Cladobranchia Willan & Morton, 1984

**Superfamily Aeolidioidea Gray, 1827**

**Family Aeolidiidae Gray, 1827**

##### Genus *Anteaeolidiella* Miller, 2001

###### * Anteaeolidiella

Taxon classificationAnimaliaNudibranchiaAeolidiidae

sp.

F17ECF75-7305-5447-A584-12FB090A849B

Figure 16K

####### Material examined.

One specimen 15 mm, CA.

####### Ecology.

Among corals and rubble in coral reef habitats. Depth 6–10 m.

####### Distribution.

*Anteaeolidiella cacaotica* (Stimpson, 1855) is known from Australia and Japan (Carmona et al. 2014b) and *Anteaeolidiella* sp. 2 (Gosliner et al. 2018) is known from Indonesia. Here representing a first record for Thai waters.

##### Genus *Aeolidiopsis* Pruvot-Fol, 1956

###### Aeolidiopsis harrietae

Taxon classificationAnimaliaNudibranchiaAeolidiidae

Rudman, 1982

3FB9112C-8FAF-5628-BF71-5C03CC64159C

Figure 16L

####### Material examined.

Two specimens 21–29 mm, LB; one specimen 17 mm, SRB.

####### Ecology.

Cryptic on their prey, *Palythoa* sp. which has thus far only been recorded growing on isolated pieces of rubble or artificial substrate (i.e., discarded plastic) in deeper soft sediment habitats outside the coral reef.

####### Distribution.

Known from Japan (Ono 2004), Australia (Rudman 1982), the Philippines and Papua New Guinea (Gosliner et al. 2008). Previously recorded from the Gulf of Thailand (Mehrotra and Scott 2016).

####### Remarks.

Specimens from Koh Tao have papillate rhinophores (similar to Carmona et al. 2014a: fig. 3D) with distinctly white tips, have between 4–6 cerata per row which are the same brown colour as the dorsum with distinctive yellowish to pale brown cnidosacs.

###### * Aeolidiopsis ransoni

Taxon classificationAnimaliaNudibranchiaAeolidiidae

Pruvot-Fol, 1956

DBF00491-B433-5558-8BE2-185FC020B980

Figure 17A, B

####### Material examined.

Two specimens 25–30 mm, SB; one specimen 23 mm, CB.

####### Ecology.

Exclusively recorded on its prey species, the zoanthid *Palythoa tuberculosa* (Esper, 1805), on which it is extremely cryptic. Depth 1–18 m.

####### Distribution.

Across the Pacific including Japan (Ono 2004), French Polynesia (Pruvot-Fol 1956), Hawaii (Carmona et al. 2014a), the Philippines, Australia, and Papua New Guinea (Gosliner et al. 2008). Here representing a first record for Thai waters.

####### Remarks.

Separated from *Aeolidiopsis harrietae* and *A. palythoae* (Gosliner, 1985) by the presence of smooth rhinophores and highly elongate body with between 17–23 pairs of cerata. Unlike specimens described by Carmona et al. (2014b) but similar to those described by Rudman (1982), specimens from Koh Tao have rhinophores that lack white tips.

**Figure 17.**
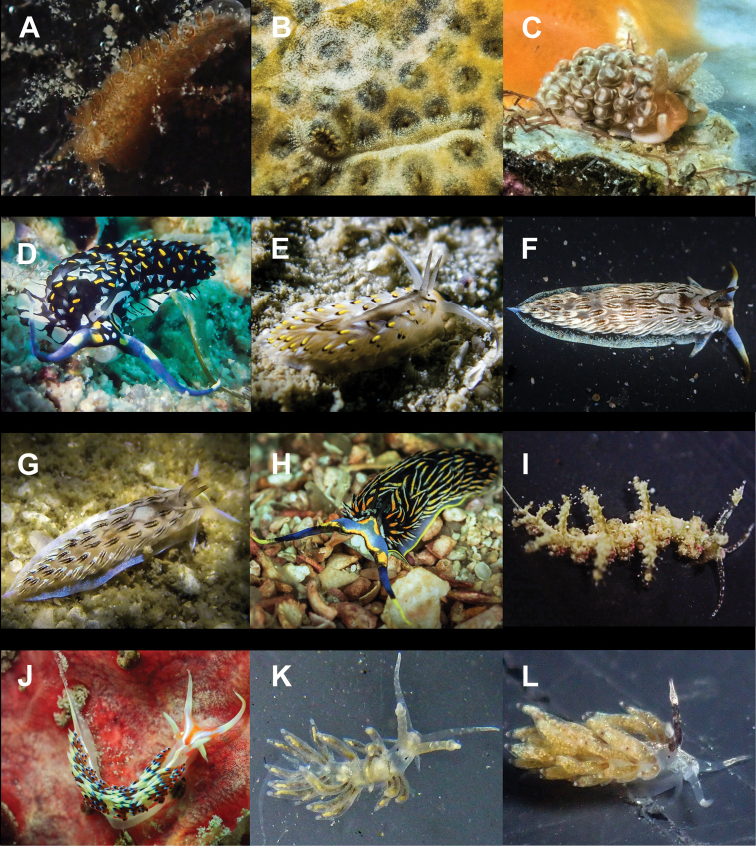
**A, B***Aeolidiopsis ransoni* 23 mm (**A**) and 25 mm (**B**) **C***Baeolidia salaamica* 10 mm **D***Cerberilla ambonensis* 20 mm **E***Cerberilla asamusiensis* 8 mm (photograph by Pau Urgell Plaza) **F, G**Cerberilla cf. incola variants 12 mm (**F**) and 7 mm (**G**) **H***Cerberilla* sp. 22 mm **I***Limenandra confusa* 12 mm **J***Caloria indica* 26 mm **K***Favorinus* sp. 1 5 mm **L***Favorinus* sp. 2 5 mm

##### Genus *Baeolidia* Bergh, 1888

###### * Baeolidia salaamica

Taxon classificationAnimaliaNudibranchiaAeolidiidae

(Rudman, 1982)

99904634-9DA1-56B0-A746-6051E84CB3A7

Figure 17C

####### Material examined.

One specimen 10 mm, CA.

####### Ecology.

Among corals and rubble in coral reef habitats. Depth 6–10 m.

####### Distribution.

Across the Indo-Pacific including Tanzania (Rudman 1982), Japan (Ono 2004), Korea (Koh 2006), Hawaii (Carmona et al. 2014a), Philippines, and Papua New Guinea (Gosliner et al. 2008). Here representing a first record for Thai waters.

####### Remarks.

Rhinophores with numerous small white knobs leading to white apices, a faint white ring visible on the head, anterior to the rhinophores. Foot white and wide. Oral tentacles basally translucent with white tips, often held curled closer to the head (Fig. 17C) but longer than rhinophores when extended. Cerata moderately long, brown with white speckles with very distinctive white cnidosacs. A similar brown colour (though often more orange) is the main colouration of the dorsum, head, and rhinophores, under the aforementioned white pigmentation, with some faint white pigmentation anterior and posterior to the pericardium. This brownish orange colouration appears to be the main externally obvious difference between the specimens from Koh Tao and those described by Carmona et al. (2014b), but orange pigmentation was described in the original description of the species, particularly in relation to the rhinophores.

##### Genus *Cerberilla* Bergh, 1873

###### Cerberilla ambonensis

Taxon classificationAnimaliaNudibranchiaAeolidiidae

Bergh, 1905

7AD4E17D-2152-5AA3-A413-79703BE1B8D2

Figure 17D

####### Material examined.

One specimen 10 mm, TT; one specimen 20 mm, SN.

####### Ecology.

Exclusively found in deeper soft sediment habitats outside coral reef habitats, where it exhibits an endo-benthic substrate preference. Depth 14–20 m.

####### Distribution.

Across the Indo-Pacific including Mozambique (Tibiriçá et al. 2017), India (Ramakrishna et al. 2010), Indonesia, Australia, and the Solomon Islands (Gosliner et al. 2008). First documented from the Gulf of Thailand by Mehrotra and Scott (2016).

###### Cerberilla asamusiensis

Taxon classificationAnimaliaNudibranchiaAeolidiidae

Baba, 1940

9AF4FE03-6A0C-5290-8C17-AB67E23E5617

Figure 17E

####### Material examined.

One specimen 8 mm, TT.

####### Ecology.

Exclusively found in deeper soft sediment habitats outside coral reef habitats, where it exhibits an endo-benthic substrate preference. Depth 14–20 m.

####### Distribution.

Across the Indo-Pacific including Australia (Nimbs and Smith 2016), China (Lin 1990), South Korea (Koh 2006), Japan (Baba 1957), and Indonesia (Gosliner et al. 2018). First documented from the Gulf of Thailand by Mehrotra and Scott (2016).

###### * Cerberilla cf. incola

Taxon classificationAnimaliaNudibranchiaAeolidiidae

Burn, 1974

DF3AFFC8-7363-525A-9045-B0B92A8E6485

Figure 17F, G

####### Material examined.

Two specimens 7–12 mm, TT.

####### Ecology.

Exclusively found in deeper soft sediment habitats outside coral reef habitats, where it exhibits an endo-benthic substrate preference. Depth 14–20 m.

####### Distribution.

*Cerberilla incola* is known from Australia (Burn 1974) and Réunion Island (Bachel 2010). First documented from the Gulf of Thailand by Chavanich et al. (2013).

####### Remarks.

*Cerberilla incola* as described by Burn (1974) represents a largely brown animal with dark brown rhinophores, dark brown lines across oral tentacles and along lateral and central lines of the dorsum with central cerata tipped with dark arrow-shaped marks. A later observation from near the type locality of South-East Australia (Cobb 2010) highlighted a light brown to nearly white specimen with pale blue lines along oral tentacles, a distinctive yellow-orange band along the anterior portion of the head, and with most cerata bearing parallel lines ranging from light to dark brown. A further observation from Réunion Island in the Indian Ocean (Bachel 2010) was also considered to be *C. incola* by Rudman (2010) bearing the same parallel lines along the cerata but lacking in the yellow-orange band of the earlier observation. All animals appear to have dark brown-grey rhinophores and the same dark colour anterior to the pericardium. Specimens from Koh Tao have been observed to show external variability (Fig. 17F, G) and further investigation is required.

###### * Cerberilla

Taxon classificationAnimaliaNudibranchiaAeolidiidae

sp.

B8DA29C8-3AB2-5EED-93C0-476FDBAFB85B

Figure 17H

####### Material examined.

One specimen 22 mm, SN.

####### Ecology.

Exclusively found in deeper soft sediment habitats outside coral reef habitats, where it exhibits an endo-benthic substrate preference. Depth 14–20 m.

####### Distribution.

*Cerberilla* sp. 4 (Gosliner et al. 2018) is currently known from Indonesia.

##### Genus *Limenandra* Haefelfinger & Stamm, 1958

###### * Limenandra confusa

Taxon classificationAnimaliaNudibranchiaAeolidiidae

Carmona, Pola, Gosliner & Cervera, 2014

4ED30D86-4A6D-5B88-852C-AEA832719819

Figure 17I

####### Material examined.

One specimen 4 mm, LT; one specimen 7 mm, LB; two specimens 6–12 mm, CB.

####### Ecology.

On rocks and under rubble, including skeletons of dead fungiid corals, in coral reef habitats. Depth 2–14 m.

####### Distribution.

Until recently, known only from the Pacific including Costa Rica (Camacho-García et al. 2005), Gulf of California (Bertsch 1972), Hawaii (Kay 1979), Australia (Nimbs and Smith 2016), Mexico, and the Philippines (Gosliner et al. 2008). Recorded in the Indian Ocean from Mozambique (Tibiriçá et al. 2017) and the Red Sea (Yonow 2015). Here documented as a first record for Thai waters.

#### Family Facelinidae Bergh, 1889

**Genus *Caloria* Trinchese, 1888**

##### Caloria indica

Taxon classificationAnimaliaNudibranchiaFacelinidae

(Bergh, 1896)

637B2730-91B0-52EA-9656-7204F7C9ABEE

Figure 17J

###### Material examined.

One specimen 26 mm, TW; two specimens 31–39 mm, SWP; two specimens 18–25 mm, HF.

###### Ecology.

Abundant in coral reef habitats, particularly at the numerous coral reef restoration sites at Koh Tao and at rocky pinnacle sites nearshore and offshore. Depth 2–30 m.

###### Distribution.

Widespread across the Indo-Pacific including Mozambique (Tibiriçá et al. 2017), India (Sreeraj et al. 2012), Maldives (Yonow 1994), Myanmar (Sanpanich and Duangdee 2019), Papua New Guinea (Baine and Harasti 2007), South Africa, Tanzania, Indonesia, Australia, Japan, Hawaii (Gosliner 1987), Oman, Seychelles, New Caledonia, Solomon Islands, Fiji (Gosliner et al. 2008) and documented from both Andaman and Gulf waters of Thailand by Chavanich et al. (2013).

##### Genus *Favorinus* Gray, 1850

##### * Favorinus

Taxon classificationAnimaliaNudibranchiaFacelinidae

sp. 1

816EFE20-A510-5D3B-9380-9F5ADB40AE7E

Figure 17K

###### Material examined.

One specimen 5 mm, CB; one specimen 5 mm, MH.

###### Ecology.

Documented in habitats ranging from shallow reef rubble and soft sediments to deeper soft sediment habitats. Depth 5–18 m.

###### Distribution.

*Favorinus* sp. 8 (Gosliner et al. 2018) is known from Papua New Guinea and Palau. Here documented as a first record for Thai waters.

###### Remarks.

Similar to *Favorinus* sp. 8 in Gosliner et al. (2018) which they record as feeding on the eggs of *Stylocheilus striatus* under ex-situ conditions. While the prey of the present species has yet to be documented, its range almost directly overlaps with the range of *S. striatus* from Koh Tao, which grazes on cyanobacteria in shallow and deeper soft sediment habitats at sites CB and SRB.

##### * Favorinus

Taxon classificationAnimaliaNudibranchiaFacelinidae

sp. 2

1490924A-DC4B-50B1-A6C4-EC7BD08EF566

Figure 17L

###### Material examined.

Two specimens SB, 5 mm.

###### Ecology.

Documented from deeper soft sediment habitats only. Depth 18–25 m.

###### Distribution.

*Favorinus* sp. 4 and *Favorinus* sp. 12 (Gosliner et al. 2018) are known from the Philippines. Here documented as a first record for Thai waters.

##### * Myja cf. longicornis

Taxon classificationAnimaliaNudibranchiaFacelinidae

Bergh, 1896

85C358ED-E8E5-5DCE-AD7C-E68E6F559520

Figure 18A

###### Material examined.

One specimen GR, 9 mm.

###### Ecology.

Found exclusively on its prey hydroid *Pennaria disticha* (Goldfuss, 1820) uncommonly found in both coral reef and deeper soft sediment habitats. Depth 8–25 m.

###### Distribution.

*Myja longicornis* is known from Indonesia (Bergh 1896), Australia (Nimbs and Smith 2016), Japan, and Papua New Guinea (Gosliner et al. 2008). A similar species Myja cf. longicornis was recently documented from the Gulf of Thailand (Martynov et al. 2019).

###### Remarks.

This genus was recently reviewed and expanded based on specimens from Japan and the Gulf of Thailand (Martynov et al. 2019). In that study, authors concluded that the studied specimens from the Gulf of Thailand have a close resemblance to the description of *M. longicornis* by Bergh, with some internal and external differences that would require examination of specimens from the type locality of Ambon. The single specimen found at Koh Tao closely resembles other specimens found in the Gulf of Thailand, and thus its species designation remains unresolved in the absence of contemporary studies of the genus from closer to the type locality.

**Figure 18.**
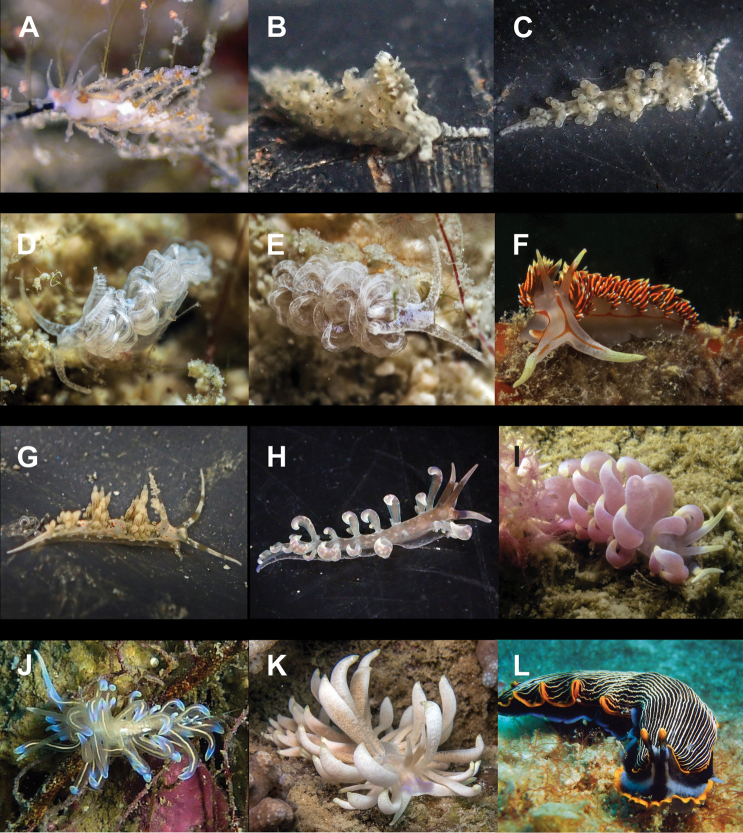
**A**Myja cf. longicornis 9 mm (photograph by Phannee Mccarthy) **B, C***Noumeaella* sp. 1 5 mm **D, E***Noumeaella* sp. 2 5 mm (photographs by Tony Myshlyaev) **F***Phidiana militaris* 35 mm (photograph by Pau Urgell Plaza) **G***Phidiana* sp. 7 mm **H***Phyllodesmium magnum* 30 mm **I**Phyllodesmium cf. magnum 35 mm (photograph by Pau Urgell Plaza) **J***Phyllodesmium opalescens* 35 mm, (photograph by Guillaume Gandoin) **K***Phyllodesmium* sp. 55 mm (photograph by Pau Urgell Plaza) **L***Armina occulta* 65 mm.

##### Genus *Noumeaella* Risbec, 1937

##### * Noumeaella

Taxon classificationAnimaliaNudibranchiaFacelinidae

sp. 1

9DB3FBF6-7EF4-56BF-8823-8DC26883DA84

Figure 18B, C

###### Material examined.

One specimen 5 mm, CB.

###### Ecology.

Found under rocks and coral rubble in shallow coral reef habitats. Depth 4–8 m.

###### Distribution.

*Noumeaella* sp. 4 is known from the Philippines (Gosliner et al. 2018). *Noumeaella rehderi* Er. Marcus, 1965 is known from Madagascar, Tanzania, Australia, Papua New Guinea, Philippines, Palau, Marshall Islands, and Hawaii (Gosliner et al. 2008). Here documented as a first record for Thai waters.

##### * Noumeaella

Taxon classificationAnimaliaNudibranchiaFacelinidae

sp. 2

D157ACE8-86FB-544E-BE1A-2F29A84DBC0D

Figure 18D, E

###### Material examined.

One specimen 5 mm, SN.

###### Ecology.

Recorded from soft sediment habitats beyond the fringing coral reef. Depth 15–20 m.

###### Distribution.

Unknown. Here documented as a first record for Thai waters.

###### Remarks.

Generally translucent throughout with some patches of white or light grey, and cerata edged in white with white apices. Bears resemblance to a number of species within the genus and requires a larger sampling effort to identify further.

##### Genus *Phidiana* Gray, 1850

##### Phidiana militaris

Taxon classificationAnimaliaNudibranchiaFacelinidae

(Alder & Hancock, 1864)

6C8467A7-AF37-50EE-AB32-197F0BAFF057

Figure 18F

###### Material examined.

Two specimens 30–35 mm, SO; two specimens 30–40 mm, HF.

###### Ecology.

Occasionally found among rubble and corals in reef habitats. Most abundant at some artificial reef sites at the island, which were initially constructed from steel rebar or concrete to support coral restoration efforts. It is likely that these substrates, while promoting scleractinian conservation, also support different potential prey items than might be found in coral reef or soft sediment habitats. Depth 8–25 m.

###### Distribution.

Across the Indo-Pacific including the Gulf of Oman (Fatemi and Attaran 2015), Red Sea (Yonow 2008), Malaysia (Ho 1989), United Arab Emirates, India, Singapore, and the Philippines (Gosliner et al. 2008). Known to have been introduced into the Mediterranean from the Red Sea (Rothman et al. 2017). Known from Andaman and Gulf waters of Thailand (Gosliner et al. 2008; Mehrotra and Scott 2016).

##### * Phidiana

Taxon classificationAnimaliaNudibranchiaFacelinidae

sp.

2712885E-C7B2-5830-A3CE-9223C9F8FBBA

Figure 18G

###### Material examined.

One specimen 7 mm, HWP.

###### Ecology.

Among rocks and coral rubble. Depth 5–16 m.

###### Distribution.

Unknown.

###### Remarks.

Bearing some similarities to *Phidiana anulifera* (Baba, 1949) and *Phidiana semidecora* (Pease, 1860).

#### Family Myrrhinidae Bergh, 1905

**Genus *Phyllodesmium* Ehrenberg, 1831**

##### Phyllodesmium magnum

Taxon classificationAnimaliaNudibranchiaMyrrhinidae

Rudman, 1991

CCA41217-64B0-5972-9B34-BDDD86F14BA4

Figure 18H

###### Material examined.

Two specimens 30–40 mm, TT.

###### Ecology.

In coral reef habitats where it feeds on the octocoral *Sinularia* sp. Depth 5–18 m.

###### Distribution.

Widespread across the Indo-Pacific including Mozambique (Tibiriçá et al. 2017), South Africa (Fraser 2001), the Red Sea (Koretz 2005), Hong Kong, New Caledonia, Australia, Marshall Islands (Rudman 1991), Japan (Ono 2004), Tanzania, Philippines, Indonesia, and Papua New Guinea (Gosliner et al. 2008). Documented from both Andaman and Gulf waters of Thailand by Chavanich et al. (2013).

##### Phyllodesmium cf. magnum

Taxon classificationAnimaliaNudibranchiaMyrrhinidae

Rudman, 1991

2E73D305-1B68-5FC8-A060-7955C5DAC411

Figure 18I

###### Material examined.

One specimen 15 mm, TT; one specimen 35 mm, SB.

###### Ecology.

In soft sediment habitats outside coral reef habitats, found regularly on or in the immediate vicinity of *Dendronephthya* octocorals, though active feeding has not yet been confirmed. Depth 16–28 m.

###### Distribution.

Unknown

###### Remarks.

The present species externally appears to match *Phyllodesmium magnum* Rudman, 1991, though smaller with relatively short cerata and no brown pigment on the animal, having pale yellow tips to rhinophores and oral tentacles. All individuals recorded to date were separately found on or very close to *Dendronephthya* spp., distant from any colonies of *Sinularia* which at Koh Tao grow only on rocks in shallower coral reef habitats. While it is possible that some animals recorded at Koh Tao are indeed *P. magnum* with differences in external colouration reflecting a drastically different diet from that known for *P. magnum*, the present species is provisionally treated as distinct due to its unique ecology.

##### * Phyllodesmium opalescens

Taxon classificationAnimaliaNudibranchiaMyrrhinidae

Rudman, 1991

3A0183DF-EAFC-5E3A-AE58-9A83858E3DF3

Figure 18J

###### Material examined.

One specimen 35 mm, CP.

###### Ecology.

Among coral, rocks, and discarded fishing equipment at an offshore pinnacle site. Depth 16 m.

###### Distribution.

Known from the Philippines, Hong Kong, Korea, and Japan (Gosliner et al. 2008). Here documented as a first record for Thai waters.

##### * Phyllodesmium

Taxon classificationAnimaliaNudibranchiaMyrrhinidae

sp.

A9971ACD-2A80-5503-A85E-CACEDB95E960

Figure 18K

###### Material examined.

Two specimens 40–55 mm, HWB.

###### Ecology.

In shallow coral reef habitats where it observed feeding on a different species of *Sinularia* than *P. magnum*. Depth 2–8 m.

###### Distribution.

*Phyllodesmium* sp. 2 (Gosliner et al. 2018) is known from Indonesia.

###### Remarks.

Similar to *Phyllodesmium* sp. 2 by Gosliner et al. (2018) which in turn is similar to *P. magnum*. External differences here are pale cerata that are less curved and held more upright than those of *P. magnum*, with digestive glands clearly visible and lacking any blueish purple pigment. Some purple-grey pigment is seen on the head.

#### Superfamily Arminoidea Iredale & O’Donoghue, 1923 (1841)

**Family Arminidae Iredale & O’Donoghue, 1923 (1841)**

**Genus *Armina* Rafinesque, 1814**

##### Armina occulta

Taxon classificationAnimaliaNudibranchiaArminidae

Mehrotra, Caballer & Chavanich, 2017

89412753-EEE1-5725-8A17-B51F1766F344

Figure 18L

###### Material examined.

Two specimens 65–72 mm, SN.

###### Ecology.

Exclusively known from soft sediment habitats outside the coral reef where it feeds on the sea pen *Virgularia* sp. Depth 14–22 m.

###### Distribution.

Known from the western Pacific including Indonesia (Yonow 2017), the Philippines (Koehler 2002), Australia (Hatton 2019), Papua New Guinea (Adams 2000), and Palau (Gosliner et al. 2008). Known from the Gulf of Thailand (Mehrotra et al. 2017). Koh Tao is the type locality of this species.

##### Armina scotti

Taxon classificationAnimaliaNudibranchiaArminidae

Mehrotra, Caballer & Chavanich, 2017

4A67AF78-C0F7-5F20-94FB-B55BCD92C115

Figure 19A

###### Material examined.

Two specimens 35–49 mm, SB; one specimen 41 mm, TT.

###### Ecology.

Exclusively known from soft sediment habitats outside the coral reef where it feeds on the sea pen *Virgularia* sp. One individual observed feeding on partially decomposed *Pteroeides* sp. (Octocorallia: Pennatulidae). Depth 12–29 m.

###### Distribution.

Known from the western Pacific including Japan (Okutani 2000), Singapore, the Philippines, and Indonesia (Gosliner et al. 2008). Known from the Andaman and Gulf waters of Thailand (Chavanich et al. 2013; Mehrotra et al. 2017). Koh Tao is the type locality of this species.

###### Remarks.

Specimens identified as *Armina semperi* (Bergh, 1866) from coasts of Thailand correspond to this species (Mehrotra et al. 2017).

**Figure 19.**
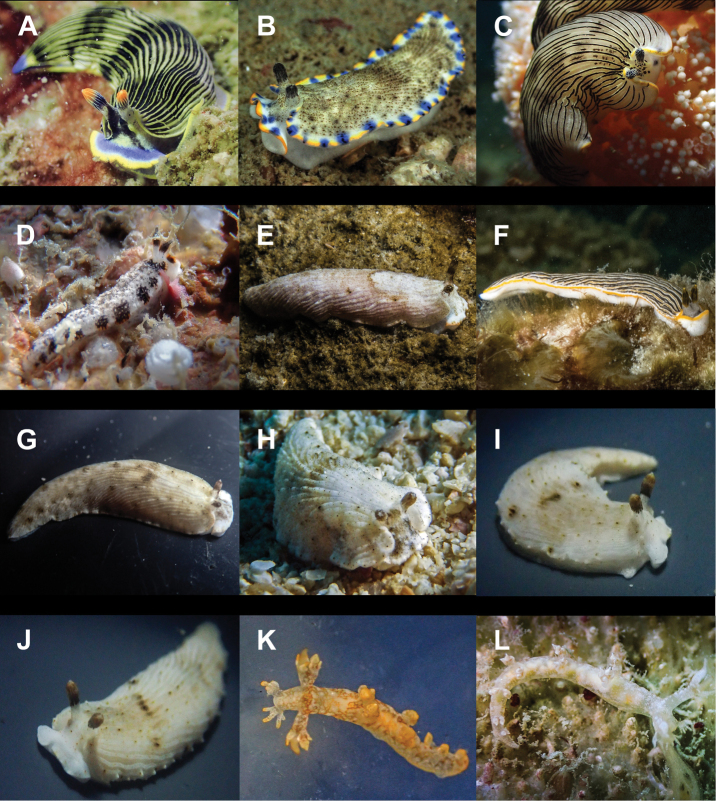
**A***Armina scotti* 41 mm **B***Dermatobranchus caeruleomaculatus* 55 mm **C***Dermatobranchus dendronephthyphagus* 40 mm **D***Dermatobranchus fortunatus* 11 mm **E***Dermatobranchus semilunus* 33 mm (photograph by Pau Urgell Plaza) **F**Dermatobranchus cf. striatus 14 mm **G, H***Dermatobranchus* sp. 1 32 mm (**G**) and 28 mm (**H**) **I, J***Dermatobranchus* sp. 2 27 mm **K***Bornella hermanni* 12 mm **L***Bornella johnsonorum* 35 mm.

##### Genus *Dermatobranchus* van Hasselt, 1824

##### * Dermatobranchus caeruleomaculatus

Taxon classificationAnimaliaNudibranchiaArminidae

Gosliner & Fahey, 2011

59C00D86-261E-53F8-AA09-BE31BB62288E

Figure 19B

###### Material examined.

Two specimens 55–67 mm, SN.

###### Ecology.

Exclusive to the soft sediment habitats outside the coral reef. Depth 14–24 m.

###### Distribution.

Known from the western Pacific including Malaysia, Indonesia, and the Philippines (Gosliner et al. 2008), Papua New Guinea (Coleman 2008), and Japan (Nakano and Fujii 2014). Here documented as a first record for Thai waters.

##### * Dermatobranchus dendronephthyphagus

Taxon classificationAnimaliaNudibranchiaArminidae

Gosliner & Fahey, 2011

EC94CC40-F130-5BF4-8C4A-2275ABBC8139

Figure 19C

###### Material examined.

Two specimens 40–50 mm, SB.

###### Ecology.

Exclusive to the soft sediment habitats outside the coral reef where it may be found feeding on *Dendronephthya* sp. or partially buried in the nearby silt/sand. Depth 14–24 m.

###### Distribution.

Known from the West Pacific including Japan and Australia (Rudman 2005b; Nimbs and Smith 2016). Here documented as a first record for Thai waters.

###### Remarks.

Recorded as *Dermatobranchus* sp. by Mehrotra and Scott (2016: fig. 3C).

##### Dermatobranchus fortunatus

Taxon classificationAnimaliaNudibranchiaArminidae

(Bergh, 1888)

D5EEE362-659A-5DAF-9C9F-E67CA1E7B717

Figure 19D

###### Material examined.

Two specimens 7–11 mm, CB; one specimen 9 mm, SB.

###### Ecology.

On rocks and under rubble, in particular under skeletons of dead Fungiidae corals, in shallow coral reef habitats. Depth 1–18 m.

###### Distribution.

Across the Indo-Pacific including the Indian Ocean of Java (Bergh 1888), India (Dixit et al. 2017), Japan (Bolland 2003), Australia (Marshall and Willan 1999), the Seychelles, the Philippines, Malaysia, Indonesia, and Papua New Guinea (Gosliner and Fahey 2011). Documented from the Gulf waters of Thailand by Mehrotra and Scott (2016).

##### * Dermatobranchus semilunus

Taxon classificationAnimaliaNudibranchiaArminidae

Gosliner & Fahey, 2011

961ED882-0417-5A6B-8F5F-6626AFDEBC6D

Figure 19E

###### Material examined.

One specimen 33 mm, TB.

###### Ecology.

Exclusive to the soft sediment habitats outside the coral reef. Observed on *Dendronephthya* sp. octocoral, though active feeding was not observed. Depth 14–24 m.

###### Distribution.

Known from the West Pacific including Malaysia, the Philippines, Indonesia, and Papua New Guinea (Gosliner and Fahey 2011). Here documented as a first record for Thai waters.

###### Remarks.

Specimens from Koh Tao are sometimes found with a pale yellow-pink margin to the oral veil. A similar trait is known from the closely related *Dermatobranchus fasciatus*Gosliner and Fahey 2011; however, the present specimens externally match *D. semilunus* based on other characteristics. Additionally, similar pigment appears to be visible in a photograph of a specimen in the original description of the species (Gosliner and Fahey 2011: fig. 74C).

##### Dermatobranchus cf. striatus

Taxon classificationAnimaliaNudibranchiaArminidae

van Hasselt, 1824

6CB733D7-4415-50AF-AE92-068C8FACDE83

Figure 19F

###### Material examined.

Two specimens 9–12 mm, SI; two specimens 11 mm, HPW; one specimen 14 mm, SW.

###### Ecology.

On rocks in coral reef habitats where it feeds on the octocoral *Briareum stechei* (Kükenthal, 1908). Occasionally observed among colonies of *B. stechei* growing on artificial substrates (i.e., discarded fishing nets) in deeper soft sediment habitats. Depth 6–22 m.

###### Distribution.

*Dermatobranchus striatus* is known from Indonesia, Papua New Guinea, and Japan (see Gosliner and Fahey 2011).

###### Remarks.

This species was recorded as *Dermatobranchus striatus* van Hasselt, 1824 by Mehrotra and Scott (2016). Due to taxonomic uncertainty between this and similar species (see Gosliner and Fahey 2011), and with *D. striatus* being the type species for the genus, we refer to this species as D. cf. striatus.

##### * Dermatobranchus

Taxon classificationAnimaliaNudibranchiaArminidae

sp. 1

64555926-3499-5774-ABFD-2FC62B1C9FA1

Figure 19G, H

###### Material examined.

Three specimens 28–40 mm, SB.

###### Ecology.

Exclusive to the soft sediment habitats outside the coral reef where it feeds on colonies of the octocoral *Dendronephthya* sp. Depth 14–24 m.

###### Distribution.

Unknown.

###### Remarks.

A species that vaguely resembles but is distinct from *Dermatobranchus semilunus* is regularly recorded from the same habitats and locations as other soft-sediment dwelling members of the genus. Specimens of *Dermatobranchus* sp. 1 externally appear to have characteristics of *D. fasciatus* and *D. semilunus*. All specimens have prominent longitudinal ridges on a generally white dorsum, with numerous black spots of varying sizes distributed along the ridges and margin of the oral veil. The oral veil always has patches of grey and the margin is sometimes pigmented with a yellow-pink band which is often pale or completely absent in some specimens. The dorsal surface usually has a single horizontal diffuse band approximately one third of the total animal length. The foot is pale pink to white, sometimes with numerous small black spots. The rhinophores have white tips, dark clubs with white lines along the lamellae, and white stalks with dark grey pigment along the inner edge of the stalks often forming a dark grey band in between and anterior to the rhinophores.

##### * Dermatobranchus

Taxon classificationAnimaliaNudibranchiaArminidae

sp. 2

CF9A0102-3637-5B8F-BD12-8D5BF04AFFA7

Figure 19I, J

###### Material examined.

One specimen 27 mm, TT.

###### Ecology.

Exclusive to the soft sediment habitats outside the coral reef. Depth 21 m.

###### Distribution.

Unknown.

###### Remarks.

*Dermatobranchus* sp. 2 is characterised by prominent pale yellow marginal sacs that are very visible as conical papillae. The dorsal longitudinal ridges and grooves are white to pale brown and scattered sparsely with small brown spots of varying sizes. These spots extend to the oral veil, which is noticeably whiter than the dorsum. The rhinophore stalks are translucent white followed by a sharp black band at the base of the rhinophore club. This fades into brown, becoming paler apically with a translucent white apex. The anterior foot corners are blunt and short, with the foot being white. This species resembles *D. fasciatus* but differs in lacking any marginal pigmentation on either mantle or foot, and possessing prominent marginal papillae that are not seen in *D. fasciatus*.

#### Superfamily Dendronotoidea Allman, 1845

**Family Bornellidae Bergh, 1874**

**Genus *Bornella* Gray, 1850**

##### Bornella hermanni

Taxon classificationAnimaliaNudibranchiaBornellidae

Angas, 1864

052DCEAB-36A9-5EE4-83A2-42E71DBE612C

Figure 19K

###### Material examined.

Two specimens 12–18 mm, HWB.

###### Ecology.

Among corals, rocks and under rubble in shallow coral reef habitats. Depth 2–12 m.

###### Distribution.

Across the Indo-Pacific including Christmas Island in the Indian Ocean, Malaysia, the Marshall Islands (Pola et al. 2009), Korea (Koh 2006), Japan (Baba 1949), and Australia (Angas 1864). First recorded from the Gulf of Thailand by Mehrotra and Scott (2016).

##### * Bornella johnsonorum

Taxon classificationAnimaliaNudibranchiaBornellidae

Pola, Rudman & Gosliner, 2009

CE6C4865-9398-5C69-8B5F-B819AAEBDE7F

Figure 19L

###### Material examined.

One specimen 35 mm, CB.

###### Ecology.

On rocks and under rubble, in particular under skeletons of dead fungiid corals, in shallow coral reef habitats. Depth 2–8 m.

###### Distribution.

*Bornella johnsonorum* is known from the Marshall Islands in the Pacific (Pola et al. 2009) and maybe from Réunion Island and the Red Sea and in the western Indian Ocean (Bidgrain 2009; Yonow 2015). Here documented as a first record for Thai waters.

###### Remarks.

The present species matches the external description of the species (Pola et al. 2009) very closely, although lacking any signs of orange reticulation. Specimens from Koh Tao have six paired dorsal processes and a single, extremely small, unpaired dorsal process near the tip of the tail.

##### Bornella stellifera

Taxon classificationAnimaliaNudibranchiaBornellidae

(A. Adams & Reeve in A. Adams, 1848)

02E184C5-E19B-5639-AFA0-775EC7BCA8D5

Figure 20A

###### Material examined.

One specimen 31 mm, CB.

###### Ecology.

On rocks and under rubble, in particular under skeletons of dead Fungiidae corals, in shallow coral reef habitats. Depth 2–8 m.

###### Distribution.

Widespread across the Indo-Pacific including the Red Sea (Vayssière 1912), Mozambique (Tibiriçá et al. 2017), South Africa (Gosliner 1987), Japan (Baba 1949), Singapore, Madagascar, India, Malaysia, the Philippines, Australia, Papua New Guinea, Fiji, Hawaii (Pola et al. 2009), Indonesia, Taiwan and New Caledonia (Gosliner et al. 2008). Recorded from the Andaman and Gulf waters of Thailand (Jensen 1998; Pola et al. 2009).

**Figure 20.**
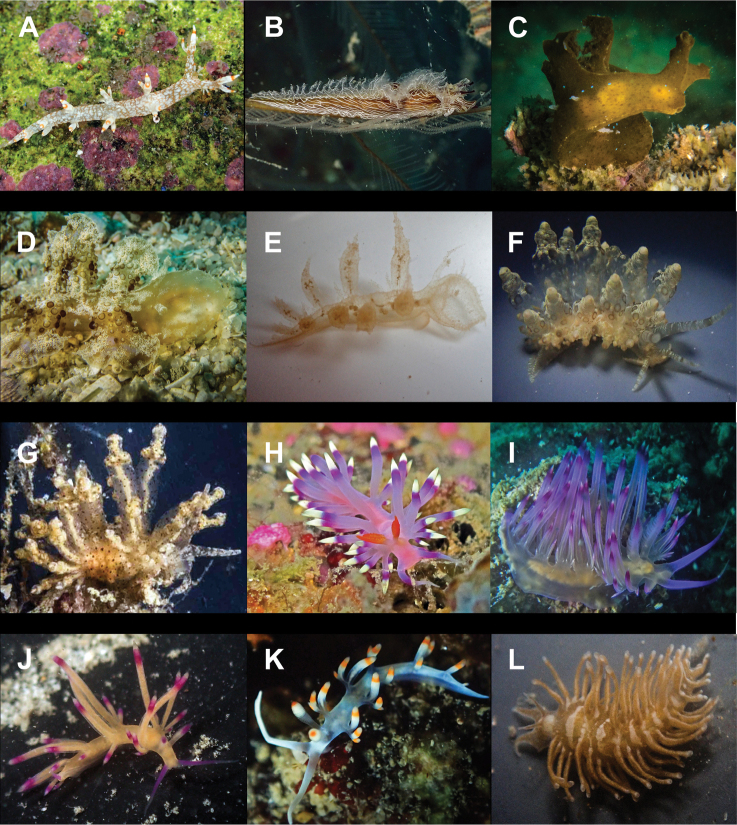
**A***Bornella stellifera* 31 mm **B***Lomanotus vermiformis* 25 mm **C***Scyllaea fulva* 45 mm **D***Melibe viridis* 97 mm **E***Melibe* sp. 22 mm **F***Eubranchus ocellatus* 22 mm **G***Eubranchus* sp. 7 mm **H***Coryphellina exoptata* 15 mm (photograph by Tine Kvamme) **I, J**Coryphellina cf. lotos variants 40 mm (**I**) and 35 mm (**J**) **K***Samla bicolor* 12 mm **L***Phestilla fuscostriata* 15 mm.

#### Family Lomanotidae Bergh, 1890

**Genus *Lomanotus* Vérany, 1844**

##### Lomanotus vermiformis

Taxon classificationAnimaliaNudibranchiaLomanotidae

Eliot, 1908

621176EC-FBA8-5C9B-9691-0250EDFF6380

Figure 20B

###### Material examined.

Two specimens 15–25 mm, SN; one specimen 17 mm, TW; one specimen 30 mm, SI.

###### Ecology.

Cryptic on its host hydroid *Macrorhynchia* sp., colonies of which are found exclusively in soft sediment habitats outside the coral reef. Depth 12–34 m.

###### Distribution.

Circumtropical, recorded from Florida, the Bahamas (Valdés et al. 2006), Panama (Collin et al. 2005), the Red Sea (Vayssière 1912), India (Dixit et al. 2017), Malaysia (Mayes 2008), the Philippines (Koehler 2005), Australia (Nimbs and Smith 2016), Indonesia, and Papua New Guinea (Gosliner et al. 2008). Recorded from the Andaman and Gulf waters of Thailand (Koehler 1999; Mehrotra and Scott 2016).

#### Family Scyllaeidae Alder & Hancock, 1855

**Genus *Scyllaea* Linnaeus, 1758**

##### * Scyllaea fulva

Taxon classificationAnimaliaNudibranchiaScyllaeidae

Quoy & Gaimard, 1824

6D09EC5C-1991-50E5-9D75-812B5D528F1E

Figure 20C

###### Material examined.

One specimen 45 mm, CB.

###### Ecology.

Recorded from a single individual on floating algae *Sargassum oligocystum* (Montagne, 1845).

###### Distribution.

Across the Indo-Pacific including Mozambique (Tibiriçá et al. 2017), Réunion Island (Cadet 2012), Japan (Baba 1949), the Philippines, Papua New Guinea (Pola et al. 2012), and maybe the Mediterranean (Yokes 2002; Pola et al. 2012). Here documented as a first record for Thai waters.

#### Family Tethydidae Rafinesque, 1815

**Genus *Melibe* Rang, 1829**

##### Melibe viridis

Taxon classificationAnimaliaNudibranchiaTethydidae

(Kelaart, 1858)

7DF16C71-4468-5CC7-98F1-782EEF923875

Figure 20D

###### Material examined.

One specimen 12 mm, TT; one specimen 122 mm, SB; one specimen 97 mm, SN.

###### Ecology.

Recorded from the soft sediment habitats outside the coral reef; however, individuals have rarely been observed swimming near the surface closer to shore. It is likely that these individuals were disturbed as no individuals have been recorded in shallower reef or sandy habitats after five years of survey. Depth 14–24 m.

###### Distribution.

Across the Indo-Pacific including Mozambique (Tibiriçá et al. 2017), Tanzania (Eliot 1904), the Red Sea (Yonow 2015), India (Parasharya and Patel 2014), Vietnam, the Philippines, Japan, and Australia (Gosliner et al. 2008). It has also recently moved into the waters of the Mediterranean (Mastrototaro et al. 2004). Recorded from the Gulf waters of Thailand (Mehrotra and Scott 2016).

##### Melibe

Taxon classificationAnimaliaNudibranchiaTethydidae

sp.

12836056-638F-5342-BD2F-CED7D38DB58C

Figure 20E

###### Material examined.

Two specimens 15–22 mm, TT.

###### Ecology.

In soft sediment habitats beyond the coral reef, grazing upon the substrate. Depth 17–22 m.

###### Distribution.

*Melibe engeli* Risbec, 1937 is known across the Indo-Pacific including Mozambique (Tibiriçá et al. 2017), the Red Sea (Burghardt and Wägele 2014), the Philippines, Indonesia, Japan, New Caledonia, and Hawaii (Gosliner et al. 2008). *Melibe* sp. 1 (Gosliner et al. 2008) is known from Indonesia only. Recorded from Koh Tao as *Melibe* sp. 1 by Mehrotra and Scott (2016: fig. 3D, E).

###### Remarks.

Externally similar to *Melibe engeli* and *Melibe* sp. 1 (Gosliner et al. 2008, 2018) in shape and some morphology, with similarities between the two also being noted by Yonow (2017). Specimens recorded from Koh Tao range in colour from near colourless to strongly golden brown, although always at least slightly transparent. The body is covered in numerous small papillae, the oral hood is able to stretch to at least half of the length of the remaining body, which have four or five pairs of cerata. Cerata are mostly ovoid to cylindrical in shape, terminating in numerous large pointed white papillae. In smaller individuals, two papillae may dominate the apex of each ceras giving them a bifurcate appearance. Most intriguingly and substantially different from the morphology of *M. engeli* are the rhinophore sheaths which lack the ‘sail-like’ appendage but instead have a single long and thin almost ‘whip-like’ extension. While the overall body shape of *M. engeli* has been shown to go through numerous changes during development (Burghardt and Wägele 2014), the specimens from Koh Tao do not entirely match *M. engeli*.

#### Superfamily Fionoidea Gray, 1857

**Family Eubranchidae Odhner, 1934**

**Genus *Eubranchus* Forbes, 1838**

##### * Eubranchus ocellatus

Taxon classificationAnimaliaNudibranchiaEubranchidae

(Alder & Hancock, 1864)

BCB0AF87-F569-5864-AF6C-8E68FCC46375

Figure 20F

###### Material examined.

One specimen 22 mm, SN.

###### Ecology.

On its prey hydroid *Idiellana pristis* Lamouroux, 1816 rare in soft sediment habitats and absent from the coral reefs of Koh Tao. Depth 12–24 m.

###### Distribution.

*Eubranchus ocellatus* is known from the Red Sea (Yonow 2008), Australia (Nimbs and Smith 2016), Tanzania, Philippines, Indonesia, and New Caledonia (Gosliner et al. 2008). Here representing a first record for Thai waters and a first record for the genus in the Gulf of Thailand.

###### Remarks.

It necessary here to clarify the brief historical records of Eubranchidae in Thai waters. Chavanich et al. (2013) recorded *Baeolidia japonica* Baba, 1933 from the Gulf of Thailand as a member of the Eubranchidae, which in fact belongs to the Aeolidiidae, as a representation of the first record of the family from Thai waters. Not mentioned in the same review, however, was the observation of *Eubranchus rubropunctatus* Edmunds, 1969 from the Andaman coast of Thailand (Neal 2010) which is believed to represent the first record of the family from Thai waters.

##### * Eubranchus

Taxon classificationAnimaliaNudibranchiaEubranchidae

sp.

A9EA95EC-82A2-5163-ACD9-EAA4A85CAE6E

Figure 20G

###### Material examined.

One specimen 7 mm, HF.

###### Ecology.

From an artificial reef structure at a reef restoration site in coral reef habitats. Depth 8–11 m.

###### Distribution.

*Eubranchus* sp. 2 (Gosliner et al. 2008) is known from Indonesia only. A similar species was also documented in India (Bhave and Apte 2011: fig. 13). Here representing a first record for Thai waters.

###### Remarks.

The present species bears numerous dark brown to black spots throughout its body with bulbous transparent cerata with the digestive gland clearly visible. The dorsal colour and that of ceratal tips is a pale yellow-brown. The rhinophores, oral tentacles, and head are colourless with numerous small white patches spread throughout.

#### Family Flabellinidae Bergh, 1889

**Genus *Coryphellina* O’Donoghue, 1929**

##### Coryphellina exoptata

Taxon classificationAnimaliaNudibranchiaFlabellinidae

(Gosliner & Willan, 1991)

9E73CE17-3776-5A6A-9635-FCCBE9CFCF58

Figure 20H

###### Material examined.

One specimen 15 mm, GR.

###### Ecology.

Among rocks and corals in coral reef habitats. Depth 5–15 m.

###### Distribution.

Widespread across the Indo-Pacific including Mozambique (Tibiriçá et al. 2017), India (Ramakrishna et al. 2010), South Africa, Réunion Island, Malaysia, the Philippines, Indonesia, Japan, Australia, Papua New Guinea, and Hawaii (Gosliner et al. 2008). Recorded from both Andaman and Gulf waters of Thailand (Chavanich et al. 2013).

###### Remarks.

This species was recently transferred to the genus *Coryphellina* in an extensive revision of the family Flabellinidae (Korshunova et al. 2017a).

##### Coryphellina cf. lotos

Taxon classificationAnimaliaNudibranchiaFlabellinidae

Korshunova et al., 2017

DD444748-A364-5FDE-A28B-24630E81C8FB

Figure 20I, J

###### Material examined.

Two specimens 35–40 mm, HWP; two specimens 35 mm, KKR.

###### Ecology.

Often feeding on hydroids growing among rocks and corals in deeper coral reef habitats and on stable substrates such as discarded nets and the remains of large terrestrial plant matter in soft sediment habitats. It is likely that the currently unknown prey hydroid of this species is able to grow in greater abundance away from shallow coral reef habitats. Depth 10–35 m.

###### Distribution.

*Coryphellina lotos* is currently known only from Japan (Korshunova et al. 2017a). A similar species is here recorded for the first time from Thai waters.

###### Remarks.

Specimens from Koh Tao strongly resemble *Coryphellina lotos* ; however, a few differences may indicate a possible cryptic species. While *C. lotos* is described as light violet with parts of the rhinophores, oral tentacles, and cerata apices as lilac to reddish lilac (Korshunova et al. 2017a: fig. 38A–E), specimens from Koh Tao appear have a background colour ranging from almost colourless to pale blue-violet, with rhinophore apices and subterminal bands on oral tentacles and cerata being a much deeper purple than the reddish violet of *C. lotos*. Most distinctive, however, is the presence of a mid-dorsal deep purple line which remains continuous in some specimens, entirely broken or limited to the oral surface in others, and completely absent in yet other specimens. The same pattern seen (if present) in the dorsal line is often mimicked laterally on both sides of some specimens. While no mention of dorsal or lateral linear pigmentation was made in the description of the species, these lines are visible in images provided supplementing the description (Korshunova et al. 2017a: fig. 38A–C). Erroneously identified as *Flabellina rubrolineata* by Mehrotra and Scott (2016), the true identity of which has recently been shown to be restricted to its type locality in the Red Sea (Ekimova et al. 2020; Yonow 2020).

#### Family Samlidae Korshunova et al., 2017

**Genus *Samla* Bergh, 1900**

##### Samla bicolor

Taxon classificationAnimaliaNudibranchiaSamlidae

(Kelaart, 1858)

A5586139-2E15-51E2-ACB0-3DB67554BCD1

Figure 20K

###### Material examined.

Two specimens 9–12 mm, CB; one specimen 18 mm, SI; one specimen 16 mm, SN.

###### Ecology.

On rocks and under rubble, including skeletons of dead Fungiidae corals, in coral reef habitats. Depth 2–14 m.

###### Distribution.

Widespread across the Indo-Pacific including the Red Sea (Yonow 2000), Mozambique (Tibiriçá et al. 2017), India (Apte 2009), Chagos Islands (Yonow et al. 2002), Myanmar (Sanpanich and Duangdee 2019), Indonesia (Eisenbarth et al. 2018), South Africa, Madagascar, Tanzania, Seychelles, Malaysia, Philippines, Hong Kong, Japan, Korea, Papua New Guinea, Australia and Hawaii (Gosliner et al. 2008). Recorded from both Andaman and Gulf waters of Thailand (Chavanich et al. 2013).

#### Family Trinchesiidae F. Nordsieck, 1972

**Genus *Phestilla* Bergh, 1874**

##### * Phestilla fuscostriata

Taxon classificationAnimaliaNudibranchiaTrinchesiidae

Hu, Zhang, Xie & Qiu, 2020

6FA69020-396C-5EA3-9044-ADF2845C1483

Figure 20L

###### Material examined.

Two specimens 11–15 mm, SRB; one specimen 12 mm, HF; one specimen 18 mm, LT.

###### Ecology.

Exclusively on its prey, the scleractinian coral *Pavona decussata* Dana, 1846, which is abundant throughout the depth range of coral reefs around Koh Tao. Depth 1–19 m.

###### Distribution.

*Phestilla fuscostriata* was previously known only from Hong Kong (Hu et al. 2020). Here we record it for the first time from Thai waters.

##### Phestilla lugubris

Taxon classificationAnimaliaNudibranchiaTrinchesiidae

(Bergh, 1870)

23465E55-8C25-5B5E-BA31-8B3F44DE76E7

Figure 21A

###### Material examined.

One specimen 30 mm, HWB; two specimens 25–30 mm, SO.

###### Ecology.

Exclusively on or in the immediate vicinity of its prey, the scleractinian coral *Porites* in coral reef habitats. Locally observed to be predating colonies of *Porites lobata* Dana, 1846, *Porites lutea* Milne Edwards, 1860, and *Porites* sp., all of which have been observed hosting the distinctive egg ribbons of the species. Depth 1–16 m.

###### Distribution.

Widespread across the Indo-Pacific including the Red Sea (Yonow 2000), Mozambique (Tibiriçá et al. 2017), India (Apte 2009), Vietnam (Risbec 1956), Indonesia (Burghardt et al. 2006), Tanzania, Madagascar, Seychelles, the Philippines, Japan, Papua New Guinea, Australia, New Caledonia, Hawaii, and the Pacific coast of North America (Gosliner et al. 2008). Recorded from both Andaman and Gulf waters of Thailand (Chavanich et al. 2013).

**Figure 21.**
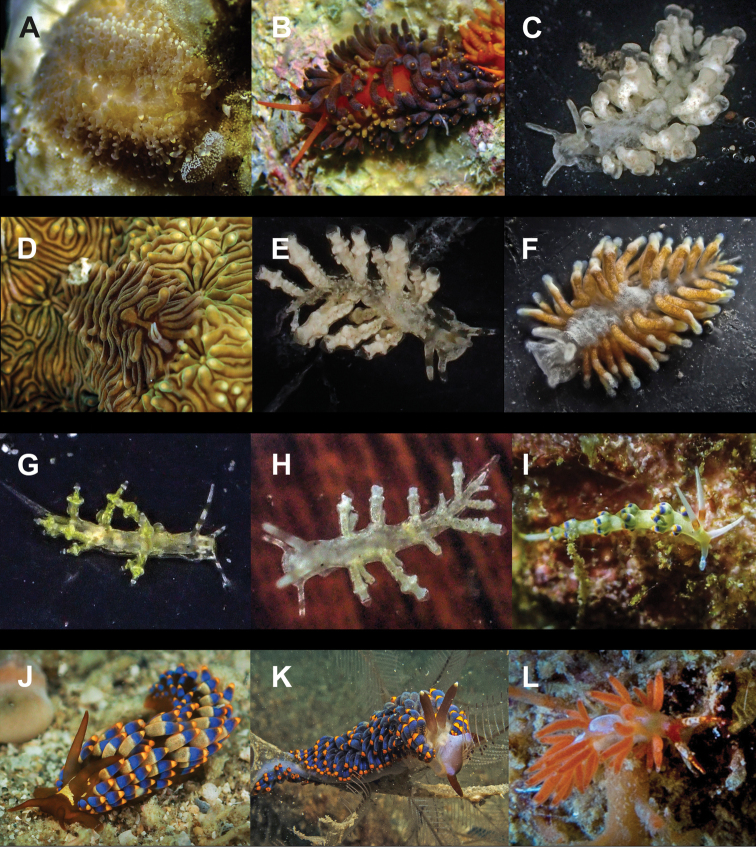
**A***Phestilla lugubris* 30 mm **B***Phestilla melanobrachia* 30 mm (photograph by Tom Jang) **C**Phestilla cf. minor 18 mm **D***Phestilla viei* 20 mm **E***Phestilla subodiosa* 2 mm **F***Phestilla* sp. 1 8 mm **G***Phestilla* sp. 2 4 mm **H***Phestilla* sp. 3 3 mm **I***Trinchesia* sp. 1 10 mm (photograph by Wanraya Kraikruan) **J***Trinchesia* sp. 2 40 mm **K***Trinchesia* sp. 3 45 mm (photograph by Pau Urgell Plaza) **L**Trinchesiidae sp. 6 mm.

##### Phestilla melanobrachia

Taxon classificationAnimaliaNudibranchiaTrinchesiidae

Bergh, 1874

A76B8107-4104-573B-9554-1853C1EE2A57

Figure 21B

###### Material examined.

Two specimens 15–30 mm, SI.

###### Ecology.

Exclusively on or in the immediate vicinity of its prey, the scleractinian corals *Tubastraea* spp., in coral reef habitats. Depth 6–32 m.

###### Distribution.

Widespread across the Indo-Pacific including Red Sea (Yonow 2000), Mozambique (Tibiriçá et al. 2017), Chagos Islands (Yonow et al. 2002), Maldives (Yonow 1994), Myanmar (Sanpanich and Duangdee 2019), Hong Kong (Scott 1984), South Africa, Réunion Island, Malaysia, the Philippines, Indonesia, Japan, Papua New Guinea, Australia, Hawaii, and Mexico (Gosliner et al. 2008). Recorded from both Andaman and Gulf waters of Thailand (Chavanich et al. 2013).

##### * Phestilla cf. minor

Taxon classificationAnimaliaNudibranchiaTrinchesiidae

Rudman, 1981

0BF9536C-C845-5C96-B7FD-888716B76ED3

Figure 21C

###### Material examined.

Four specimens 15–25 mm, CA.

###### Ecology.

Exclusively on or in the immediate vicinity of its prey, the scleractinian coral *Porites* in coral reef habitats. Locally observed to be predating colonies of *Porites lobata* and *Porites lutea*. Depth 6–14 m.

###### Distribution.

Across the Indo-Pacific including Tanzania, Australia, Hawaii (Rudman 1981), Indonesia (Burghardt et al. 2006), Madagascar, Seychelles, Philippines, Japan, Papua New Guinea, and New Caledonia (Gosliner et al. 2008). Recorded from the Gulf waters of Thailand (Chavanich et al. 2013).

###### Remarks.

Recent molecular analyses have revealed *Phestilla minor* to be a complex of up to six distinct species (Mehrotra et al. 2020a).

##### Phestilla viei

Taxon classificationAnimaliaNudibranchiaTrinchesiidae

Mehrotra, Caballer & Chavanich, 2020

8CDDCFE4-B62F-53E1-889D-A6A9D1DD1CF2

Figure 21D

###### Material examined.

Two specimens 20–25 mm, AMN; one specimen 25 mm, TT; one specimen 33 mm, SB.

###### Ecology.

Exclusively on its prey, the scleractinian coral *Pavona* in coral reef habitats. Locally observed to be predating colonies of *Pavona explanulata* Lamarck, 1816. Depth 4–22 m.

###### Distribution.

*Phestilla viei* is known from Madagascar, Thailand, Philippines, Indonesia, and Papua New Guinea (Mehrotra et al. 2020a).

##### Phestilla subodiosa

Taxon classificationAnimaliaNudibranchiaTrinchesiidae

Wang, Conti-Jerpe, Richards & Baker, 2020

A8011AB9-E96A-5C44-BB51-5D73B98E023E

Figure 21E

###### Material examined.

One specimen 2 mm, SB.

###### Ecology.

Exclusively on it its prey coral *Montipora*, in coral reef habitats. Depth 2–8 m.

###### Distribution.

*Phestilla subodiosa* is currently known from Thailand (type locality Koh Tao) and South Korea, and possibly Singapore (Wang et al. 2020).

##### * Phestilla

Taxon classificationAnimaliaNudibranchiaTrinchesiidae

sp. 1

25543D95-BC1A-5A7D-A1A7-7BEE8F608B45

Figure 21F

###### Material examined.

Two specimens 8–14 mm, CB.

###### Ecology.

Exclusively on its prey, the scleractinian coral *Goniopora* in coral reef habitats. Locally observed to be predating colonies of *Goniopora fruticosa* Saville-Kent, 1891. Depth 2–8 m.

###### Distribution.

*Phestilla* sp. 3 (Gosliner et al. 2008) is recorded from Tanzania, the Philippines, Indonesia, Japan, Hong Kong, Papua New Guinea, Australia, and the Marshall Islands. Here representing a first record for Thai waters.

##### * Phestilla

Taxon classificationAnimaliaNudibranchiaTrinchesiidae

sp. 2

62D1A7A8-457C-5B5C-8350-B5044811E079

Figure 21G

###### Material examined.

Two specimens 4–7 mm, SB.

###### Ecology.

Exclusively on its prey, the scleractinian coral *Acropora* sp. in coral reef habitats. Depth 2–8 m.

###### Distribution.

Unknown. Here representing a first record for Thai waters.

##### * Phestilla

Taxon classificationAnimaliaNudibranchiaTrinchesiidae

sp. 3

5E7F1B5B-A60C-5DE1-B23C-D3C4F05BE091

Figure 21H

###### Material examined.

One specimen 3 mm, HWB.

###### Ecology.

Recorded from a single individual found during sampling of the rare octocoral *Nanipora* (Urgell Plaza et al. 2018). The individual was recorded from the skeleton, among polyps of the coral itself. Depth 7 m.

###### Distribution.

Unknown.

###### Remarks.

Very similar to *Phestilla subodiosa*, which is considered an obligate feeder of *Montipora* sp. corals. While *Montipora* corals were observed in the vicinity, the present sample was observed upon *Nanipora*, although no feeding or egg masses were observed. The specimen has smooth rhinophores and oral tentacles with clear indications of a darker band approximately halfway on both. Cerata with clearly visible digestive glands and a distinct bulge followed by a subterminal black band (broken up into black spots in some cerata), terminating in translucent rounded apices.

##### Genus *Trinchesia* Ihering, 1879

##### Trinchesia

Taxon classificationAnimaliaNudibranchiaTrinchesiidae

sp. 1

E5C714BB-7677-50D7-90E6-E069D64327C0

Figure 21I

###### Material examined.

One specimen 10 mm, TW.

###### Ecology.

Among rocks and corals in coral reef habitats. Depth 5–10 m.

###### Distribution.

*Cuthona* sp. 2 (Gosliner et al. 2008) is known from Tanzania, the Philippines, Papua New Guinea, Japan, Australia, and Guam. Known from the Gulf of Thailand (Mehrotra and Scott 2016).

###### Remarks.

Incorrectly identified as *Cuthona ornata* Baba, 1937 by Mehrotra and Scott (2016), the present species is similar to *Cuthona* sp. 2 (Gosliner et al. 2008). The status of numerous taxa historically described under multiple families and genera such as *Cuthona*, *Trinchesia*, *Tenellia*, etc. is currently in dire need of clarification with recent attempts being made at extensive lumping of groups (Cella et al. 2016). The most recent evidence provided involved the family Trinchesiidae and genus *Trinchesia* being re-instated by Korshunova et al. (2017b). We therefore follow this (likely temporary) state of affairs until this group of taxa is stabilised.

##### Trinchesia

Taxon classificationAnimaliaNudibranchiaTrinchesiidae

sp. 2

5DE4626E-CF29-5AC3-BBC8-5AB62E7CB528

Figure 21J

###### Material examined.

Two specimens 35–40 mm, TT; one specimen 45 mm, TW.

###### Ecology.

Cryptic on its host hydroid *Macrorhynchia* sp., colonies of which are found exclusively in soft sediment habitats outside the coral reef. Depth 12–24 m.

###### Distribution.

*Tenellia* sp. 17 is known only from the United Arab Emirates (Gosliner et al. 2018) and *Tenellia* sp. (Tibiriçá et al. 2017) is known from Mozambique. Known from the Gulf of Thailand (Mehrotra and Scott 2016).

###### Remarks.

Mistakenly identified as *Cuthona yamasui* Hamatani, 1993 by Mehrotra and Scott (2016). Very similar also to *Trinchesia* sp. 3, with which it shares a prey species (alongside *Lomanotus vermiformis*).

##### * Trinchesia

Taxon classificationAnimaliaNudibranchiaTrinchesiidae

sp. 3

6662D4F6-182B-5320-B555-095ADDE3D08B

Figure 21K

###### Material examined.

Three specimens 12–45 mm, SN.

###### Ecology.

Cryptic on its host hydroid *Macrorhynchia* sp., colonies of which are found exclusively in soft sediment habitats outside the coral reef. Depth 12–24 m.

###### Distribution.

‘*Cuthona yamasui*’ (Gosliner et al. 2008) is known from Tanzania, Oman, Malaysia, the Philippines, Indonesia, Japan, and Australia. Here representing a first record for Thai waters.

###### Remarks.

Very similar to *Trinchesia* sp. 2, with which it shares a prey species (alongside *Lomanotus vermiformis*). Distinguished by the presence of a white body with brown rhinophores and oral tentacles, unlike the brown body with white markings around the rhinophores as seen in *Trinchesia* sp. 2. Cerata in the present species are basally transparent (with the blue digestive glands clearly visible) turning blue with a thin black band, a thick yellow band, and another thin black subapical band below translucent apices. The cerata of *Trinchesia* sp. 2 on the other hand are basally opaque white followed by a distinct large blue band and yellow apices with colourless tips. The present species is similar to *Tenellia* sp. 15 and *Tenellia* sp. 16 of Gosliner et al. (2018). See above comments regarding genus and family instability.

##### Trinchesiidae

Taxon classificationAnimaliaNudibranchiaTrinchesiidae

sp.

AA1AC8DC-64D4-5BDB-87D6-D474372A1F72

Figure 21L

###### Material examined.

One specimen 6 mm, HF.

###### Ecology.

On artificial reef structures feeding on the same orange *Corydendrium* sp. hydroid as *Unidentia aliciae*, on which it is cryptic. Depth 8–12 m.

###### Distribution.

*Cuthona* sp. 19 (Gosliner et al. 2008) is known from the Philippines and Papua New Guinea. Known from the Gulf of Thailand (Mehrotra and Scott 2016: fig. 2A).

#### Family Unidentiidae Millen & Hermosillo, 2012

**Genus *Unidentia* Millen & Hermosillo, 2012**

##### Unidentia aliciae

Taxon classificationAnimaliaNudibranchiaUnidentiidae

Korshunova, Mehrotra, Arnold, Lundin, Picton & Martynov, 2019

63E7FF77-25FE-5DDF-9030-9786E418264A

Figure 22A

###### Material examined.

Three specimens 19–24 mm, HF.

###### Ecology.

On artificial reef structures feeding on the same orange hydroid *Corydendrium* sp. as Trinchesiidae sp. This hydroid is predominantly known from artificial reef structures at Koh Tao (see Korshunova et al. 2019). Depth 5–18 m.

###### Distribution.

Currently known only from the Gulf of Thailand (Korshunova et al. 2019).

**Figure 22.**
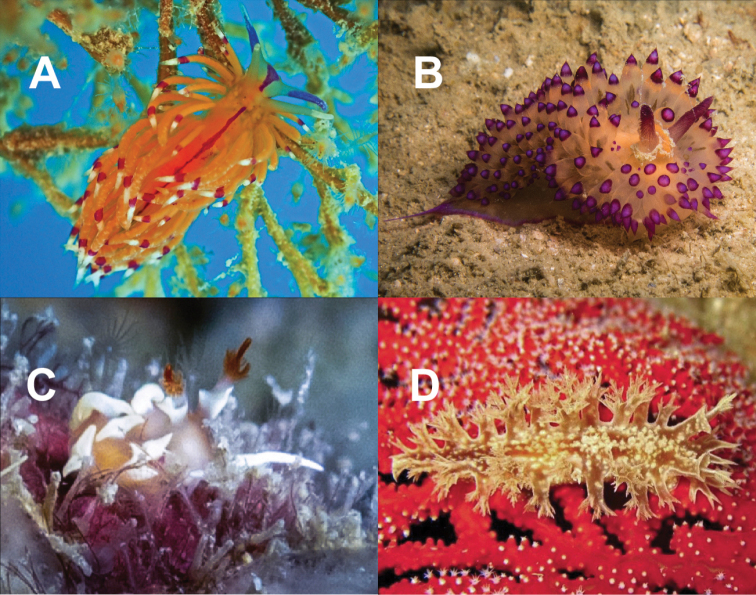
**A***Unidentia aliciae* 24 mm **B***Janolus savinkini* 55 mm (photograph by Pau Urgell Plaza) **C***Marianina rosea* 10 mm **D***Tritonia* sp. 35 mm (photograph by Khumron Waipaka).

#### Superfamily Proctonotoidea Gray, 1853

**Family Janolidae Pruvot-Fol, 1933**

##### Genus *Janolus* Bergh, 1884

###### Janolus savinkini

Taxon classificationAnimaliaNudibranchiaProctonotidae

Martynov & Korshunova, 2012

7CE9FE8D-4049-5C62-85D9-75C02BA7BB91

Figure 22B

####### Material examined.

Two specimens 55–70 mm, LB; one specimen 38 mm, SW.

####### Ecology.

In soft sediment habitats outside the coral reef. Depth 18–28 m.

####### Distribution.

Across the Indo-Pacific including the Red Sea (Yonow 2015), Australia (Nimbs and Smith 2016), Vietnam (Martynov and Korshunova 2012), the Philippines, Indonesia, and Japan (Gosliner et al. 2008). Known from the Gulf of Thailand (Mehrotra and Scott 2016).

#### Superfamily Tritonioidea Lamarck, 1809

**Family Tritoniidae Lamarck, 1809**

##### Genus *Marianina* Pruvot-Fol, 1931

###### * Marianina rosea

Taxon classificationAnimaliaNudibranchiaTritoniidae

(Pruvot-Fol, 1930)

E54A0CE8-A766-5DAE-8778-3DDC6BF8BC90

Figure 22C

####### Material examined.

One specimen 10 mm, CB.

####### Ecology.

Under rubble, among small hydroids, in coral reef habitats. Depth 5–8 m.

####### Distribution.

Across the Indo-Pacific including India (Apte 2009), Australia (Burn 1978), South Africa, Madagascar, the Philippines, Papua New Guinea, and New Caledonia (Gosliner et al. 2008). Here representing a first record for Thai waters.

####### Remarks.

The single specimen observed from Koh Tao appears paler than is typical for the species, though such colour differences are not unheard of (see Fraser 2000). It is clearly recognised by its cerata and rhinophoral morphology, unique among the Tritoniidae.

##### Genus *Tritonia* Cuvier, 1798

###### Tritonia

Taxon classificationAnimaliaNudibranchiaTritoniidae

sp.

9F25472B-F1F5-5980-8F97-6CCE5EE68175

Figure 22D

####### Material examined.

One specimen 35 mm, GR.

####### Ecology.

Known from a single specimen observed on the octocoral *Echinogorgia* sp. Depth 14 m.

####### Distribution.

*Tritonia* sp. 7 (Gosliner et al. 2008) is known only from Indonesia. Known from the Gulf of Thailand (Mehrotra and Scott 2016).

## Discussion

Prior to studies from Koh Tao, the documented diversity of sea slug taxa from the Gulf of Thailand numbered 111 species, with 204 species in total recorded for Thailand (Table 2). Surveys by Mehrotra and Scott (2016) increased these numbers to 154 for the Gulf specifically and 235 for Thai waters overall. The present findings further increase the documented diversity of sea slug taxa from the Gulf and Thai waters to 256 and 336 respectively. In general, species were recorded from coral reef habitats or soft sediment habitats exclusively, with only 28 species being found across both (Fig. 3). Of those found in coral-dominated habitats, numerous species were documented to have one of two further specialist habitat types that have thus far remained unexplained.

The first of these are those species that are documented preferentially from artificial substrates such as artificial reefs and debris. Nudibranch species such as *Phidiana militaris*, Trinchesiidae sp., and *Unidentia aliciae* were found in abundance over the years and were mostly or exclusively documented associated with prey growing on such substrates, with *U. aliciae* being described associated from these substrates (Korshunova et al. 2019). The role of artificial substrates in the benthic ecology of reef environments requires further exploration, in particular the association of sea slugs with regards to the role of synthetic materials as substrates for colonial organisms and vectors for their dispersal (Hoeksema et al. 2012; McCuller et al. 2018). Such trends may reveal unexpected ecological impacts from habitat manipulation such as coral restoration or marine debris, if they are found to promote certain organisms over others (i.e., hydroids or sponges as prey to nudibranch taxa).

The second specialist habitat type within the hard substrate habitats was the ventral surface of the skeletons of dead Fungiidae corals. These corals are known to be in high abundance around Koh Tao (Hoeksema et al. 2013; Scott et al. 2017). Benthic surveys among rubble and reef substrate environments revealed that taxa that were rarer or more cryptic due to size or camouflage were recorded in noticeably greater abundances adhering to the underside of the skeletons of dead mushroom corals than other rubble or living coral substrates in reef habitats. In particular, the species *Elysia obtusa*, *Philine orca*, Goniobranchus cf. albonares, *Mexichromis trilineata*, and *Dermatobranchus fortunatus* were all recorded almost exclusively from under these skeletons. The rugosity and proportionally larger surface area of these skeletons, in combination with the shelter from light and wave action likely provide attractive conditions for the high diversity of poriferan, algal, cnidarian, and bryozoan taxa observed under these corals. A closer investigation into the dynamics of species composition across different benthic substrate types in more coral reef areas may yield greater information on species traditionally considered ecologically cryptic.

Mehrotra and Scott (2016) found that 37% of species documented were not observed in hard substrate habitats such as fringing reefs and offshore pinnacles but were instead exclusive to deeper soft sediment habitats beyond the reef slope. Remarkably, the present findings document only 36% of all species being exclusive to these habitats, despite a more than doubling of documented taxa. These habitats support a diverse community of organisms not observed in hard substrate areas such as the fringing reef and offshore pinnacles. These include cnidarians such as pennatulacean and *Dendronephthya* sp. octocorals (Mehrotra et al. 2017), *Heteropsammia* spp. and *Heterocyathus* spp. scleractinian corals (Hoeksema and Matthews 2015; Mehrotra et al. 2016, 2019), and diverse assemblages of cerianthids and benthic hydrozoans (Martynov et al. 2019). Additionally, algae such as *Avrainvillea longicaulis* and *Vaucheria* sp. are often abundant in these areas (Mehrotra et al. 2019) as well as bryozoans of the family Bugulidae and other known prey items of sea slugs (McDonald and Nybakken 1997), most of which are either rare or entirely absent from hard substrate habitats around Koh Tao. Indeed, such ecological aspects have already been suggested as key drivers in the description of four new species from the soft sediments of Koh Tao (Mehrotra et al. 2017, 2020b).

It is possible that many of these specialised organisms can colonise much of the benthic environment in the Gulf due to its particular characteristics. Apart from being among the western-most ecological regions attributed to the Pacific, the Gulf of Thailand differs greatly from the Andaman coast of Thailand, being a region inundated by heavy sedimentation due to the many rivers that flow into it and remained entirely frozen until the glacial retreat into the Holocene (Sathiamurthy and Voris 2006). The Gulf of Thailand today has a maximum depth of 84 m (Cheevaporn and Menasveta 2003) although the vast majority of the Gulf is shallower than 75 m with only the central region exhibiting a depth of greater than this (Voris 2000; Sojisuporn et al. 2010). Therefore, the Gulf of Thailand is a 320,000 km^2^ sediment-rich basin entirely in the photic zone. It is at present challenging to draw sweeping conclusions on the comparative sea slug diversity between the Gulf of Thailand and other West-Pacific regions, as it is extremely unlikely that the majority of taxa present within either the Gulf or the surrounding seas have been documented. Nonetheless, recent years have seen attempts being made to quantitatively compare diversity estimates between West-Pacific regions (i.e., Eisenbarth et al. 2018; Undap et al. 2019). However, despite an increase in the numbers of sea slug biodiversity inventories in the past decade, there remains a paucity of in-depth ecological information for most species.

Beyond the habitat preferences, the present study further expands on the trophic dynamics of different sea slugs allowing insights into their place in the food webs of Koh Tao.

For example, predation upon sea slugs were documented from both habitat types, with predation upon Haminoeidae spp. in particular observed numerous times in the present study. In soft sediment habitats, predation by crustaceans (both decapod and stomatopod) appeared prominent (Fig. 6G), and in coral reef habitats, predation by Labriidae fish was abundant, in both cases agreeing with growing evidence of sea slugs as viable prey items to non-heterobranchs in the Indo-Pacific tropics (Mehrotra et al. 2019; Anker and Ivanov 2020). Furthermore, habitat-specifics of various prey items were distinctly visible, with certain groups (i.e., hydroids) having a much more complex habitat distribution across coral reef and soft sediment habitats than others such as major anthozoan groups. A deeper exploration of these observations is needed to investigate ecological drivers for such habitat distribution.

## Conclusions

The findings presented here highlight the need for a greater documentation and understanding of sea slug ecology in the Indo-Pacific as many questions remain regarding the habitat and prey preferences of the majority of species documented from the Gulf of Thailand and elsewhere. It is apparent that the diversity of sea slug taxa in the Gulf of Thailand has been greatly under-reported and that the marine habitats in the region support a high diversity of benthic species. With the increasing availability of SCUBA infrastructure around the Gulf, it is likely that a greater area within the region will be made accessible for the study of marine benthic environments. In conjunction, the expansion of surveys into deeper soft sediment habitats will likely further expand on the known diversity of a great number of species in these areas. Very little has been documented on the distinct biological and ecological characteristics of marine soft sediment habitats (Wilson 1991), yet it has been shown that they are able to support a USD$150 million tourism industry in South-East Asia alone (De Brauwer et al. 2017). A combination of increased efforts on surveying unexplored benthic habitats and the growing utility of citizen science efforts will yield much needed advancements in the understanding of benthic ecology in the Gulf of Thailand.
